# OTUD5-TIF1γ-SMAD3/4 positive feedback loop inhibits TGF-β-induced EMT and metastasis in NSCLC

**DOI:** 10.1038/s41419-026-08901-z

**Published:** 2026-05-25

**Authors:** Yong Wang, Runfeng Sun, Xiaochen Wang, Ersuo Jin, Lingji Hu, Xia Liu, Jie Huang, Xiaoyu Yin, Hao Shi, Chang Li, Jie Zhu, Guang Hu, Shengjie Wang, Jun Zhao, Hong-Tao Zhang

**Affiliations:** 1 Department of Thoracic Surgery, The First Affiliated Hospital of Soochow University, Suzhou Medical College of Soochow University; Collaborative Innovation Center of Molecular Medicine between Soochow University and Donghai County People’s Hospital, Clinical Medicine Research Institute of Soochow University and Suzhou BenQ Medical Center, Suzhou Medical College of Soochow University, Suzhou, Jiangsu Province China; 2 Department of Medical Genetics, School of Basic Medical Sciences, Suzhou Medical College of Soochow University, Suzhou, Jiangsu Province China; 3 Donghai County People’s Hospital, Lianyungang, Jiangsu Province China; 4 Department of Thoracic Surgery, Suzhou BenQ Medical Center, Suzhou, Jiangsu Province China; 5 Department of Cell Biology, College of Medicine, Jiaxing University, Jiaxing, Zhejiang Province China; 6 Institute of Minimally Invasive Thoracic Cancer Therapy and Translational Research, Soochow University, Suzhou, Jiangsu Province China; 7 Department of Bioinformatics and Computational Biology, School of Life Sciences, Suzhou Medical College of Soochow University, Suzhou, Jiangsu Province China; 8 Department of Basic Medicine, Kangda College of Nanjing Medical University; Lianyungang Medical-Education Innovation and Research Center, Nanjing Medical University, Lianyungang, Jiangsu Province China; 9 Soochow University Laboratory of Cancer Molecular Genetics, Suzhou Medical College of Soochow University, Suzhou, Jiangsu Province China; 10 Suzhou Key Laboratory for Molecular Cancer Genetics, Suzhou, Jiangsu Province China

**Keywords:** Non-small-cell lung cancer, Mechanisms of disease, Ubiquitylation

## Abstract

The molecular mechanisms underlying non-small cell lung cancer (NSCLC) metastasis remain incompletely understood, limiting the identification of potential therapeutic targets. Here, integrating clinical data, we identify ovarian tumor domain-containing protein 5 (OTUD5), an OTU family member of deubiquitinases, as a potential metastasis suppressor in NSCLC. Reduced OTUD5 expression is observed in metastatic NSCLC specimens and correlates with poor patient survival. Using human NSCLC cell lines, we find that OTUD5 directly interacts with and deubiquitinates transcriptional intermediary factor 1 γ (TIF1γ). The latter attenuates TGF-β-induced SMAD3/4 complex formation, thereby impeding TGF-β-induced repression of *OTUD5* transcription. Upon TGF-β stimulation, OTUD5 overexpression dramatically suppresses SMAD3/SMAD4 complex formation; however, this effect is abrogated when TIF1γ is silenced. OTUD5 overexpression inhibits TGF-β-induced epithelial-mesenchymal transition (EMT) and metastasis of NSCLC cells, whereas these effects are largely abrogated by TIF1γ knockdown. Notably, targeting OTUD5 with nilotinib, an FDA-approved drug for chronic myeloid leukemia (CML), enhances the OTUD5-TIF1γ interaction, reduces the ubiquitination of TIF1γ, and exerts significant anti-metastatic effects on NSCLC cells. Taken together, our findings indicate that OTUD5 inhibits TGF-β-induced EMT and NSCLC cell metastasis in a partially TIF1γ-dependent manner and reveal an OTUD5-TIF1γ-SMAD3/4 positive feedback loop for preventing TGF-β-induced EMT. These findings provide new insights into the molecular basis of NSCLC metastasis and suggest that nilotinib may be repositioned as an anti-metastatic drug by targeting OTUD5 in NSCLC treatment.

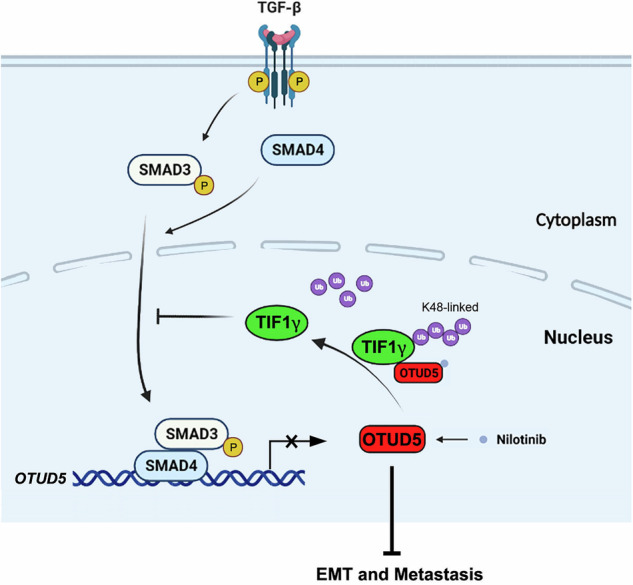

## Introduction

Lung cancer remains the leading cause of cancer-related deaths globally, and non-small cell lung cancer (NSCLC) accounts for approximately 85% of all lung cancers [1]. Deaths from cancer are mostly due to tumor metastasis, including NSCLC [2]. Therefore, it is of great importance to elucidate the precise mechanisms underlying metastasis of NSCLC, as well as to identify potential therapeutic targets.

Epithelial-mesenchymal transition (EMT) is an essential biological process involved in embryogenesis, inflammation, fibrosis, wound healing, and tumor metastasis [3–5]. This transition is characterized by the loss of epithelial markers (e.g., E-cadherin) and the acquisition of mesenchymal markers (e.g., N-cadherin) [6]. Cancer cells undergoing EMT acquire migratory and invasive properties, displaying metastatic potential for colonization of distant organs [7]. EMT associated with metastasis is induced by multiple signaling pathways, including transforming growth factor-β (TGF-β) signaling [8]. We previously reported that activation of TGF-β/SMAD signaling enhances TGF-β-induced EMT in NSCLC cells [9–12]. In particular, we found that transcriptional intermediary factor 1 γ (TIF1γ), a negative regulator of TGF-β/SMAD signaling [13, 14], is downregulated in metastatic NSCLC tissues and inhibits TGF-β-induced EMT and invasion of NSCLC cells [10, 15]. Although we identified a mechanism whereby SOX2-mediated transcriptional inhibition of TIF1γ facilitates TGF-β-induced EMT in NSCLC [10], the post-translational regulation of TIF1γ during NSCLC metastasis remains poorly characterized.

Post-translational modification with ubiquitin is found in all eukaryotic cells and plays a crucial role in various cellular processes [16, 17]. This functional diversity of ubiquitination is achieved by different types of ubiquitin chains, which can be reversed by deubiquitinases (DUBs) [18, 19]. To date, more than 100 DUBs have been identified in human cells, classified into seven families. It is generally accepted that DUB-mediated dysregulation of the ubiquitin system contributes to cancer initiation, progression, and metastasis [19–21]. Among seven families of human DUBs, ovarian tumor proteases (OTUs) constitute the second-largest family and regulate multiple important cellular signaling cascades [18]. For example, the OTU-family deubiquitinases OTUD7B and OTUD5 positively modulate mTOR signaling to promote cancer cell proliferation [22, 23]. In contrast, OTUD5 enhances antiviral and antitumor immunity by positively regulating STING-mediated signaling [24] and OTUD5 suppresses tumorigenesis through deubiquitination of TRIM25 [25]. OTUD5 has been reported to be downregulated in NSCLC [25, 26], suggesting a potential tumor-suppressive role in this malignancy. However, the molecular mechanisms by which OTUD5 regulates TGF-β-induced EMT and metastasis of NSCLC cells, especially at the post-translational level, remain poorly understood.

By integrating clinical data, we identified OTUD5 as a potential metastasis-suppressing OTU-family deubiquitinase in NSCLC. Thus, we initially conducted mass spectrometry (MS)-based proteomic analysis to identify potential substrates and interacting partners of OTUD5 in NSCLC cells. Among the OTUD5-interacting proteins we identified, TIF1γ emerged as a candidate of particular interest. Both TIF1γ and OTUD5 show nuclear localization [10, 27], strengthening the possibility of their interaction. However, to the best of our knowledge, very little is known about the connection between OTUD5 and TIF1γ. Therefore, we investigated whether OTUD5 interacts with TIF1γ to regulate TGF-β-induced EMT and metastasis in NSCLC cells.

In this study, we demonstrate that OTUD5 is transcriptionally repressed by TGF-β-activated SMAD3/4 complex. Like TIF1γ [10, 15], OTUD5 also inhibits TGF-β-induced EMT and metastasis of NSCLC cells. Importantly, we find that OTUD5 directly interacts with and deubiquitinates TIF1γ, promoting its stability. This attenuates TGF-β-induced formation of SMAD3/4 complex, which in turn undermines TGF-β-mediated transcriptional repression of OTUD5. In vivo experiments show that OTUD5 represses TGF-β-induced EMT and NSCLC cell metastasis in a partially TIF1γ-dependent manner. This study reveals that the OTUD5-TIF1γ-SMAD3/4 positive feedback loop is essential for preventing TGF-β-induced EMT and metastasis of NSCLC cells, thereby extending our understanding of the interplay between OTUD5 and TGF-β/SMAD signaling. Through structure-based virtual screening, we identify nilotinib, an FDA-approved tyrosine kinase inhibitor (TKI) drug for chronic myeloid leukemia (CML) [28, 29], as a compound that exerts anti-metastatic effects by directly binding OTUD5 in NSCLC.

## Results

### Identification of tumor metastasis-associated OTUs in human NSCLC

**Fig. 1:**
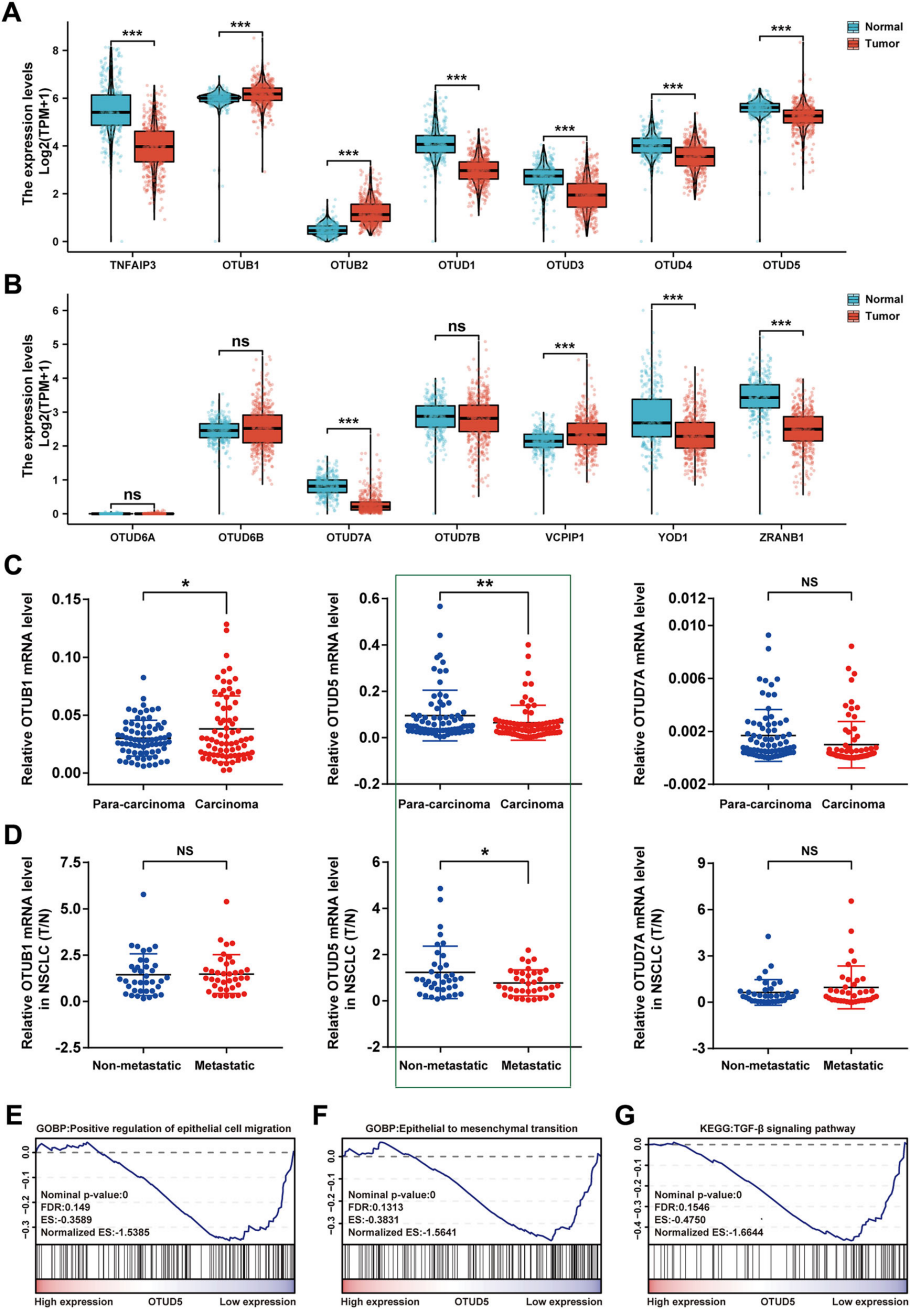
OTUD5 expression is reduced in metastatic NSCLC tissues and negatively associated with EMT and TGF-β signaling. **A**, **B** The relative mRNA levels of 14 OTUs, including TNFAIP3, OTUB1/2, OTUD1/3/4/5, OTUD6A/6B, OTUD7A/7B, VCPIP1, YOD1, and ZRANB1, in normal and tumor tissues of NSCLCs (*n* = 862) from TCGA and GTEx databases. Data are shown as the mean ± SD. ns, not significant; ***P < 0.001 by unpaired Student’s *t* test. **C** RT-qPCR analysis of three OTUs (OTUB1, OTUD5, and OTUD7A) mRNA levels in 74 NSCLC tissues (*n* = 74) and their matched para-carcinoma tissues. ns, not significant; *P < 0.05 and **P < 0.01 by unpaired Student’s *t* test. The experiment was repeated three times for confirmation (biological replicates). **D** The relative mRNA expression (T/N) of OTUB1, OTUD5, and OTUD7A in non-metastatic (*n* = 37) and metastatic (*n* = 37) NSCLC tissues. NSCLC tissues were categorized into non-metastatic and metastatic tissues as described in the Materials and Methods. T, NSCLC tumor tissues; N, para-carcinoma tissues. ns, not significant; *P < 0.05 by unpaired Student’s *t* test. The experiment was repeated three times for confirmation (biological replicates). **E**–**G** GSEA was performed based on GSE19804 from Gene Expression Omnibus (GEO) database. Three gene sets, including epithelial cell migration, EMT and TGF-β signaling pathway, were enriched in NSCLC specimens with OTUD5 low-expression. FDR, false discovery rate. ES, enrichment score. GSEA was considered to be significant at predefined values of *p* < 0.05, FDR < 0.25, and |Normalized ES | > 1.

To identify OTU-family deubiquitinases associated with NSCLC metastasis, we screened 14 human OTUs based on their expression patterns (Fig. 1A, B) and correlations with overall survival (Figures S1A–S1N) in NSCLC samples from TCGA and GTEx databases. OTUB1, OTUD5, and OTUD7A emerged as candidates that were differentially expressed between normal and NSCLC tumor tissues and significantly correlated with patient survival. Then, we examined the mRNA expression levels of these three genes in 74 NSCLC tissues and paired para-carcinoma tissues. OTUB1 was significantly upregulated, whereas OTUD5 was significantly downregulated in NSCLC tissues; OTUD7A expression remained unchanged (Fig. 1C and Table S1). Furthermore, among the three candidates, only OTUD5 expression was significantly lower in metastatic NSCLC tissues (*n* = 37) compared with non-metastatic (*n* = 37) counterparts (Fig. 1D and Table S1). Additionally, NSCLC patients with low expression of OTUD5 exhibited poor survival (Figure S1G). Sex-stratified analysis of OTUD5 expression revealed no statistically significant differences between male and female patients (Figure S1O). Using dataset GSE19804 from the Gene Expression Omnibus (GEO) database, we carried out gene set enrichment analysis (GSEA) and found that gene sets related to epithelial cell migration, EMT, and TGF-β signaling pathway were substantially enriched in NSCLC specimens with low OTUD5 expression (Fig. 1E–G). These results suggest that OTUD5 expression is inversely correlated with tumor metastasis, EMT, and TGF-β signaling in NSCLC.

### TGF-β-induced SMAD3/4 complex transcriptionally inhibits OTUD5 expression in NSCLC cells

**Fig. 2:**
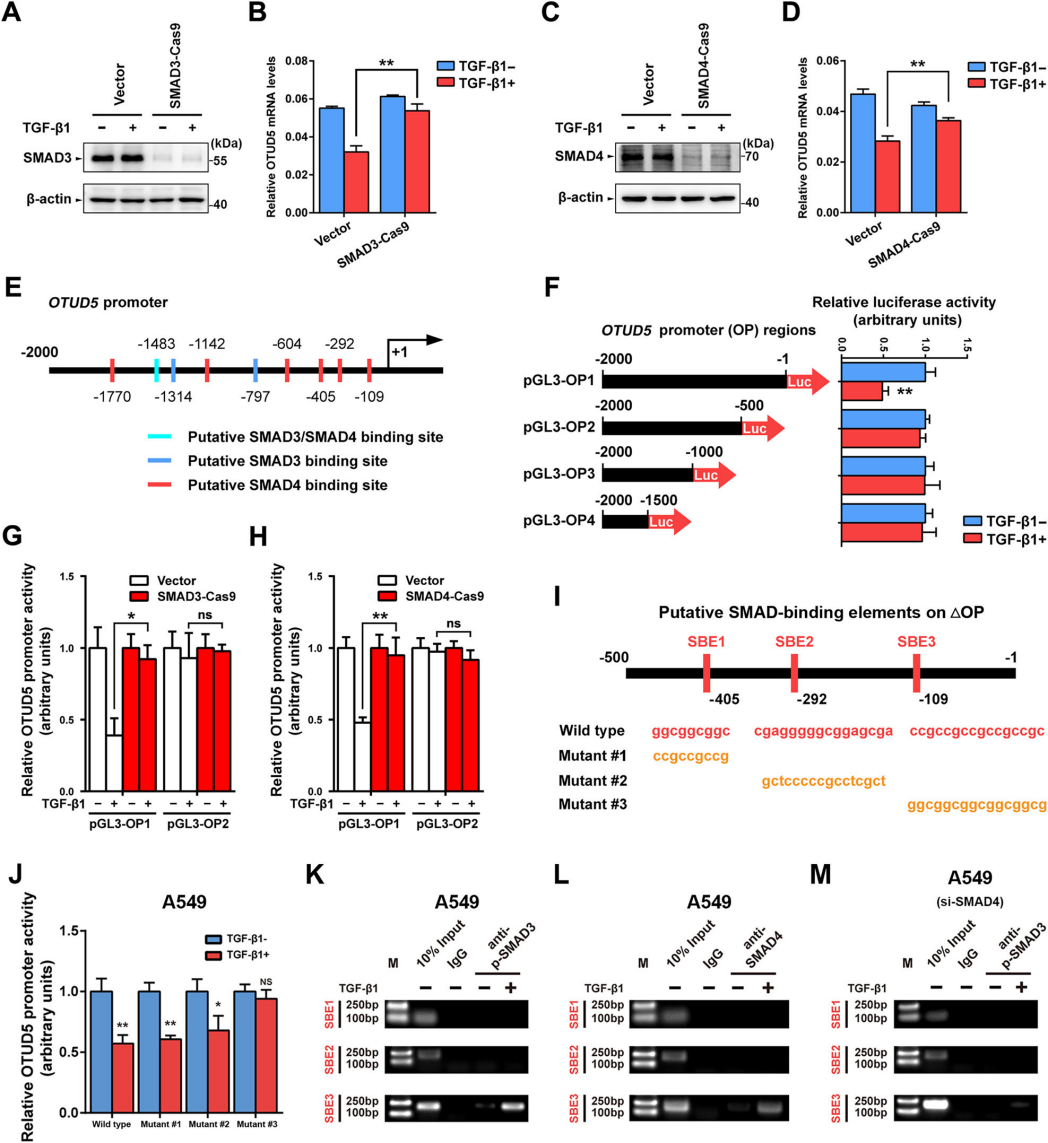
OTUD5 is transcriptionally inhibited by TGF-β-induced SMAD3/4 complex in NSCLC cells. **A**–**D** SMAD3- and SMAD4-silenced A549 cells with CRISPR/Cas9 system (SMAD3- and SMAD4-Cas9) and A549 control cells were serum starved and then treated with or without TGF-β1 (5 ng/mL) for 24 h. The expression of SMAD3, SMAD4 protein (**A**, **C**) and OTUD5 mRNA (**B**, **D**) in the above cells were determined by western blot and RT-qPCR, respectively. Data are shown as the mean ± SD of *n* = 3 technical replicates, **P < 0.01 by unpaired Student’s *t*-test. The experiment was repeated three times for confirmation (biological replicates). **E** In silico analysis predicted the *OTUD5* promoter region (position −2000 to +1) harbors several putative SBEs, containing SMAD3/SMAD4-binding sites (cyan box), SMAD3-binding sites (blue box). and SMAD4-binding sites (red box). The position of the first base of each SBE consensus DNA sequence is indicated. **F** A series of pGL3 luciferase reporter constructs (pGL3-OP1, 2, 3, and 4) containing different fragments of the *OTUD5* promoter were prepared (left panel) and transiently transfected into A549 cells. After being serum starved for 24 h, A549 cells were treated with or without TGF-β1 (5 ng/mL) for 24 h. Their luciferase activities (right panel) were determined as described in Materials and Methods. OP, *OTUD5* promoter. Data are shown as the mean ± SD of *n* = 3 technical replicates, **P < 0.01 by unpaired Student’s *t* test. The experiment was repeated three times for confirmation (biological replicates). **G**, **H** The pGL3-OP1 and pGL3**-**OP2 constructs were transfected into A549 cells with SMAD3-Cas9 (**G**), SMAD4-Cas9 (**H**) and empty vectors. Then, the cells were treated as above and subjected to luciferase reporter assays for *OTUD5* promoter activity. Data are shown as the mean ± SD of *n* = 3 technical replicates. ns, not significant; *P < 0.05 and **P < 0.01 by unpaired Student’s *t*-test. The experiment was repeated three times for confirmation (biological replicates). **I** Schematic diagram shows the position of three putative SBEs (SBE1, 2 and 3) in the ΔOP region (position -500 to -1). The wild type and mutant of each SBE consensus sequence are indicated below. OP: OTUD5 promoter; SBE, SMAD-binding element. **J** Four different pGL3-ΔOP luciferase reporter constructs encompassing the wild-type or mutants of three SBEs were transfected into A549 cells, and the cells were treated as above, followed by the detection of *OTUD5* promoter activity. Data are shown as the mean ± SD of *n* = 3 technical replicates. ns, not significant; *P < 0.05 and **P < 0.01 by unpaired Student’s *t* test. The experiment was repeated three times for confirmation (biological replicates). **K**, **L** TGF-β1-treated or -untreated A549 cells were subjected to ChIP analysis, in which anti-p-SMAD3 (**K**) or anti-SMAD4 (**L**) antibodies were used. Before immunoprecipitation, 10% input was obtained as a positive control and allowed to PCR to confirm the DNA fragments containing SBEs in the ΔOP. Anti-IgG antibody served as a negative control. The immunoprecipitated DNA was subjected to PCR and gel staining analysis for detecting the enrichment of SBEs. SBE, SMAD-binding element. **M** A549 cells with SMAD4 knockdown were treated as above, followed by ChIP analysis using anti-p-SMAD3 antibodies. SBE, SMAD-binding element.

Based on the aforementioned GSEA results and our previous findings that TGF-β effectively induces EMT in NSCLC cells [9–12, 15], we deduced that TGF-β may negatively affect OTUD5 expression in NSCLC cells. Thus, we detected the expression changes of OTUD5 in TGF-β1-treated and -untreated A549 and H1650 cells. As expected, TGF-β1 treatment significantly decreased both mRNA and protein levels of OTUD5 in NSCLC cells (Figures S2A and S2B). Subsequently, we investigated the molecular mechanism by which TGF-β inhibits OTUD5 expression. Given that SMAD3/SMAD4 function as both essential mediators of TGF-β/SMAD signaling and transcriptional regulators [30], we generated SMAD3- or SMAD4-silenced A549 cells using CRISPR/Cas9 system. Upon TGF-β1 stimulation, SMAD3 or SMAD4 knockout partly abrogated TGF-β1-mediated inhibition of OTUD5 mRNA expression (Fig. 2A–D), suggesting that TGF-β1 transcriptionally represses OTUD5 expression by relying on the SMAD3/4 complex. Using hTFtarget database (https://guolab.wchscu.cn/hTFtarget), we identified several putative SMAD-binding elements (SBEs) within the ~2000-bp 5′-region of the *OTUD5* promoter (Fig. 2E). According to the prediction, we generated a series of pGL3 luciferase reporter constructs containing various *OTUD5* promoter fragments (Fig. 2F, left panel) and transfected them into A549 cells with or without TGF-β1 treatment. Upon TGF-β1 treatment, the luciferase activity of pGL3-OP1 (position -2000 to -1) was significantly decreased compared with that of pGL3-OP2/3/4, from which the fragment (termed ΔOP, position -500 to -1) had been deleted (Fig. 2F, right panel). These results indicate that ΔOP region harbors SMAD3/4-binding elements that mediate TGF-β-mediated transcriptional repression of the *OTUD5* gene.

Furthermore, SMAD3 or SMAD4 knockout augmented the pGL3-OP1 activity in the presence of TGF-β1, whereas neither affected pGL3-OP2 activity regardless of TGF-β1 treatment (Fig. 2G, H), indicating that SMAD3 and SMAD4 negatively regulate TGF-β1-mediated *OTUD5* promoter activity through binding to the ΔOP region. To identify the specific functional SBE within the ΔOP region, we created pGL3-ΔOP luciferase reporter plasmids containing wild-type or mutants of three SBEs (Fig. 2I). Results of luciferase assay showed that TGF-β1-mediated repression of pGL3-ΔOP activity was abolished only when SBE3 was mutated (Fig. 2J), suggesting that SBE3 (position -109 to -124) in the *OTUD5* promoter serves as the functional SMAD3/4 complex-binding site. To further validate the above results, we performed chromatin immunoprecipitation (ChIP) assays using phosphorylated SMAD3 (p-SMAD3) and SMAD4 antibodies. ChIP assays revealed that both p-SMAD3 and SMAD4 bound to the SBE3-containing promoter fragment, with enrichment significantly increased upon TGF-β1 treatment (Fig. 2K, L). Notably, p-SMAD3 binding to SBE3 was dramatically decreased when SMAD4 was knocked down (Fig. 2M). Collectively, these results demonstrate that OTUD5 expression is transcriptionally inhibited by TGF-β-induced SMAD3/4 complex in NSCLC cells.

### OTUD5 inhibits TGF-β-induced EMT and invasion of NSCLC cells

**Fig. 3:**
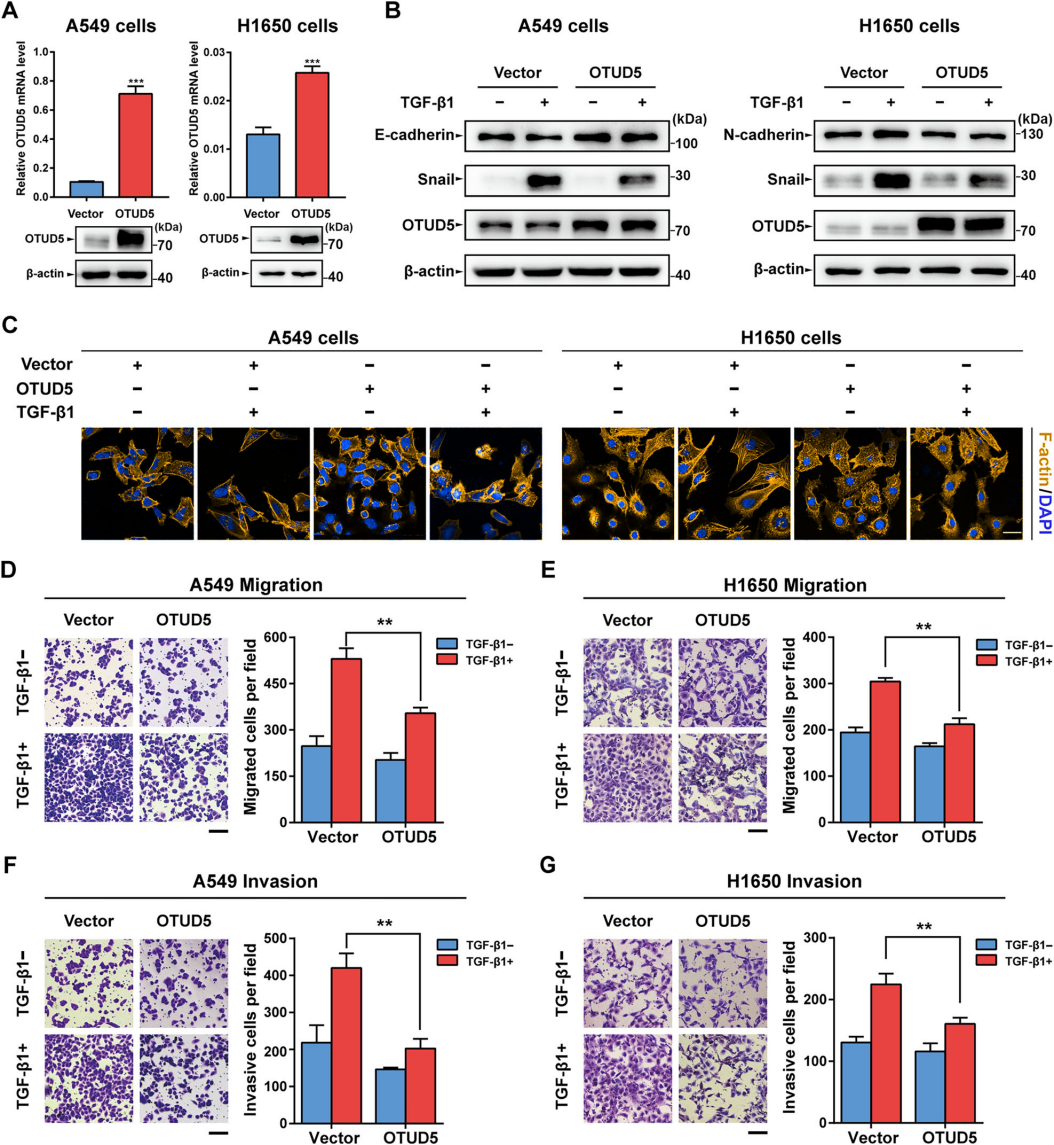
OTUD5 overexpression suppresses TGF-β-induced EMT and invasion of NSCLC cells. **A** RT-qPCR and western blot analyses of OTUD5 expression in OTUD5-overexpressing A549 and H1650 cells. β-actin served as a loading control. Data are shown as the mean ± SD of *n* = 3 technical replicates, ***P < 0.001 by unpaired Student’s *t*-test. The experiment was repeated three times for confirmation (biological replicates). **B** After being serum starved, OTUD5-overexpressing A549 and H1650 cells were treated with or without TGF-β1 (5 ng/mL) for 24 h. Then, western blot analysis was performed to examine the expression of the indicated EMT markers. **C** OTUD5-overexpressing A549 and H1650 cells and control cells were treated as above, and F-actin (orange) was stained with iFluor 555-labeled phalloidin. Cell nuclei were counterstained and visualized with DAPI (blue). Scale bar, 100 μm. **D**–**G** OTUD5-overexpressing A549 and H1650 cells were treated as above and allowed to migrate through an 8-μm pore membrane (**D**, **E**) or invade through a Matrigel-coated membrane (**F**, **G**) in Transwells. After 24 h, the migratory and invasive cells were stained, photographed, and counted in at least three light microscopic fields per well. Representative images and the migratory and invasive cell numbers were shown. Scale bar, 100 μm. Data are shown as the mean ± SD of *n* = 3 technical replicates, **P < 0.01 by unpaired Student’s *t* test. The experimen*t* was repeated three times for confirmation (biological replicates).

Considering the above findings and our previous studies showing that TGF-β/SMAD signaling activation enhances EMT and invasion of NSCLC cells [9, 11, 12], we examined whether OTUD5 inhibits TGF-β-induced EMT and invasion of NSCLC cells. OTUD5 overexpression significantly impaired TGF-β-induced downregulation of the epithelial marker E-cadherin and upregulation of the mesenchymal markers N-cadherin and Snail in A549 and H1650 cells (Fig. 3A, B). But short hairpin RNA (shRNA)-mediated silencing of OTUD5 had the opposite effect (Figure S3A and S3B). Moreover, we stained the cytoskeleton using phalloidin and examined cell morphology. After treatment with TGF-β1, control A549 and H1650 cells exhibited a mesenchymal spindle-shaped morphology, whereas OTUD5 overexpression largely reversed this morphological transition (Fig. 3C). OTUD5 depletion promoted TGF-β-induced morphological changes toward a mesenchymal shape (Figure S3C). Furthermore, Transwell migration and invasion assays indicated that OTUD5 overexpression effectively alleviated TGF-β-induced migration and invasion of A549 and H1650 cells (Fig. 3D–G). By contrast, OTUD5 depletion facilitated TGF-β-induced migratory and invasive capabilities of A549 and H1650 cells (Figures S3D–S3G). Taken together, these results show that OTUD5 suppresses TGF-β-induced EMT and invasion of NSCLC cells.

### OTUD5 interacts with TIF1γ

**Fig. 4:**
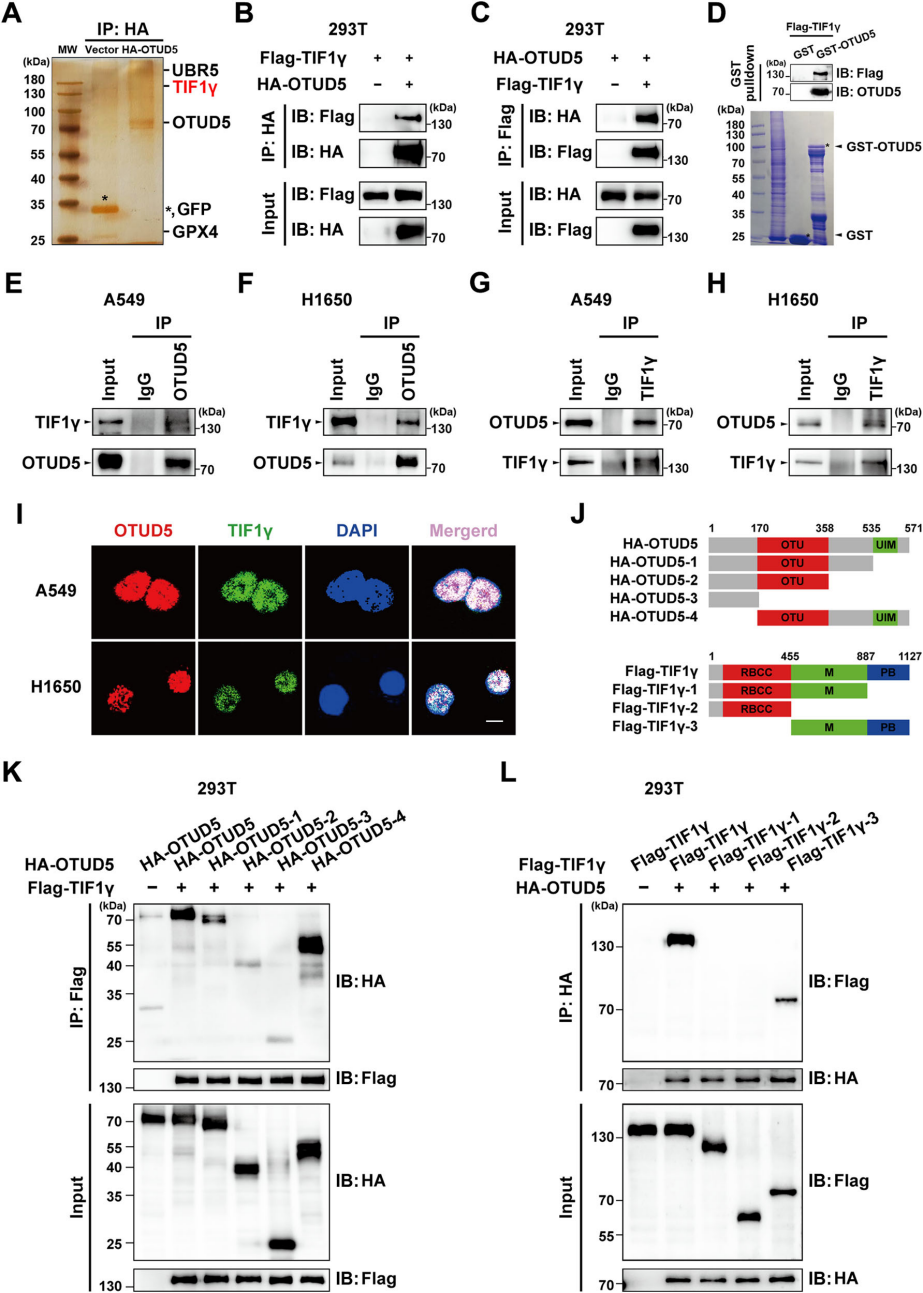
OTUD5 interacts with TIF1γ. **A** HEK 293 T cells were transfected with HA-tagged OTUD5 expression or control vectors for 48 h, and cell lysates were immunoprecipitated with anti-HA magnetic beads. The OTUD5-binding proteins were eluted and subjected to MS-based proteomic analysis. Eluted proteins were separated on SDS-PAGE gel and visualized by silver staining. IP, immunoprecipitation. **B**, **C** Cell lysates of HEK 293 T cells co-expressing HA-OTUD5 and/or Flag-TIF1γ were subjected to co-IP using anti-HA (**B**) or anti-Flag antibodies (**C**) and immunoblotted with anti-Flag or anti-HA antibodies. IB, immunoblot. **D** Recombinant Glutathione S-transferase (GST)-OTUD5 pulls down Flag-TIF1γ in a cell-free system as described in Materials and Methods. IB analysis was performed using anti-Flag or anti-OTUD5 antibodies (upper panel), and Coomassie Blue staining shows purified GST-OTUD5 or GST proteins (lower panel). **E**, **F** Co-IP was performed in A549 and H1650 cells. Endogenous OTUD5 was immunoprecipitated with anti-OTUD5 antibody. The binding of TIF1γ to OTUD5 were determined by IB with anti-TIF1γ antibody. An anti-IgG antibody served as a negative control. **G**, **H** Co-IP was conducted using anti-TIF1γ antibody in the lysates of A549 and H1650 cells. The indicated proteins were determined by IB. **I** A549 or H1650 cells were co-incubated with a rabbit anti-OTUD5 antibody and a mouse anti-TIF1γ antibody. Then, cells were stained with Cy3-conjugated anti-rabbit IgG (red, for OTUD5) and FITC-conjugated anti-mouse IgG (green, for TIF1γ). Cell nuclei were counterstained and visualized with DAPI (blue). Scale bar, 10 μm. **J** Schematic diagrams show a full-length (FL) and four truncated coding sequences (CDSs) of OTUD5 (top panel) and an FL and three truncated CDSs of TIF1γ (bottom panel), which were used for construction of HA-tagged OTUD5 or Flag-tagged TIF1γ expression vectors. OTU, ovarian tumor domain; UIM, ubiquitin-interacting motif domain; RBCC, RING, B-box, coiled-coil motif; M, middle linker; PB, a PHD domain and a bromodomain. **K** The five above-mentioned HA-tagged OTUD5 vectors and Flag-TIF1γ were co-transfected into HEK 293 T cells as indicated, and then cell lysates were subjected to co-IP assay using anti-Flag antibody and immunoblotted with indicated antibodies, which are for detection of domains of OTUD5 interacting with TIF1γ. **L** Lysates of HEK 293 T cells co-transfected with the four above-mentioned Flag-tagged TIF1γ vectors and HA-OTUD5 were subjected to co-IP analysis using anti-HA antibody and immunoblotted with indicated antibodies, which are for the detection of domains of TIF1γ interacting with OTUD5.

To identify undiscovered OTUD5-interacting proteins, we performed an anti-hemagglutinin (HA) immunoprecipitation (IP) assay followed by MS-based proteomic analysis in HEK 293 T cells transfected with HA-tagged OTUD5. A total of 492 candidate OTUD5-interacting proteins were identified (Fig. 4A and Table S2). In support of our results, UBR5 and GPX4, previously reported OTUD5 interactors [27, 31], were detected in our MS data (Table S2). Among these OTUD5-interacting proteins, TIF1γ attracted particular attention and was visualized by silver staining (Fig. 4A). This is because of our findings that both OTUD5 (Fig. 3B–G) and TIF1γ inhibit TGF-β-induced EMT and invasion of NSCLC cells [10, 15].

Furthermore, we validated the interaction of OTUD5 with TIF1γ. In HEK 293 T cells co-transfected with HA-OTUD5 and/or Flag-TIF1γ expression vectors, TIF1γ and OTUD5 were co-immunoprecipitated with OTUD5 and TIF1γ, respectively (Fig. 4B, C). In a cell-free system, recombinant GST-OTUD5 pulled down Flag-TIF1γ in vitro (Fig. 4D), confirming a direct interaction between OTUD5 and TIF1γ. Endogenous TIF1γ was coprecipitated with OTUD5 in A549 and H1650 cells (Fig. 4E, F) and vice versa (Fig. 4G, H). Moreover, immunofluorescence staining showed that OTUD5 and TIF1γ co-localize in the nucleoplasm (Fig. 4I), further supporting their interaction in NSCLC cells. Next, we tried to map the interaction domains mediating OTUD5-TIF1γ association. To this end, we generated HA-tagged OTUD5 constructs comprising full-length (FL) and four truncated coding sequences (CDSs), as well as Flag-tagged TIF1γ constructs comprising FL and three truncated CDSs (Fig. 4J). These constructs were co-transfected into HEK 293 T cells for domain mapping experiments. Co-IP analyses showed that the amount of OTUD5 coprecipitated with TIF1γ was remarkably decreased when the ubiquitin-interacting motif (UIM)-containing domain was deleted from OTUD5 (Fig. 4K), suggesting that the UIM domain is critical for the OTUD5-TIF1γ interaction. Besides, the PB domain of TIF1γ was required for binding to OTUD5 (Fig. 4L). Overall, these findings demonstrate that OTUD5 directly interacts with the PB domain of TIF1γ by effectively utilizing UIM domain.

### OTUD5 stabilizes TIF1γ via deubiquitination

**Fig. 5:**
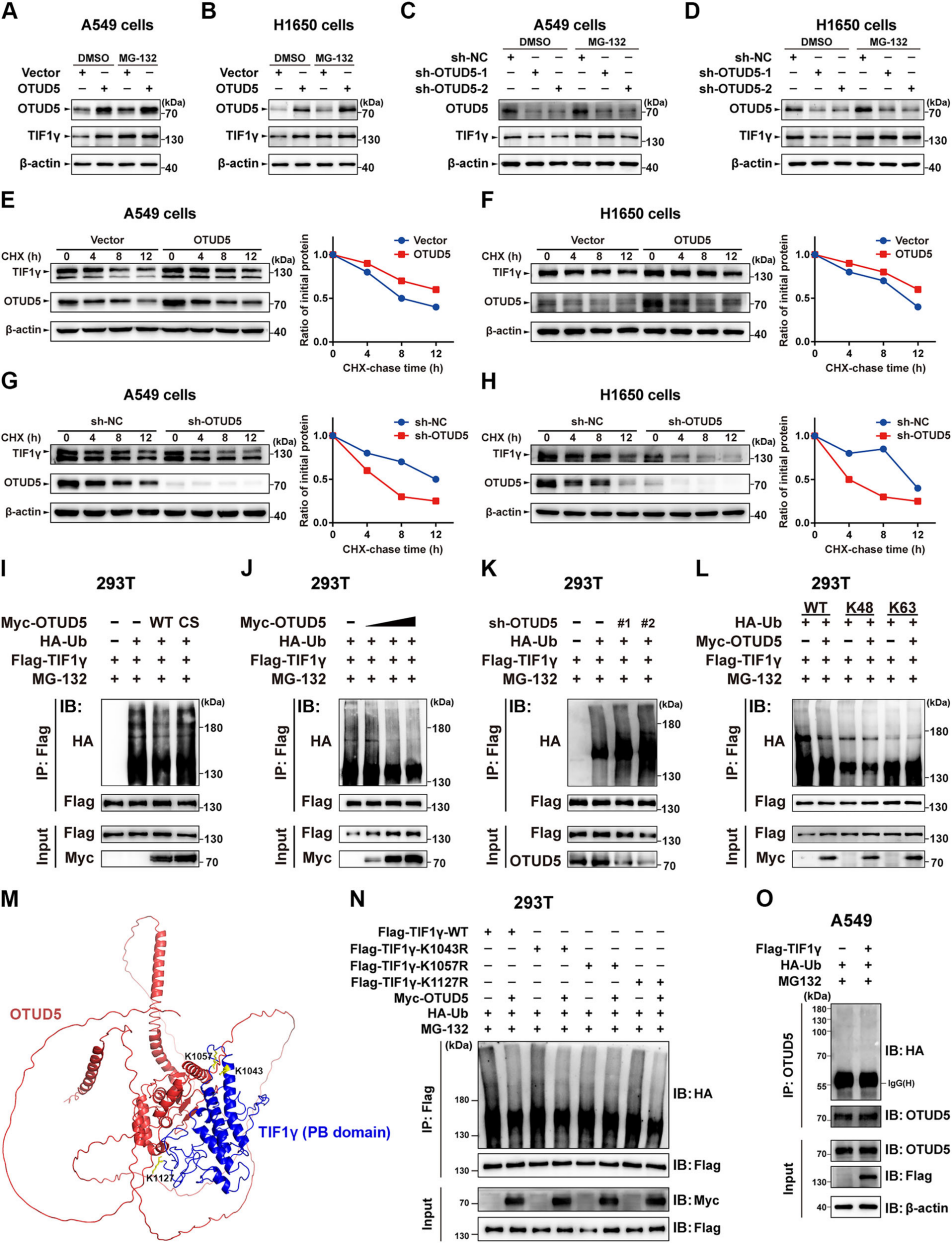
OTUD5 stabilizes TIF1γ via deubiquitination. **A**–**D** OTUD5-overexpressing/silenced A549 and H1650 cells were treated with MG-132 (10 μM) for 6 h and harvested for western blot analysis of TIF1γ and OTUD5 expression. β-actin served as an internal control. sh-OTUD5, short hairpin RNA-mediated silencing of OTUD5. MG-132, proteasome inhibitor. **E**–**H** OTUD5-overexpressing/silenced A549 and H1650 cells were treated with CHX (150 μg/mL) for the indicated times, and then immunoblotted with antibodies against TIF1γ, OTUD5 and β-actin as loading control. CHX, protein biosynthesis inhibitor cycloheximide. **I** Flag-TIF1γ-overexpressing HEK 293 T cells were co-transfected with HA-Ub and/or Myc-OTUD5/CS as indicated for 42 h, and then treated with MG-132 (10 μM) for 6 h before being harvested. Cell lysates were immunoprecipitated with anti-Flag antibodies and immunoblotted with anti-HA antibodies. Ub, ubiquitin. WT, wild-type. CS, C224S (OTUD5-inactivated mutant). **J**, **K** Flag-TIF1γ-overexpressing HEK 293 T cells were co-transfected with HA-Ub and/or an increasing dose of Myc-OTUD5 (J) or sh-OTUD5 (K) as indicated, cell lysates were immunoprecipitated with anti-Flag antibodies and then immunoblotted with anti-HA antibodies. Cells were treated with MG-132 (10 μM) for 6 h before being harvested. **L** Flag-TIF1γ and HA-Ub or Ub-K48/K63 mutants were co-transfected into HEK 293 T cells with or without Myc-OTUD5 expression for 42 h. Then, cells were treated with MG-132 (10 μM) for 6 h, followed by anti-Flag IP and IB with indicated antibodies. Ub-K48 or -K63 represents that all 7 lysines of Ub were mutated to arginines except K48 or K63. K, lysine. **M** AlphaFold prediction of OTUD5 (red) and PB domain of TIF1γ (blue) interaction. Yellow sticks represent the lysine residues K1043, K1057 and K1127 of TIF1γ, which are located on the surface of OTUD5-TIF1γ (PB domain) interaction. **N** Flag-TIF1γ-WT, Flag-K1043R, Flag-TIF1γ-K1057R, Flag-TIF1γ-K1127R and HA-Ub were respectively transfected into HEK 293 T cells with or without Myc-OTUD5 as indicated. Cells were treated as above with MG-132, followed by anti-Flag IP and IB with indicated antibodies. R, arginine. **O** A549 cells were co-transfected with Flag-TIF1γ and HA-Ub for 48 h, and cell lysates were immunoprecipitated with anti-OTUD5 antibodies. IB with anti-HA antibodies for monitoring the ubiquitylation status of OTUD5.

Given the facts that OTUD5 functions as a deubiquitinase [18] and directly interacts with TIF1γ (Fig. 4A–L), we examined whether OTUD5 regulates TIF1γ protein levels in NSCLC cells. Overexpression and knockdown of OTUD5 respectively increased and decreased TIF1γ protein levels in A549 and H1650 cells, which were abrogated by MG-132, a proteasome inhibitor (Fig. 5A–D). Notably, neither overexpression nor knockdown of OTUD5 altered TIF1γ mRNA levels (Figures S4A–S4D). These results suggest that OTUD5 regulates TIF1γ at post-translational level. To directly assess protein stability, we performed cycloheximide (CHX, a protein biosynthesis inhibitor) chase assays. OTUD5 overexpression prolonged the half-life of TIF1γ in CHX-treated A549 and H1650 cells (Figs. 5E, F), whereas OTUD5 knockdown shortened it (Fig. 5G, H). These findings suggested that OTUD5 stabilizes TIF1γ through deubiquitination. To test this hypothesis, co-IP experiments demonstrated that TIF1γ ubiquitination was inhibited by wild-type OTUD5 rather than deubiquitination-deficient mutant of OTUD5 (C224S) [32, 33] in HEK 293 T cells (Fig. 5I), indicating that OTUD5 acts as a deubiquitinase to repress TIF1γ ubiquitination. TIF1γ ubiquitination decreased in a dose-dependent manner upon OTUD5 overexpression (Fig. 5J) and increased upon OTUD5 knockdown (Fig. 5K). In fact, OTUD5 was reported to cleave K48-linked polyubiquitin chains on STING or K63-linked polyubiquitin chains on TRAF3 [24, 33]. To determine the ubiquitin chain linkage specificity, we performed anti-Flag co-IP in HEK 293 T cells transfected with Myc-OTUD5/empty, Flag-TIF1γ, and HA-tagged wild-type or mutant ubiquitin (all 7 lysines mutated to arginines except K48 or K63) and found that OTUD5 diminished K48-linked polyubiquitination of TIF1γ (Fig. 5L). Furthermore, an AlphaFold-predicted structural modeling [34] suggests a dimeric OTUD5-TIF1γ (PB domain) complex, in which three lysine residues (K1043, K1057 and K1127) of TIF1γ are located on the surface of OTUD5 and TIF1γ (PB domain) interaction (Fig. 5M). In light of this prediction, we examined which lysine residues could play a role in OTUD5-mediated deubiquitination of TIF1γ. HEK 293 T cells were transfected with Flag-TIF1γ or three TIF1γ mutants (K1043R, K1057R and K1127R), together with Myc-OTUD5/empty and HA-ubiquitin, and subjected to co-IP assays. The results showed that only OTUD5-mediated TIF1γ (K1057R) deubiquitination was almost abolished (Fig. 5N), suggesting that OTUD5 deubiquitinates TIF1γ at K1057 residue. Due to the E3 ubiquitin ligase function of TIF1γ [35, 36], we determined whether TIF1γ reciprocally regulates OTUD5 ubiquitination. As illustrated in Fig. 5O, endogenous OTUD5 ubiquitination was unaffected by TIF1γ overexpression in A549 cells. Taken together, these results demonstrate that OTUD5 stabilizes TIF1γ by removing K48-linked polyubiquitin chains at K1057, thereby preventing its proteasomal degradation.

### OTUD5 inhibits TGF-β-induced SMAD3/4 complex formation via TIF1γ, which in turn impedes TGF-β-induced repression of OTUD5 transcription

**Fig. 6:**
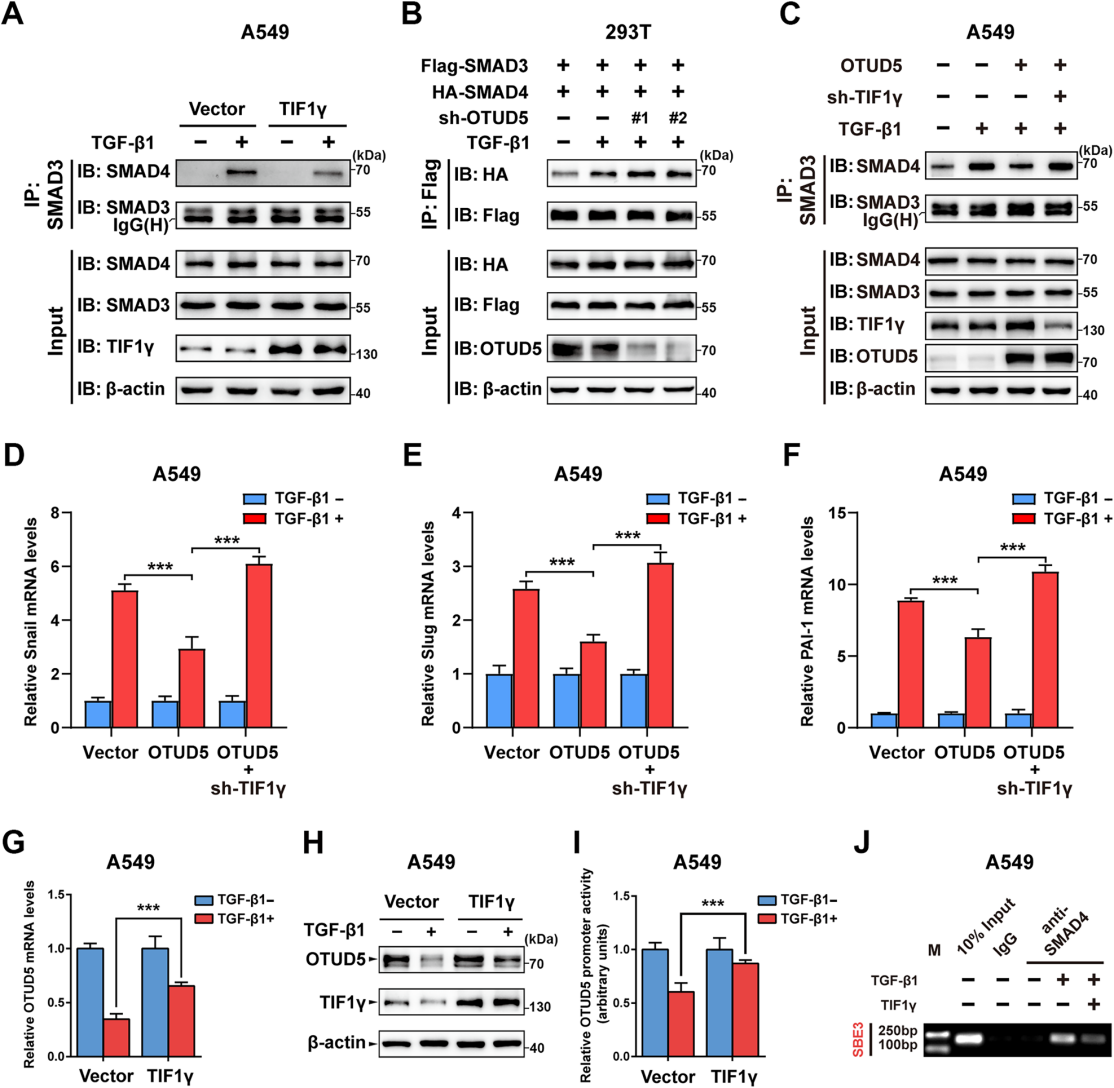
OTUD5 blocking TGF-β-induced SMAD3/4 complex formation depends on TIF1γ, which in turn impedes TGF-β-induced inhibition of OTUD5 transcription. **A** TIF1γ-overexpressing A549 cells were treated with or without TGF-β1 (5 ng/mL) for 1 h, and subjected to co-IP using SMAD3 antibodies. The amount of SMAD4 bound to SMAD3 was detected by IB using anti-SMAD4 antibodies. **B** HEK 293 T cells were transfected with Flag-SMAD3, HA-SMAD4, and/or sh-OTUD5 as indicated, then treated with TGF-β1 (5 ng/mL) for 1 h. Cell lysates were subsequently immunoprecipitated with anti-Flag antibodies. The amount of SMAD4 bound to SMAD3 was determined by IB with anti-HA antibodies. **C** OTUD5-overexpressing A549 cells were transfected with sh-TIF1γ and treated as above with TGF-β1, cell lysates were immunoprecipitated with anti-SMAD3 antibodies, then immunoblotted with anti-SMAD4 antibodies. **D**–**F** RT-qPCR analysis of Snail, Slug and PAI-1 mRNA expression in vector control, OTUD5-overexpressing and OTUD5-overexpressing/sh-TIF1γ A549 cells treated with TGF-β1 (5 ng/mL) for 24 h. Data are shown as the mean ± SD of *n* = 3 technical replicates, ***P < 0.001 by unpaired Student’s *t* test. The experiment was repeated three times for confirmation (biological replicates). **G**, **H** RT-qPCR and western blot analyses of OTUD5 mRNA expression in TIF1γ-overexpressing A549 cells with TGF-β1 (5 ng/mL) treatment for 24 h. Data are shown as the mean ± SD of *n* = 3 technical replicates, ***P < 0.001 by unpaired Student’s *t*-test. The experiment was repeated three times for confirmation (biological replicates). **I** The pGL3-ΔOP (position -500 to -1) luciferase reporter construct (Fig. 2I) was transfected into TIF1γ-overexpressing A549 cells. Cells were then treated with TGF-β1 (5 ng/mL) for 24 h and subjected to luciferase reporter assays for OTUD5 promoter activity. Data are shown as the mean ± SD of *n* = 3 technical replicates, ***P < 0.001 by unpaired Student’s *t* test. The experiment was repea*t*ed three times for confirmation (biological replicates). **J** TGF-β1-treated or -untreated TIF1γ-overexpressing A549 cells were subjected to ChIP analysis, in which anti-SMAD4 antibodies were used. Before IP, 10% input was used as a positive control and allowed to PCR to confirm the DNA fragments containing SBE3 in ΔOP of *OTUD5* promoter. Anti-IgG antibody served as a negative control. The immunoprecipitated DNA was subjected to PCR and gel staining analysis to determine the enrichment of SBE3. SBE, SMAD-binding element.

Previous studies, including our own, have demonstrated that TIF1γ serves as a negative regulator of TGF-β/SMAD signaling by disrupting formation of SMAD3/SMAD4 transcriptional complex [10, 13, 14, 37, 38]. Consistent with these reports, our co-IP experiments confirmed that TIF1γ overexpression weakened the binding ability of SMAD4 to SMAD3 in TGF-β1-treated A549 cells (Fig. 6A). Based on our above findings that OTUD5 interacts with and stabilizes TIF1γ (Figs. 4 and 5A–N), we assumed that OTUD5 inhibits TGF-β-induced SMAD3/4 complex formation. As hypothesized, upon TGF-β1 stimulation, OTUD5 knockdown significantly increased the amount of SMAD4 bound to SMAD3 (Fig. 6B) and OTUD5 overexpression decreased the formation of SMAD3/4 complex (Fig. 6C). However, the repression of SMAD3/SMAD4 complex formation by OTUD5 overexpression was relieved when TIF1γ was silenced (Fig. 6C), suggesting that the regulation of TGF-β-induced SMAD3/4 complex formation by OTUD5 depends on TIF1γ. In support of this, OTUD5 overexpression or knockdown failed to change the expression of the key TGF-β/SMAD signaling components, including TGFβR1/2, SMAD3/4, and SMAD6/7, in A549 and H1650 cells (Figures S5A–S5D). Moreover, in TGF-β1-treated A549 cells, OTUD5 overexpression dramatically inhibited mRNA expression of TGF-β/SMAD target genes, including Snail, Slug, and PAI-1; however, this effect was reversed by TIF1γ knockdown (Fig. 6DD–F). These results indicate that OTUD5 inhibits TGF-β/SMAD signaling by relying on TIF1γ.

Logically, the aforementioned results (Figs. 2A–M, S2A, S2B, and 6A–F) led us to hypothesize that TIF1γ could destroy the inhibitory effect of TGF-β/SMAD signaling on *OTUD5* transcription. To verify this hypothesis, we firstly examined OTUD5 expression in TIF1γ-overexpressing A549 cells treated with or without TGF-β1, and found that TIF1γ overexpression impaired TGF-β-mediated inhibition of OTUD5 mRNA and protein expression (Fig. 6G, H). Luciferase reporter assay showed that TIF1γ overexpression significantly inverted TGF-β-mediated inhibition of *OTUD5* promoter activity in A549 cells (Fig. 6I). Consistent with this, ChIP analysis revealed that TIF1γ overexpression reduced SMAD4 binding to SBE3 in the *OTUD5* promoter (Fig. 6J). Taken together, these results reveal that TIF1γ impedes TGF-β-mediated transcriptional repression of OTUD5 by disrupting SMAD3/4 complex formation, thereby establishing a positive feedback loop.

### OTUD5 overexpression inhibits TGF-β-induced EMT and NSCLC cell metastasis in a TIF1γ-dependent manner

**Fig. 7:**
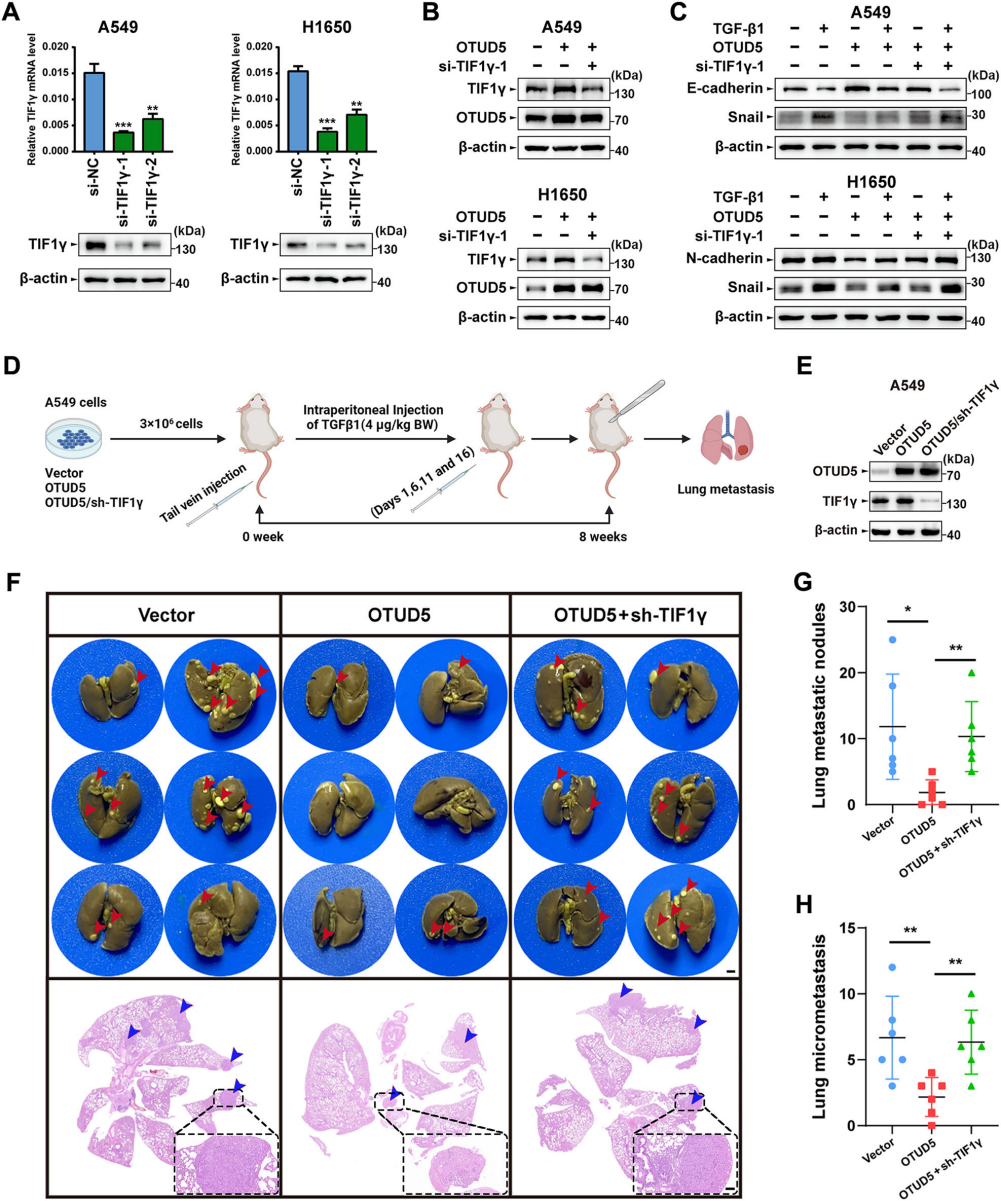
OTUD5 overexpression inhibits TGF-β-induced EMT and NSCLC cell metastasis in a TIF1γ-dependent manner. **A** RT-qPCR analysis of TIF1γ expression in A549 and H1650 cells transfected with specific siRNAs of TIF1γ (si-TIF1γ-1, si-TIF1γ-2) or scrambled sequence (si-NC). siRNA, small-interfering RNA. NC, negative control. Data are shown as the mean ± SD of *n* = 3 technical replicates. **P < 0.01 and ***P < 0.001 by unpaired Student’s *t* test. The experiment was repeated three times for confirmation (biological replicates). **B** si-TIF1γ-1-mediated knockdown of TIF1γ in OTUD5-overexpresing A549 and H1650 cells. **C** The above-mentioned A549 and H1650 cells were treated with TGF-β1 (5 ng/mL) as indicated for 24 h, and subjected to western blot analysis for determining the expression of EMT-related markers. **D** Schematic flowchart of NSCLC cell in vivo metastasis model. Vector control, OTUD5-overexpresing and OTUD5-overexpresing/sh-TIF1γ A549 cells (3 × 10^6^ cells/mouse) were intravenously injected into BALB/c nude mice. TGF-β1 was injected i.p. at days 1, 6, 11, and 16 after cell injection to facilitate TGF-β-stimulated A549 cell metastasis. **E** Before cell injection, Western blot was performed to confirm the efficiency of OTUD5 overexpression and TIF1γ knockdown. **F** Representative photographs of lung metastatic nodules and micrometastatic foci developed in mice 8 weeks after injection of A549 cells and TGF-β described above. The surgically resected lungs were fixed, stained and histologically examined as described in Materials and Methods. Red arrowheads indicate metastatic nodules established in lungs. Scale bar, 3 mm (top part). Blue arrowheads denote micrometastatic foci; the dashed box encloses an enlarged view (20×) of the metastatic focus; Scale bar, 100 μm (bottom part). **G**, **H** Dot plots showing the difference in lung metastatic nodules or micrometastasis counts between vector control, OTUD5-overexpresing and OTUD5-overexpresing/sh-TIF1γ groups (*n* = 6 mice per group). Data are shown as the mean ± SD, *P < 0.05 and **P < 0.01 by unpaired Student’s *t* test.

Our previous study demonstrated that TIF1γ knockdown promotes TGF-β-induced EMT and NSCLC cell metastasis [10]. Here, we found that OTUD5 not only inhibited TGF-β-induced EMT (Figs. 3B and S3B) but also blocked TGF-β/SMAD signaling by interacting with TIF1γ in NSCLC cells (Figs. 4–6). Therefore, we explored whether the inhibitory effects of OTUD5 on TGF-β-induced EMT and metastasis are dependent on TIF1γ. First, we knocked down TIF1γ in A549 and H1650 cells overexpressing OTUD5 (Fig. 7A, B) and examined EMT marker expression and invasive capacity in the presence or absence of TGF-β1. As a result, TGF-β-induced EMT (characterized by downregulation of E-cadherin and upregulation of N-cadherin and Snail) and invasion were reversed by OTUD5 overexpression; however, these effects were partially abrogated by TIF1γ knockdown (Figs. 7C and S6A–S6D). Second, we investigated whether OTUD5 inhibits NSCLC cell metastasis in vivo in a TIF1γ-dependent manner. Vector control, OTUD5-overexpressing, and OTUD5-overexpressing/sh-TIF1γ A549 cells were injected via tail vein into BALB/c nude mice, followed by intraperitoneal (i.p.) administration of TGF-β1 (Fig. 7D, E). Eight weeks post-inoculation, mice injected with OTUD5-overexpressing A549 cells developed fewer metastatic lung nodules and micrometastatic foci in lung tissues compared with controls. Importantly, this anti-metastasis effect was largely abrogated in mice injected with OTUD5-overexpressing/sh-TIF1γ A549 cells (Fig. 7F–H). Collectively, our results demonstrate that OTUD5 overexpression inhibits TGF-β-induced EMT and metastasis of NSCLC cells in a partially TIF1γ-dependent manner.

### Nilotinib directly binds to OTUD5 to augment TIF1γ deubiquitination and inhibits TGF-β-induced EMT and invasion of NSCLC cells

**Fig. 8:**
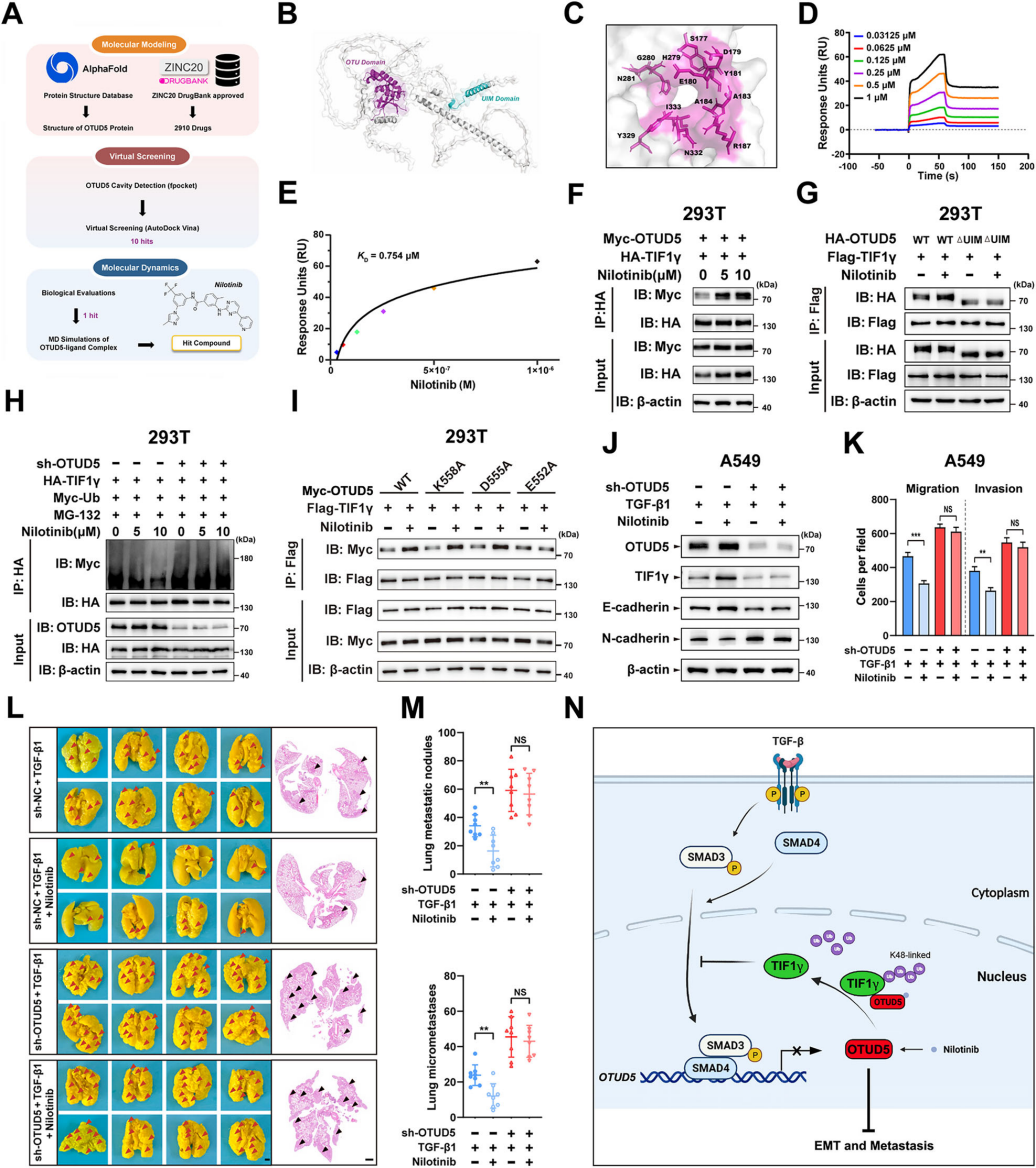
Nilotinib decreases TIF1γ ubiquitination by directly binding OTUD5 and suppresses TGF-β-induced EMT and NSCLC cell invasion and metastasis. **A** Computational workflow was performed for the virtual screening of ZINC20 DrugBank-approved drugs. **B** The modeled structure of OTUD5 available from AlphaFold Protein Structure Database. The structure is colored according to the functional domains with OTU domain in purple, and UIM domain in cyan. **C** The substrate-binding region of OTUD5 was detected by using fpocket, with 13 residues of the OTU domain colored in purple. **D** SPR analysis of the interaction of nilotinib with recombinant human OTUD5 protein. **E** The steady-state *K*_D_ value of SPR was calculated using Biacore insight evaluation software. **F** HEK 293 T cells co-transfected with Myc-OTUD5 and HA-TIF1γ were treated with nilotinib (0, 5, and 10 μM) as indicated for 12 h, and cell lysates were immunoprecipitated using anti-HA antibodies, followed by IB with indicated antibodies. **G** HEK 293 T cells were co-transfected with Flag-TIF1γ and either HA-OTUD5 (WT) or HA-OTUD5-ΔUIM. Nilotinib (10 μM) was administered 12 h prior to harvest. Cell lysates were then immunoprecipitated with anti-Flag beads and IB with the indicated antibodies. ΔUIM: OTUD5 with UIM domain deletion. **H** Analysis of TIF1γ ubiquitination in HEK 293 T cells co-transfected with sh-OTUD5, HA-TIF1γ, and Myc-Ub. Cell lysates were subjected to co-IP with anti-HA antibodies and IB with indicated antibodies after the cells were treated with MG-132 (10 μM) and nilotinib (0, 5, and 10 μM) for 6 h and 12 h, respectively. **I** HEK 293 T cells co-transfected with Myc-OTUD5 or its mutants and Flag-TIF1γ were treated with or without nilotinib (10 μM) for 12 h. Cell lysates were then immunoprecipitated using anti-Flag antibodies, followed by IB with the indicated antibodies. **J** Western blot analysis of EMT markers and TIF1γ expression in OTUD5-silenced A549 cells, which were treated with TGF-β1 (5 ng/mL) for 12 h, followed by co-treatment with nilotinib (10 μM) for another 12 h before being harvested. **K** OTUD5-silenced A549 cells were treated as above (**J**) and subjected to Transwell migration and invasion assays. After 24 h, the migrated and invasive cells were stained and counted in at least three light microscopic fields per well. Data are shown as the mean ± SD of *n* = 3 technical replicates. ns, not significant; **P < 0.01 and ***P < 0.001 by unpaired Student’s *t*-test. The experimen*t* was repeated three times for confirmation (biological replicates). **L** Representative photographs of lung metastatic nodules and micrometastatic foci developed in BALB/c nude mice 8 weeks after injection of control shRNA or sh-OTUD5 A549 cells, TGF-β1 (4 μg/kg), and/or nilotinib (20 mg/kg) as indicated. Red arrowheads denote metastatic nodules established in lungs. Scale bar, 3 mm (left part). Black arrowheads indicate micrometastatic foci. Scale bar, 1 mm (right part). **M** Dot plots showing the difference in lung metastatic nodules or lung micro-metastases counts between the indicated groups (*n* = 8 mice per group). Data are shown as the mean ± SD. ns, not significant; **P < 0.01 by unpaired Student’s *t* test. **N** A proposed model illus*t*rates how therapeutic targeting of the OTUD5-TIF1γ-SMAD3/4 positive feedback loop by nilotinib inhibits TGF‑β‑induced EMT and metastasis in NSCLC. Nilotinib directly binds to OTUD5 and enhances its interaction with TIF1γ, thereby promoting TIF1γ deubiquitination and attenuating TGF‑β/SMAD signaling. These effects collectively suppress TGF‑β‑driven EMT and NSCLC metastasis, revealing a previously unrecognized molecular mechanism underlying the anti‑tumor activity of nilotinib. Created with BioRender (https://biorender.com/).

Next, based on drug repurposing strategy, we performed a virtual screening to identify small compounds targeting OTUD5 protein (Fig. 8A). The receptors and ligands were retrieved from AlphaFold and ZINC20-DrugBank databases for their molecular docking in the most druggable binding pocket of OTUD5. The top 10 ranked compounds were selected for biological evaluation and molecular dynamics (MD) simulations. Structurally, OTUD5 comprises an OTU catalytic domain and a UIM domain (Fig. 8B). The binding pocket, displaying the highest druggable score, is located within the OTU domain (Fig. 8C), consisting of thirteen residues. Based on docking scores, ten compounds (temoporfin, nilotinib, bisoctrizole, paritaprevir, eribulin, ledipasvir, olysio, accolate, digitoxin, and midostaurin) exhibited favorable binding affinities (Table S3). Especially, temoporfin and nilotinib exhibited the strongest binding affinities to OTUD5. Thus, nilotinib, an FDA-approved TKI drug for CML [28, 29], was picked for further evaluation. Surface plasmon resonance (SPR)-based assay revealed that nilotinib directly binds to OTUD5 in a dose-dependent manner, with a dissociation constant (*K*_D_) of 0.754 μM (Fig. 8D, E). To validate the binding stability and refine the binding pose of nilotinib, we conducted 500-ns all-atom MD simulations. The nilotinib-bound OTU domain exhibited lower root-mean-square deviation (RMSD) compared with the apo state (Figure S7A), indicating enhanced structural stability. Reduced root-mean-square fluctuation (RMSF) values further confirmed decreased structural flexibility upon nilotinib binding (Figure S7B). Molecular mechanics-generalized born surface area (MM-GBSA) calculations with the 400-500 ns MD trajectory yielded a favorable binding free energy (Δ*G*_*binding*_) of -46.0822 kcal/mol, confirming high-affinity binding of nilotinib to OTUD5 (Figure S7C). Taken together, these results establish nilotinib as a high-affinity ligand for OTUD5.

Next, we examined the effects of nilotinib on TGF-β/SMAD signaling pathway. Nilotinib dose-dependently suppressed TGF-β/SMAD signaling activation in NSCLC cells, as evidenced by reduced expression of Snail, Slug, and PAI-1 (Figures S7D–S7F). Mechanistic investigations revealed that nilotinib specifically potentiated the OTUD5-TIF1γ interaction, an effect that was completely abolished upon UIM domain deletion (Figs. 8F, G). Furthermore, nilotinib enhanced TIF1γ deubiquitination; however, this effect was attenuated in OTUD5-silenced cells (Fig. 8H), indicating that OTUD5 mediates the nilotinib-induced deubiquitination of TIF1γ. To understand how nilotinib allosterically activates OTUD5, we generated residue interaction networks (RINs) from MD simulations of OTUD5 in both its apo (unbound) and holo (nilotinib-bound) states. By calculating changes in betweenness centrality (ΔBC = BC_holo_ – BC_apo_), we identified a significant rewiring of the protein communication network. The ubiquitin-interacting motif (UIM; residues 536-553) showed the largest increases in ΔBC (Figure S7G), suggesting that it becomes a central hub for signal transmission when nilotinib is bound. This observation was corroborated by the identification of a shorter, more direct allosteric pathway in the holo state that converges on UIM hotspot residues (K558, D555, and E552) (Figures S7H and S7I). Importantly, site-directed mutagenesis confirmed that the OTUD5/E552A mutation completely abolished nilotinib-induced OTUD5-TIF1γ interaction (Fig. 8I), confirming E552 as a critical mediator of nilotinib’s allosteric effect. Together, these results delineate the structural and dynamic basis for nilotinib-mediated allosteric activation of OTUD5. We next evaluated the anti-metastatic effects of nilotinib in NSCLC cells and mouse models. Nilotinib treatment efficiently inhibited TGF-β-induced EMT and invasion in A549 cells; however, these effects were abolished upon OTUD5 knockdown (Figs. 8J, K). Moreover, nilotinib showed stronger suppressive effects on TGF-β-driven EMT and invasion in OTUD5-overexpressing A549 cells (Figures S7J and S7K). Consistently, administration of nilotinib successfully attenuated lung metastasis of A549 cells in BALB/c nude mice (sh-OTUD5 control group). However, the therapeutic effect of nilotinib was abolished in the OTUD5-silenced group (Fig. 8L, M), demonstrating that OTUD5 is indispensable for the inhibitory effects of nilotinib on NSCLC cell invasion and metastasis. Immunohistochemical staining for p-SMAD3 indicated that the TGF-β pathway was activated in this model (Figure S7L). Notably, the nilotinib concentrations used did not affect cell viability, and in vivo experiments confirmed that nilotinib treatment caused no detectable toxicity to major organs, including heart, liver, spleen, lung, and kidney (Figures S7M and S7N).

## Discussion

Although our previous studies demonstrated that TIF1γ inhibits TGF-β-induced EMT and metastasis of NSCLC cells [10, 15], the post-translational regulators of TIF1γ remain largely uncharacterized. Here, we identify OTUD5, a deubiquitinase of the OTU family, as a metastasis-suppressive factor in NSCLC. OTUD5 stabilizes TIF1γ through direct deubiquitination, thereby attenuating TGF-β/SMAD signaling. Mechanistically, the TGF-β-activated SMAD3/4 complex transcriptionally represses OTUD5 expression. Conversely, OTUD5 interacts with and deubiquitinates TIF1γ, enhancing its stability and thereby attenuating TGF-β-induced formation of SMAD3/4 complex. This, in turn, impairs TGF-β-induced inhibition of *OTUD5* transcription. Functionally, we reveal that OTUD5 represses TGF-β-induced EMT and metastasis of NSCLC cells in a manner that is partially, but not exclusively, dependent on TIF1γ. Our study reveals an OTUD5-TIF1γ-SMAD3/4 positive feedback loop that restrains TGF-β/SMAD signaling in NSCLC metastasis and highlights nilotinib as a promising therapeutic strategy (Fig. 8N).

Consistent with a tumor-suppressive role, OTUD5 is significantly downregulated in NSCLC tissues [25, 26] and low OTUD5 expression correlates strongly with poor patient prognosis [39]. Our data corroborate these findings and further demonstrate pronounced OTUD5 loss in metastatic NSCLC specimens (Fig. 1A, C, D, and S1G). Integrating this with our earlier observation that SMAD3/4 transcriptionally activates EMT-related genes in NSCLC [9, 11], we hypothesized that the same complex might repress OTUD5. Indeed, we confirm that TGF-β-induced SMAD3/4 directly suppresses OTUD5 transcription (Fig. S2A, S2B, and 2A–M), and that OTUD5 overexpression potently inhibits TGF-β-induced EMT and metastasis in vitro and in vivo (Figs. 3A–G, S3A–S3G, and 7C–7H). Notably, our in vivo assays employed a tail-vein injection model, which primarily recapitulates late-stage metastatic events (extravasation and lung colonization). Future studies using orthotopic or spontaneous metastasis models will be essential to evaluate the role of the OTUD5-TIF1γ axis in early dissemination steps. Additionally, incorporating TGF-β-insensitive cell lines as negative controls would further validate the specificity of these findings.

Through MS-based proteomics, co-IP, and pull-down analyses, we found that OTUD5 directly interacts with, deubiquitinates, and stabilizes TIF1γ (Figs. 4A–L, and 5E–L). Given that TIF1γ antagonizes TGF-β/SMAD signaling by impairing SMAD3/4 complex formation [13, 14, 38], it is significant that OTUD5 recapitulates this effect by disrupting SMAD3/4 assembly in a TGF-β-dependent manner (Fig. 6A–C). Importantly, the inhibitory effect of OTUD5 on TGF-β/SMAD signaling depends on TIF1γ, and TIF1γ impedes TGF-β-induced repression of *OTUD5* transcription (Fig. 6D–J). Although TIF1γ possesses E3 ubiquitin ligase activity [35, 36], it does not ubiquitinate or promote degradation of OTUD5 (Fig. 5O). This observation supports the existence of an OTUD5-TIF1γ-SMAD3/4 positive feedback loop that impedes TGF-β/SMAD signaling.

A critical question arising from these observations is whether OTUD5 suppresses TGF-β-induced EMT and metastasis in a TIF1γ-dependent manner. To address this, we performed functional experiments and found that TIF1γ knockdown significantly, but not completely, reversed the inhibitory effects of OTUD5 on TGF-β-induced EMT and metastasis (Figs. 7 and S6), suggesting that this effect of OTUD5 partially depends on TIF1γ. Notably, our proteomic screen identified several additional OTUD5-interacting proteins, including GRB2 and CDKN2A [40–42], which may contribute to TIF1γ-independent anti-metastatic activity of OTUD5. To our knowledge, this study is the first to report functional cooperation between OTUD5 and TIF1γ, providing mechanistic evidence that OTUD5 acts as a tumor-suppressing DUB in NSCLC. This finding aligns with published literature showing that reduced TIF1γ promotes TGF-β-dependent metastasis in human cancers [10, 38, 43].

The role of OTUD5 appears strikingly context-dependent. While it acts as an oncogene in breast, bladder, and hepatocellular carcinomas [44–46], it functions as a metastasis suppressor in NSCLC [25, 26, 39]. This context-dependent duality likely reflects differences in substrate availability, E3 ligase networks, and the tumor microenvironment [47]. In NSCLC, where TGF-β signaling drives metastasis, and TIF1γ serves as a critical negative regulator of this pathway [10, 48], OTUD5-mediated stabilization of TIF1γ results in a net tumor-suppressive effect. By contrast, in tumor types where OTUD5 preferentially stabilizes oncogenic substrates, its activity may instead promote tumor progression [44–46]. Thus, the biological output of OTUD5 is determined by the broader signaling context rather than its intrinsic catalytic activity alone.

Interestingly, using a structure-based pharmacophore modeling combined with molecular docking, we identified nilotinib, an FDA-approved TKI drug for CML [28, 29], as a potential regulator of OTUD5. Our experimental data demonstrate that nilotinib significantly inhibited TGF-β-induced EMT, invasion, and metastasis in NSCLC cells, which is abrogated upon OTUD5 knockdown (Fig. 8J–M). While nilotinib was previously reported to suppress colorectal cancer metastasis by antagonizing DDR1 [49], our data reveal a distinct, kinase-independent mechanism in NSCLC: nilotinib acts as a functional agonist of OTUD5, promoting TIF1γ deubiquitination and subsequent attenuation of TGF-β/SMAD signaling (Fig. 8N). Although our genetic data support OTUD5 as a functionally relevant target in this model (Figs. 8 and S7), we cannot completely exclude potential contributions from inhibition of nilotinib’s canonical kinase targets [28, 50]. These pharmacological considerations warrant further investigation in preclinical and clinical studies. Furthermore, clinical studies will be necessary to determine whether sex influences the efficacy or toxicity of OTUD5-targeting therapies, including nilotinib.

In summary, our study establishes an OTUD5-TIF1γ-SMAD3/4 positive feedback loop by which OTUD5 suppresses TGF-β-induced EMT and metastasis of NSCLC cells, providing new insights into the regulation of TGF-β/SMAD signaling by deubiquitination. Furthermore, we identify nilotinib as a promising high-affinity OTUD5-targeting drug candidate for the treatment of metastatic NSCLC.

## Materials and methods

All detailed protocols were described in the Supplementary Experimental Procedures.

### ETHICS

All methods were carried out in accordance with the Declaration of Helsinki. This study has been approved by the Ethics Committee of Soochow University.

## Supplementary information

Supplementary Figures S1-S7 and Figure Legends, and Supplementary Experimental Procedures

Supplementary Tables S1-S5

Checklist

Uncropped original western blots

## Data Availability

The data analyzed in Figs. 1A, 1B, and S1A–S1N are available from TCGA (https://www.cancer.gov/ccg/ research/genome-sequencing/tcga) and GTEx (https://www.gtexportal.org/) databases. The raw data of MS-based proteomics generated in Table S2 have been deposited into the ProteomeXchange Consortium (https://proteomecentral.proteomexchange.org) via the iProX partner repository with the dataset identifier PXD067457. GSEA data are accessible at the GEO repository (https://www.ncbi.nlm.nih.gov/geo/) under accession number GSE19804.
