# Supplementary material for: OTUD5-TIF1γ-SMAD3/4 positive feedback loop inhibits TGF-β-induced EMT and metastasis in NSCLC
Source: Cell Death Dis. 2026 May 25;17(1):650. doi: 10.1038/s41419-026-08901-z (PMC13385805; doi:10.1038/s41419-026-08901-z)
Supplement: Supplementary file 1 — Supplementary Figures S1-S7 and Figure Legends, and Supplementary Experimental Procedures [file 41419_2026_8901_MOESM1_ESM.pdf]

Supplementary Figures and Figure Legends

Figure S1

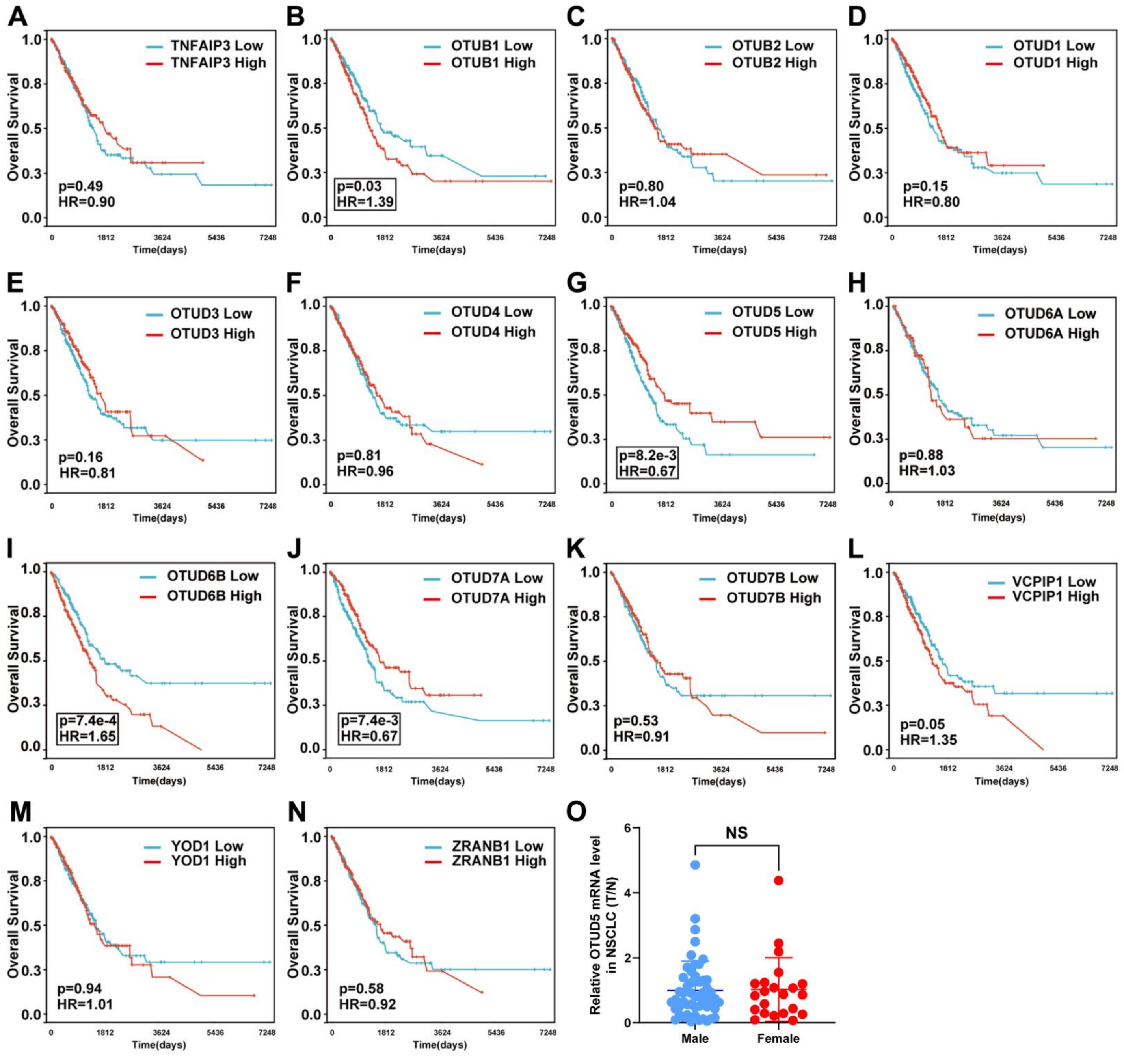

Figure S1. Association of OTUs' expression with overall survival of NSCLC patients and the expression of OTUD5 between male and female. (**A**-**N**) Kaplan-Meier survival curves of NSCLC individuals (n = 502, from TCGA database) with low or high expression of 14 OTUs. *P* values were used to compare the differences between two groups. (**O**) The relative mRNA expression (T/N) of OTUD5 in male (n = 52) and female (n = 22) NSCLC tissues. T, NSCLC tumor tissues; N, para-carcinoma tissues. Data are shown as the mean ± SD; ns, not significant. The experiment was repeated three times for confirmation (biological replicates).

Figure S2

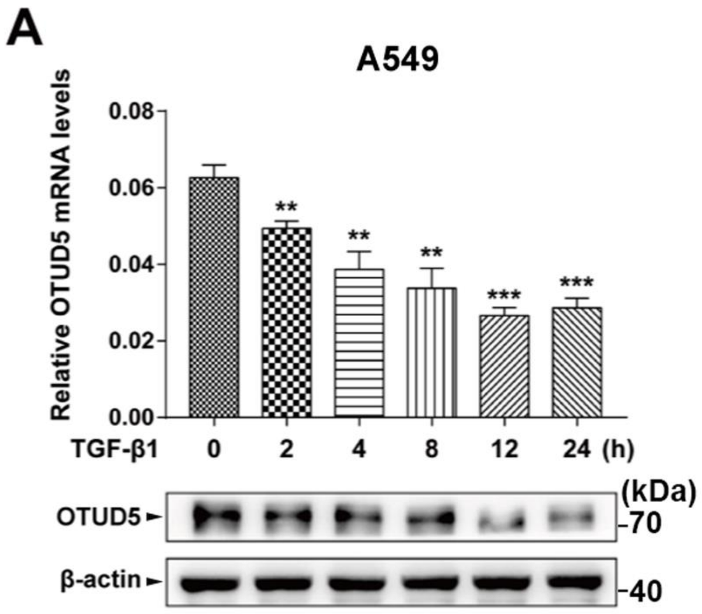

Figure S2. OTUD5 expression is inhibited by TGF-β1. (**A** and **B**) A549 and H1650 cells were treated with TGF-β1 (5 ng/mL) for the indicated times, and subjected to RT-qPCR and western blot analysis for detection of OTUD5 expression. β-actin served as a loading control. Data are shown as the mean ± SD of n = 3 technical replicates, $*P < 0.05$, $**P < 0.01$ and $***P < 0.001$ by unpaired Student's *t*-test. The experiment was repeated three times for confirmation (biological replicates).

Figure S3

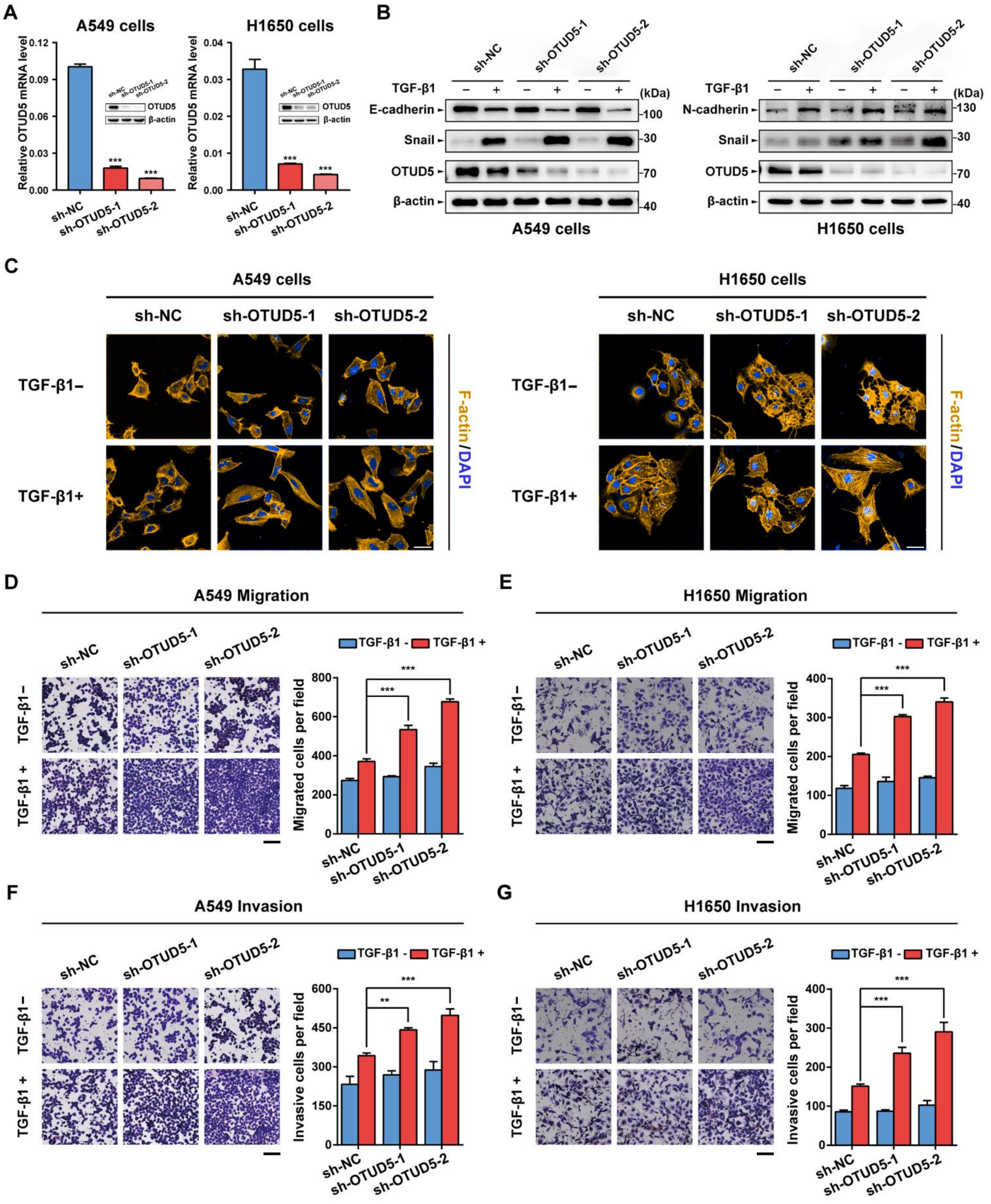

Figure S3. OTUD5 knockdown promotes TGF-β-induced EMT and invasion of NSCLC cells. (**A**) RT-qPCR and western blot analyses of OTUD5 expression in OTUD5-silenced A549 and H1650 cells. β-actin served as a

loading control. Data are shown as the mean ± SD of n = 3 technical replicates, ***$P < 0.001$ by unpaired Student's *t*-test. The experiment was repeated three times for confirmation (biological replicates). (**B**) After being serum starved, OTUD5-silenced A549 and H1650 cells were treated with or without TGF-β1 (5 ng/mL) for 24 h. Then, western blot was conducted to determine the expression of the indicated EMT markers. (**C**) OTUD5-silenced A549 and H1650 cells and control cells were treated as above, and F-actin (orange) was stained with phalloidin-iFluor™ 555 reagent. Cell nuclei were counterstained and visualized with DAPI (blue). Scale bar, 100 μm. (**D-G**) OTUD5-silenced A549 and H1650 cells were treated as above and allowed to migrate through an 8-μm pore membrane (D and E) or invade through a Matrigel-coated membrane (F and G) in Transwells. After 24 h, the migratory and invasive cells were stained, photographed, and counted in at least three light microscopic fields per well. Representative images and the migratory and invasive cell numbers were shown. Scale bar, 100 μm. Data are shown as the mean ± SD of n = 3 technical replicates, **$P < 0.01$ and ***$P < 0.001$ by unpaired Student's *t*-test. The experiment was repeated three times for confirmation (biological replicates).

Figure S4

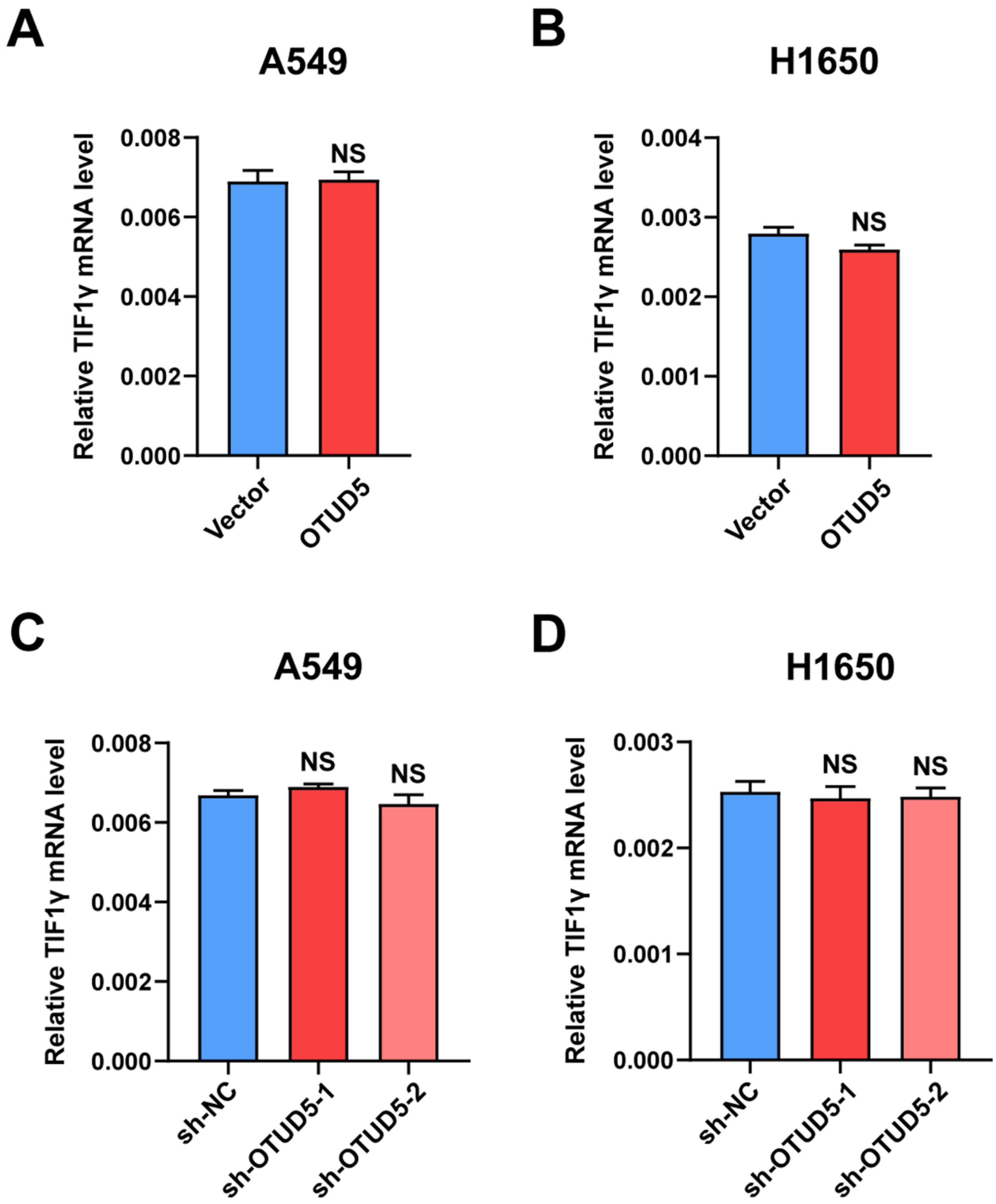

Figure S4. OTUD5 does not change TIF1γ mRNA expression in NSCLC cells. (**A-D**) RT-qPCR analysis of TIF1γ mRNA expression in OTUD5-overexpressing/silenced A549 and H1650 cells. Data are shown as the mean ± SD of n = 3 technical replicates. ns, not significant by unpaired Student's *t*-test. The experiment was repeated three times for confirmation (biological replicates).

Figure S5

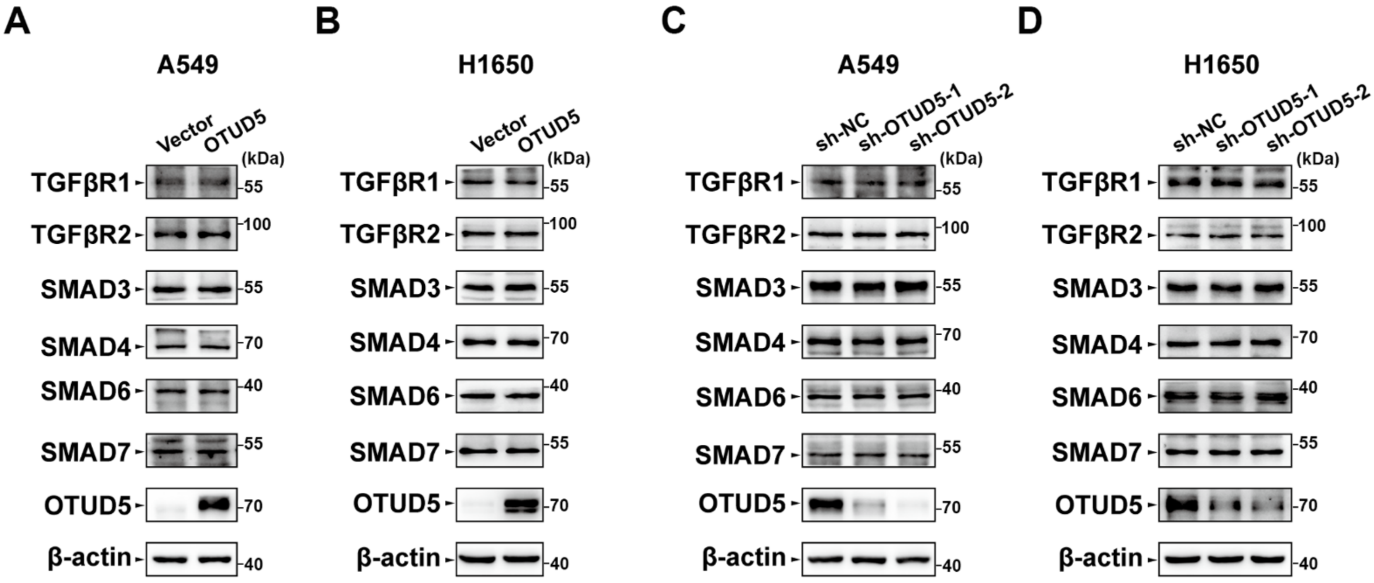

Figure S5. OTUD5 fails to alter protein levels of key components regulating TGF-β/SMAD signaling. (**A-D**) Western blot analysis for the expression of several proteins, including TGFβR1/2, SMAD3/4, and SMAD6/7, in OTUD5-overexpressing/silenced A549 and H1650 cells. β-actin served as a loading control.

Figure S6

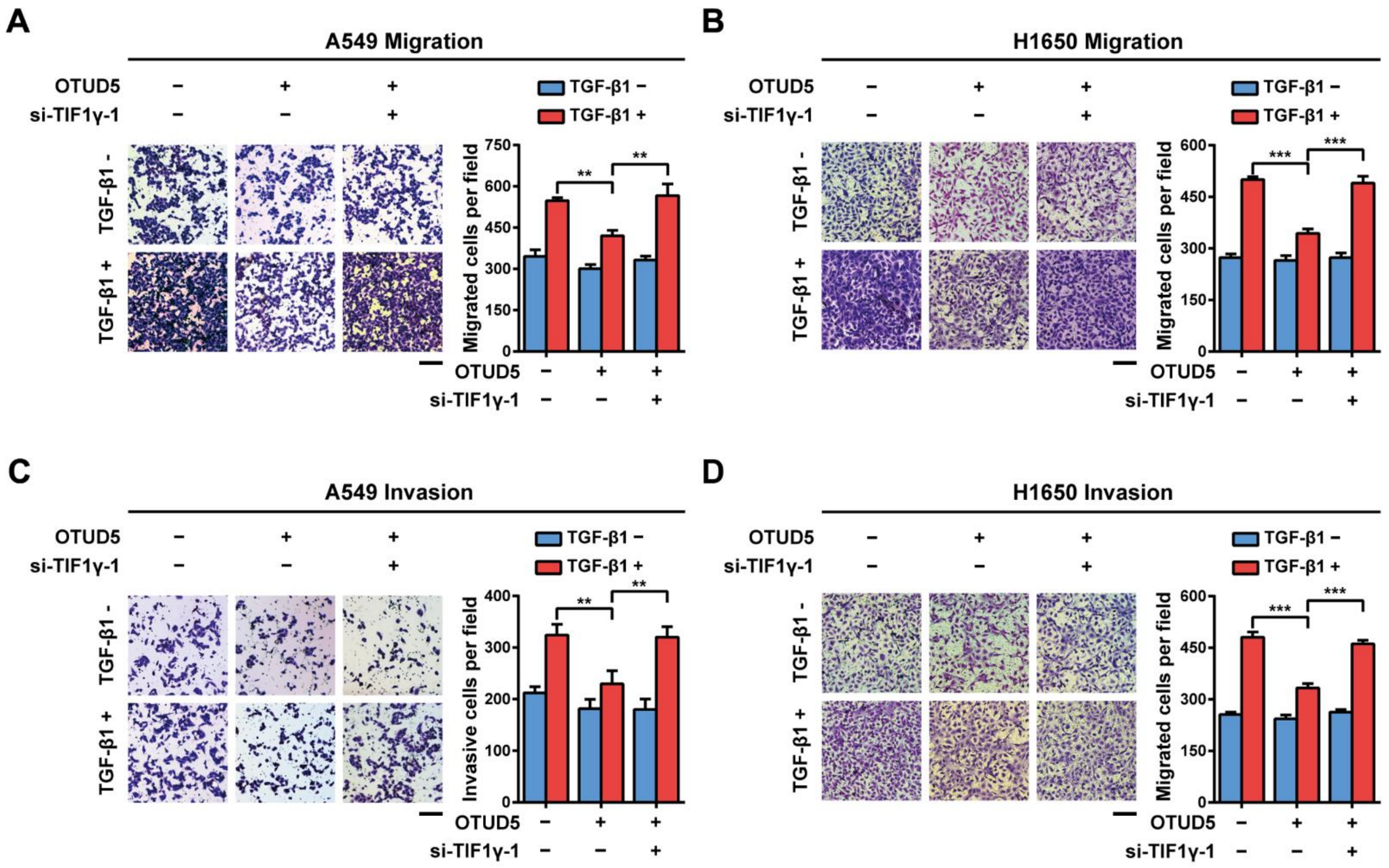

Figure S6. OTUD5 overexpression inhibits TGF-β-induced NSCLC cell invasion in a TIF1γ-dependent manner. (**A-D**) A549 and H1650 cells overexpressing OTUD5 and/or transfected with si-TIF1γ-1 (Figure 7B) were treated with or without TGF-β1 as indicated. Then, cells were allowed to migrate through an 8-μm pore membrane (A and B) or invade through a Matrigel-coated membrane (C and D) in Transwells. After 24 h, the migratory and invasive cells were stained, photographed, and counted in at least three light microscopic fields per well. Representative images and the migratory and invasive cell numbers were shown. Scale bar, 100 μm. Data are shown as the mean ± SD of n = 3 technical replicates, ***P* < 0.01 and ****P* < 0.001 by unpaired Student's *t*-test. The experiment was repeated three times for confirmation (biological replicates).

Figure S7

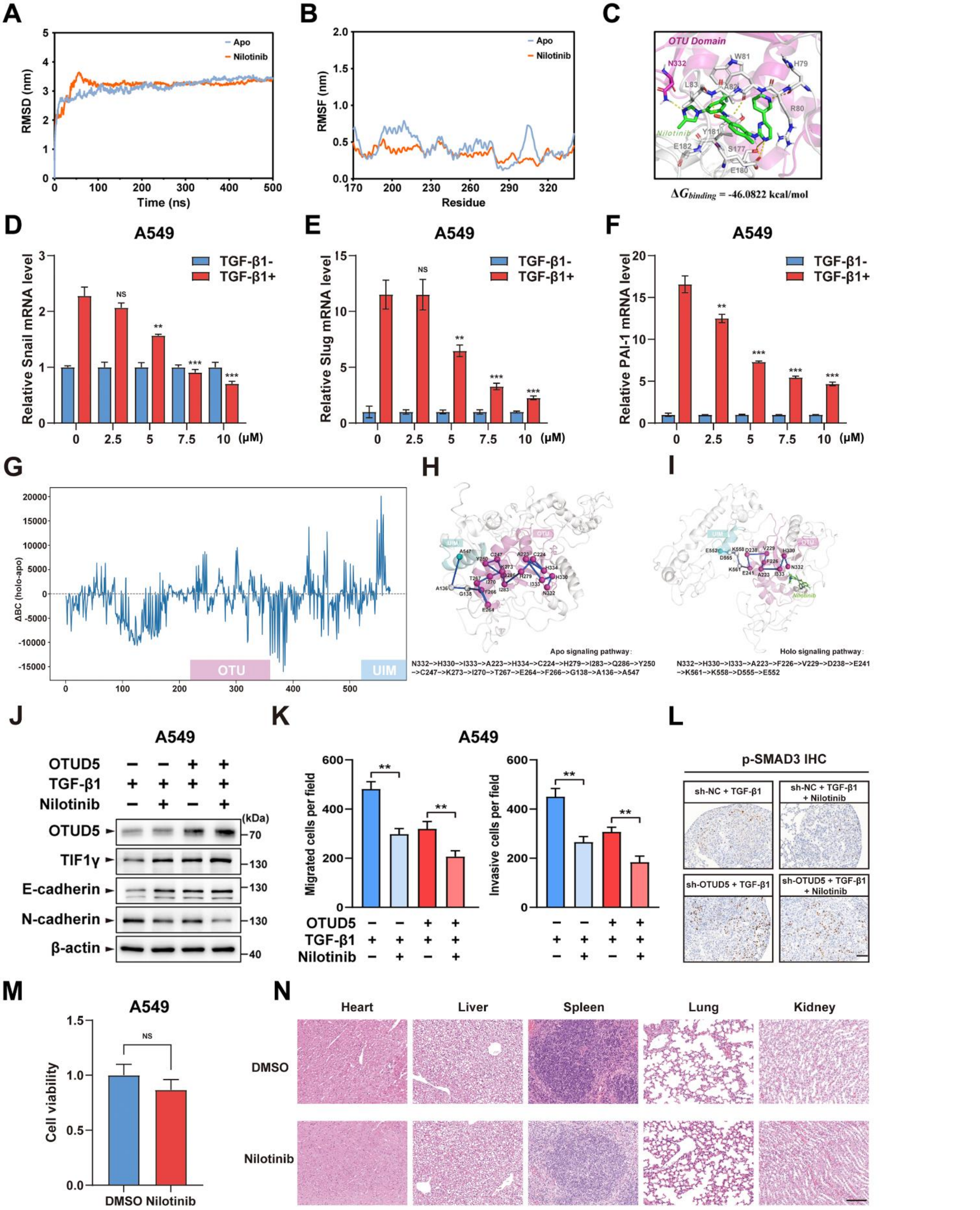

Figure S7. Nilotinib decreases TGF-β/SMAD signaling activation and exerts significant anti-metastasis effects on NSCLC cells. (**A** and **B**) RMSD and RMSF analysis of the OTU domain in the apo and nilotinib-bound

OTUD5 systems. apo, ligand-free state. **(C)** The binding pattern of nilotinib and OTUD5 protein at 500 ns of MD simulations. Nilotinib (green) forms hydrogen bonds (yellow dashed lines) with key interacting residues of OTU domain (H79, A82, L83, S177, E180, and N332, which are represented as sticks). Based on the 400-500 ns MD simulation trajectory, the binding free energy ($\Delta G_{binding}$) between nilotinib and OTUD5 was calculated using MM/GBSA method. (**D-F**) RT-qPCR analysis of Snail, Slug and PAI-1 mRNA expression in A549 cells. Cells were treated with TGF-β1 (5 ng/mL) for 12 h, followed by co-treatment with nilotinib (0, 2.5, 5, 7.5, 10 μM) for another 12 h before being harvested. Data are shown as the mean ± SD of n = 3 technical replicates. ns, not significant; $^{**}P < 0.01$ and $^{***}P < 0.001$ by unpaired Student's *t*-test. The experiment was repeated three times for confirmation (biological replicates). (**G**) ΔBC analysis revealed that upon nilotinib binding, the communication network of OTUD5 underwent significant rewiring, with the UIM domain (residues 536-553) displaying the strongest positive ΔBC peaks across the entire protein. BC, betweenness centrality. apo, ligand-free state; holo, nilotinib-bound state. (**H**) Conformational pathways from the OTU domain to the UIM domain in the apo state. (**I**) Conformational pathways from the OTU domain to the UIM domain in the holo state. (**J**) Western blot analysis of EMT markers and TIF1γ expression in OTUD5-overexpressing A549 cells. Cells were treated with TGF-β1 (5 ng/mL) for 12 h, followed by co-treatment with nilotinib (10 μM) for another 12 h before cell harvesting. (**K**) OTUD5-overexpressing A549 cells were treated as above (J) and subjected to Transwell assays. After 24 h, the migrated and invasive cells were counted in at least three fields per well. Data are shown as the mean ± SD of n = 3 technical replicates, $^{**}P < 0.01$ by unpaired Student's *t*-test. The experiment was repeated three times for confirmation (biological replicates). (**L**) Immunohistochemical staining for p-SMAD3 was used to evaluate TGF-β pathway activation. Scale bar: 100 μm. (**M**) Cell viability was assessed in A549 cells treated with nilotinib (10 μM) for 12 h using a CCK-8 assay. Data are shown as the mean ± SD; ns, not significant. The experiment was repeated three times for confirmation (biological replicates). (**N**) To evaluate nilotinib toxicity, BALB/c nude mice were injected intraperitoneally with 20 mg/kg nilotinib (dissolved in saline) every other day for 8 weeks. HE staining was used to examine the heart, liver, spleen, lung, and kidney of nilotinib-treated and untreated mice. Scale bar: 100 μm.

Supplementary Experimental Procedures

Cell culture

Human embryonic kidney (HEK) 293T cell line, and NSCLC cell lines A549 and H1650 were purchased from the Cell Bank of Chinese Academy of Science. These cell lines were authenticated with STR profiling and checked free of mycoplasma contamination by PCR. A549 and H1650 cells were cultured in RPMI 1640 medium (Basal Media, L210KJ) and HEK 293T cells in Dulbecco's modified Eagle medium (DMEM, UUBIO, U11004) with 10% fetal bovine serum (FBS; ABW, AB-FBS0500S) at 37°C in a 5% CO_2 humidified incubator. A549 and H1650 cells were treated by TGF-β1 (5 ng/mL, PeproTech, 100-21) to undergo EMT following serum-starvation for 24 h. TGF-β1 was reconstituted in 10 mM Citric Acid containing 0.1% bovine serum albumin to maintain activity.

Human NSCLC tissue samples

Seventy-four fresh-frozen carcinoma tissues and their matched adjacent para-carcinoma tissues were collected after informed consent from NSCLC patients in the First Affiliated Hospital of Soochow University (Suzhou, China). NSCLC patients were pathologically diagnosed and evaluated according to the Revised International System for Staging Lung Cancer. The detailed clinical information of 74 NSCLC patients is described in Table S1, metastatic tissues (n = 37) were from NSCLC patients with local lymph node metastasis ($T_{1–4}N_{1–2}M_0$) or distant organ metastasis ($T_{1–4}N_{any}M_1$), and non-metastatic tissues (n = 37) were from NSCLC patients without any metastasis ($T_{1–4}N_0M_0$). None of patients had received radiotherapy or chemotherapy before surgical treatment. This study was authorized by the Ethics Committee of Soochow University (2023 Ethics Approval No. 241).

Gene set enrichment analysis (GSEA)

The software GSEA 4.0.3 (Broad Institute, Cambridge, USA) was performed to analyze the correlation of OTUD5 expression with three gene sets, including epithelial cell migration, EMT, and TGF-β signaling pathway. GSE19804 dataset including 60 lung cancer specimens was downloaded from Gene Expression Omnibus (GEO), which were divided into high and low subgroups by OTUD5 median expression value. For each analysis, the gene set permutations were run for 1,000 times. Gene sets were considered significantly enriched with predefined values of $p < 0.05$, FDR < 0.25, and |Normalized ES| > 1.

Construction of vectors

The pcDNA3.1-TIF1γ-Flag and pcDNA3.1-truncated TIF1γ-Flag transient expression vectors, and pCDH-TIF1γ stable expression vector were generated as previously described by us [1]. OTUD5 CDS (GeneBank Accession number: NM_017602) was synthesized (GENEWIZ) and subcloned into pcDNA3.1-HA and pcDNA3.1-Myc vectors with endonucleases *Nhe*I/*Age*I (New England Biolabs, NEB; R0131L/R0552L) to yield pcDNA3.1-OTUD5-HA and pcDNA3.1-OTUD5-Myc expression vectors. OTUD5 CDS was also separately subcloned into pCDH-CMV-MCS-EF1-CopGFP-T2A-Puro lentiviral vector (Miaolingbio, P0268P0376) and pGEX-4T-2 vector with N-terminal GST-tag to produce pCDH-OTUD5 and pGEX-GST-OTUD5. Truncated CDS domains of OTUD5 were amplified with the corresponding PCR primers (Table S4) and subcloned into pcDNA3.1-HA vector to generate various expression vectors. The mutant vector pcDNA3.1-OTUD5(C224S)-Myc was generated using PCR-based site-directed mutagenesis and ClonExpress Ultra One Step Cloning Kit (C115-01, Vazyme). All plasmids were verified by sequencing before expression. Short hairpin RNAs (shRNAs) against TIF1γ and OTUD5 were synthesized and subcloned into a lentiviral vector pLKO.1-puro (GENEWIZ) to create pLKO.1-sh-TIF1γ and pLKO.1-sh-OTUD5, respectively. The shRNA sequences are listed in Table S4.

Generation of stable cell lines

The above-mentioned lentiviral vectors pCDH-OTUD5, pCDH-TIF1γ, pLKO.1-sh-OTUD5, and pLKO.1-sh-TIF1γ were respectively co-transfected with packaging plasmids psPAX2 and pMD2.G (RIBOBIO) into HEK 293T cells using Polyethylenimine Linear (PEI; APExBIO, K1029) for 48 h. The empty vectors or negative control shRNA (sh-NC) were used as negative controls. Packaged lentiviruses were collected and used to infect NSCLC or HEK 293T cells for 72 h. The stable cells were selected with 1 μg/mL puromycin (Yeasen, 60210ES25) for one week.

RNA interference

Short interfering RNAs (siRNAs) targeting TIF1γ (si-TIF1γ-1 and si-TIF1γ-2) and SMAD4 (si-SMAD4) were designed and synthesized (GenePharma). A scrambled siRNA served as negative control (si-NC). The sequences of siRNAs are listed in Table S4. Cells were transiently transfected with 100 pmol of siRNAs using Lipofectamine 3000. After transfection for 48 h, the cells were collected for further experiments.

RNA extraction, cDNA synthesis, and real-time quantitative reverse transcriptase PCR (RT-qPCR)

Total RNA from cells or tissues was extracted with TRIzol (Thermo Fisher Scientific, 15596018) according to

the manufacturer's protocol. cDNA synthesis and RT-qPCR analysis were as previously described by us [1]. Primers used for RT-qPCR analysis are listed in Table S4. Relative expression of mRNAs was determined by the $\Delta\Delta Ct$ method and each RT-qPCR analysis was conducted in triplicate. Each RT-qPCR analysis was repeated three times for confirmation (biological replicates).

Western blot analysis

Total protein was isolated from cell lysate and subjected to western blot analysis in accordance with our previous methods [2]. The primary antibodies employed in immunoblot assay were as follows: rabbit anti-OTUD5 (CST, 20087, 1:1000), mouse anti-E-cadherin (BD Biosciences, 610181, 1:1000), mouse anti-N-cadherin (BD Biosciences, 610920, 1:1000), mouse anti-Snail (CST, 3895, 1:1000), rabbit anti-SMAD3 (CST, 9523, 1:1000), rabbit anti-p-SMAD3 (Abcam, ab52903, 1:1000), rabbit anti-SMAD4 (CST, 46535, 1:1000), rabbit anti-TGFβR1 (Proteintech, 30117-1-AP, 1:1000), mouse anti-TGFβR2 (Santa Cruz, sc-17799, 1:500), mouse anti-SMAD6 (Santa Cruz, sc-25321, 1:500), rabbit anti-SMAD7 (Proteintech, 25840-1-AP, 1:1000), rabbit anti-TIF1γ (CST, 90051, 1:1000), rabbit anti-HA (CST, 3724, 1:1000), rabbit anti-Flag (CST, 14793, 1:1000), mouse anti-Myc (Proteintech, 60003-2-Ig, 1:1000), and mouse anti-β-actin (AB clonal, AC004, 1:1000). The secondary antibodies were goat anti-mouse (Santa Cruz, sc-2005, 1:3000) or mouse anti-rabbit antibodies (Santa Cruz, sc-2357, 1:3000). Relative expression of each protein was normalized to β-actin. Each Western blot analysis was repeated three times for confirmation (biological replicates), and representative bands were presented.

Co-immunoprecipitation (Co-IP)

Co-IP assay was performed as described [1] with some modifications. Cell lysates were respectively incubated with anti-FLAG M2 and anti-HA magnetic beads (Sigma-Aldrich, M8823; Thermo Scientific, 88836) at 4°C overnight. Besides, cell lysates were also incubated with antibodies against OTUD5 or TIF1γ at 4°C overnight and Protein A/G magnetic beads (Thermo Fisher Scientific, 88802) at room temperature for 2 h. The captured immune complexes were washed with IP buffer and subjected to immunoblot.

Protein silver-staining assay

The anti-HA co-IP products from HEK 293T cells transfected with HA-OTUD5 were separated on SDS-PAGE gel and then identified with Silver Staining Kit (CWBIO, CW2012). The detailed protocol was performed as previously described [1].

Mass spectrometry assay

The aforementioned IP protein mixture from HEK 293T cells overexpressing HA-OTUD5 was subjected to MS assay as described previously [3]. Data of MS-based analysis are described in Table S2.

GST pull-down

The pGEX-GST-OTUD5 or pGEX-4T-2 with N-terminal GST-tag (empty vector) plasmids were transformed into *E. coli* strain BL21 cells. Cells were cultured at 37°C overnight, followed by induction with 0.1 mM isopropyl-β-D-1-thiogalactopyranoside (IPTG) at 16°C with vigorous shaking for 12 h, and harvested and sonicated on ice and incubated with glutathione-Sepharose beads (Sangon Biotech, C600327). Purified GST-OTUD5 or GST proteins coupled to the beads were mixed with lysates from HEK 293T cells overexpressing Flag-TIF1γ. The beads and lysates mixture were incubated overnight at 4°C with rotation. After washing three times, bound proteins were separated on SDS-PAGE gel and visualized by immunoblot with anti-Flag or anti-OTUD5 antibodies and Coomassie Blue staining.

Chromatin immunoprecipitation (ChIP)

ChIP analysis was carried out with ChIP-IT Express Kit (Active Motif, 53008), which was depicted earlier by us [4]. In this ChIP analysis, the sheared chromatin fraction from cell nuclei was incubated with Protein G magnetic beads and anti-p-SMAD3 or SMAD4 antibodies overnight at 4°C. The ChIP DNA was purified and subjected to PCR amplification with *OTUD5*-specific primers (Table S4).

Immunofluorescence (IF) staining

Cells were seeded on coverslips in a 24-well culture plate. After 24 h, cells were fixed with 4% paraformaldehyde for 30 min, washed with PBS buffer, and incubated with 5% BSA for 1 h. Then, cells were incubated with mouse anti-TIF1γ (Santa Cruz, sc-101179) or rabbit anti-OTUD5 (CST, 20087) primary antibodies (1:300 dilution for both) at 4°C overnight, washed with PBST buffer, and incubated with fluorescein isothiocyanate (FITC)-conjugated anti-mouse secondary antibody (Beyotime, A0568) and Cy3-conjugated anti-rabbit secondary antibody (Beyotime, A0516) at room temperature for 2 h. Alternatively, F-actin protein was stained with iFluor 555-labeled phalloidin (Yeasen, #40737ES75). Nuclei were counterstained with 4′,6-diamidino-2-phenylindole (DAPI). Finally, protein subcellular localization was observed and photographed using a confocal laser

microscope (Carl Zeiss).

Transwell migration and invasion assays

Transwell migration and invasion assays were conducted as previously described by us [5, 6] with somewhat modification. Briefly, the upper chambers with 8-μm pore-permeable or Matrigel-coated membrane (Corning, 356234) were placed on 24-well plates (Corning, 35309) containing 20% FBS medium. Then, 5×10^4 cells were seeded on top of the insert in 1% FBS medium. Six hours later, 5 ng/mL TGF-β1 (PeproTech, 100-21-10) was added to the lower chambers. After incubation at 37°C for 24 h, the inserts were taken out and cells on the upper surface was removed with cotton swabs. Cells that migrated or invaded to the lower side were fixed and stained with 1% crystal violet (Solarbio Life Sciences, C8470). Cells were photographed and counted in at least three random microscopic fields per well. Each experiment was performed with three biological replicates.

Luciferase reporter assay

TGF-β1-induced transcriptional reporter assay was performed as described by us [4, 5]. Briefly, various lengths of OTUD5 promoter (OP) (Table S5) were subcloned into pGL3-Luc reporter vector (Promega, E1751) to form pGL3-OP1/2/3/4-Luc and pGL3-ΔOP-wild type/mutant-Luc constructs. Then, the aforementioned constructs and pRL-TK plasmid were co-transfected into A549 cells. After treatment with or without TGF-β1 for 24 h, cells were harvested and luciferase activities were determined with a Dual-Luciferase Reporter Assay System (Promega, E1910). Results are presented as relative luciferase activities (firefly/*Renilla*) and each luciferase reporter analysis was done in triplicate.

***In silico* docking**

Protein 3D-docking structure of OTUD5 and TIF1γ (PB domain) was predicted using ColabFold v1.5.3 [7] and modeled with AlphaFold2 Multimer [8]. Visualization of the protein 3D structure was performed using PyMol v3.0.3 software.

Virtual screening and molecular dynamics (MD) simulations

The structure of OTUD5 protein was retrieved from the AlphaFold Protein Structure Database [9]. The virtual screening using ZINC20-DrugBank database obtained a total of 2,910 FDA-approved drugs. Subsequently, the potential binding pocket region of OTUD5 was defined using fpocket [10], followed by molecular docking via

AutoDock Vina [11]. MD simulations of 500 ns were conducted with GROMACS [12] and Amber force field with GAFF parameters [13]. The stability of the simulation systems was assessed using root-mean-square deviation (RMSD) and root-mean-square fluctuation (RMSF). Post-MD simulations, the binding free energy ($\Delta G_{binding}$) of a protein-ligand complex was calculated by molecular mechanics-generalized born surface area (MM-GBSA) approach [14].

Surface plasmon resonance (SPR) analysis

SPR analysis of the binding of OTUD5 to nilotinib (TargetMol, T1524) was performed on a Biacore 1K instrument with CM5 sensor chip (Cytiva, Sweden). Briefly, recombinant human OTUD5 protein (CUSABIO, CSB-BP850298HU) was directly immobilized on an activated CM5 sensor chip by amine coupling. Then, gradient concentrations of nilotinib (0.03125 μM, 0.0625 μM, 0.125 μM, 0.25 μM, 0.5 μM, and 1 μM) were injected into the OTUD5-immobilized CM5 chip channel at a flow rate of 30 μL/min. The association and dissociation times were 60 s and 90 s, respectively. The binding kinetics was analyzed using Biacore insight evaluation software 4.0.8.20368 (Cytiva Inc.).

***In vivo* metastasis and drug intervention assays**

The *in vivo* assays in this study were based on a tail-vein injection model to evaluates lung colonization. Eighteen 5-week-old female BALB/c nude mice were purchased from the Laboratory Animal Center of Soochow University and housed in specific pathogen-free condition. Mice were randomly divided into three groups, termed as Vector control group, OTUD5-overexpresing group, and OTUD5-overexpresing/sh-TIF1γ group (6 mice per group). OTUD5-overexpresing, OTUD5-overexpresing/sh-TIF1γ, and control A549 cells were intravenously (i.v.) injected into murine tail vein (3×10^6 cells/mouse), respectively. TGF-β1 (4 μg/kg body weight) was intraperitoneally (i.p.) injected four times every 5 days post-inoculation to facilitate tumor cell metastasis [15]. For *in vivo* drug intervention experiments, BALB/c nude mice were divided into four groups, including sh-OTUD5 control group and OTUD5-silenced group (8 mice per group), which were injected i.p. with 4 μg/kg TGF-β1 every 5 days and/or 20 mg/kg nilotinib in saline every other day. During nilotinib treatment, the body weight and routine conditions of the mice were monitored. No systemic toxicity was seen in any of the treatment groups. Measurements were performed in a single-blinded manner. Eight weeks later, all the mice were euthanized and their lungs were resected and fixed in Bouin's solution for counting macroscopically the numbers of metastatic nodules. Lung tissues were histologically examined with H&E staining for micrometastatic foci

analysis. The animal studies were approved and supervised by the Animal Ethics Committee of Soochow University (Ethics Approval No. 202505A1080).

Establishment of SMAD3 and SMAD4 knockout stable cells

CRISPR/Cas9 system was utilized to silence SMAD3 and SMAD4 in A549 cells, which was completely described in our previous study [4].

Communication pathway analysis

To probe nilotinib's allosteric effects on OTUD5, we performed residue interaction networks (RINs) analysis using MD-TASK [16]. Networks were built from molecular dynamics (MD) trajectories with Cα (and Cβ for side-chain residues) as nodes; edges were assigned if interatomic distances stayed within 6.5 Å. Betweenness centrality (BC) was calculated to identify key communication residues. Allosteric pathways were mapped by computing weighted shortest paths (Dijkstra's algorithm) between the nilotinib-binding pocket in the OTU domain and the UIM domain [17].

Statistical analysis

Unpaired *t*-test (two-tailed) was applied for comparisons between two groups of *in vitro* and *in vivo* data, and NSCLC samples. Data were presented as mean ± SD. Sample size estimation was guided by prior publications employing comparable experimental paradigms and reporting reproducible biological effects. $P < 0.05$ were considered statistically significant. Statistical analyses were performed with GraphPad Prism 7.01 software (GraphPad).

References

1 Su Z, Sun Z, Wang Z, Wang S, Wang Y, Jin E *et al*. TIF1gamma inhibits lung adenocarcinoma EMT and metastasis by interacting with the TAF15/TBP complex. Cell Rep 2022; 41: 111513.

2 Yang H, Wang L, Zhao J, Chen Y, Lei Z, Liu X *et al*. TGF-beta-activated SMAD3/4 complex transcriptionally upregulates N-cadherin expression in non-small cell lung cancer. Lung Cancer 2015; 87: 249-257.

3 Jin E, Wang S, Chen D, Wang JP, Zeng Y, Sun R *et al*. P4HA2 activates mTOR via hydroxylation and targeting P4HA2-mTOR inhibits lung adenocarcinoma cell growth. Oncogene 2024; 43: 1813-1823.

4 Tong X, Wang S, Lei Z, Li C, Zhang C, Su Z *et al*. MYOCD and SMAD3/SMAD4 form a positive feedback loop and drive TGF-beta-induced epithelial-mesenchymal transition in non-small cell lung cancer. Oncogene 2020; 39: 2890-2904.

5 Wang L, Yang H, Lei Z, Zhao J, Chen Y, Chen P *et al*. Repression of TIF1gamma by SOX2 promotes TGF-beta-induced epithelial-mesenchymal transition in non-small-cell lung cancer. Oncogene 2016; 35: 867-877.
6 Wang S, Tong X, Li C, Jin E, Su Z, Sun Z *et al*. Quaking 5 suppresses TGF-beta-induced EMT and cell invasion in lung adenocarcinoma. EMBO Rep 2021; 22: e52079.
7 Mirdita M, Schutze K, Moriwaki Y, Heo L, Ovchinnikov S, Steinegger M. ColabFold: making protein folding accessible to all. Nat Methods 2022; 19: 679-682.
8 Jumper J, Evans R, Pritzel A, Green T, Figurnov M, Ronneberger O *et al*. Highly accurate protein structure prediction with AlphaFold. Nature 2021; 596: 583-589.
9 Varadi M, Anyango S, Deshpande M, Nair S, Natassia C, Yordanova G *et al*. AlphaFold Protein Structure Database: massively expanding the structural coverage of protein-sequence space with high-accuracy models. Nucleic Acids Res 2022; 50: D439-D444.
10 Schmidtke P, Le Guilloux V, Maupetit J, Tuffery P. fpocket: online tools for protein ensemble pocket detection and tracking. Nucleic Acids Res 2010; 38: W582-589.
11 Trott O, Olson AJ. AutoDock Vina: improving the speed and accuracy of docking with a new scoring function, efficient optimization, and multithreading. J Comput Chem 2010; 31: 455-461.
12 Hess B, Kutzner C, van der Spoel D, Lindahl E. GROMACS 4: Algorithms for Highly Efficient, Load-Balanced, and Scalable Molecular Simulation. J Chem Theory Comput 2008; 4: 435-447.
13 Wang J, Wolf RM, Caldwell JW, Kollman PA, Case DA. Development and testing of a general amber force field. J Comput Chem 2004; 25: 1157-1174.
14 Hou T, Wang J, Li Y, Wang W. Assessing the performance of the molecular mechanics/Poisson Boltzmann surface area and molecular mechanics/generalized Born surface area methods. II. The accuracy of ranking poses generated from docking. J Comput Chem 2011; 32: 866-877.
15 Marwitz S, Depner S, Dvornikov D, Merkle R, Szczygiel M, Muller-Decker K *et al*. Downregulation of the TGFbeta Pseudoreceptor BAMBI in Non-Small Cell Lung Cancer Enhances TGFbeta Signaling and Invasion. Cancer Res 2016; 76: 3785-3801.
16 Brown DK, Penkler DL, Sheik Amamuddy O, Ross C, Atilgan AR, Atilgan C *et al*. MD-TASK: a software suite for analyzing molecular dynamics trajectories. Bioinformatics 2017; 33: 2768-2771.
17 Sethi A, Eargle J, Black AA, Luthey-Schulten Z. Dynamical networks in tRNA:protein complexes. Proc Natl Acad Sci U S A 2009; 106: 6620-6625.
