# Supplementary material for: OTUD5-TIF1γ-SMAD3/4 positive feedback loop inhibits TGF-β-induced EMT and metastasis in NSCLC
Source: Cell Death Dis. 2026 May 25;17(1):650. doi: 10.1038/s41419-026-08901-z (PMC13385805; doi:10.1038/s41419-026-08901-z)
Supplement: Supplementary file 2 — Supplementary Tables S1-S5 [file 41419_2026_8901_MOESM2_ESM.pdf]

Table S1. Demographic and clinical characteristics of 74 NSCLC patients and relative mRNA expression of OTUB1, OTUD5, and OTUD7A in 74 paired NSCLC tissues.

Case	Sex	Age (yrs)	TNM	Stage	Metastasis §	OTUB1 mRNA (T/N) †	OTUD5 mRNA (T/N)†	OTUD7A mRNA (T/N)†
1	Male	70	T2N1M0	IIB	Yes	0.3035	0.5035	1.4661
2	Female	54	T1N0M0	IA	No	2.7895	0.2872	0.4061
3	Female	65	T3N2M0	IIIB	Yes	0.5249	0.4383	0.1518
4	Male	73	T1N0M0	IA	No	0.3231	2.0849	0.3737
5	Male	57	T2N1M0	IIB	Yes	1.1173	0.6285	0.1066
6	Male	65	T2N1M0	IIB	Yes	1.4896	0.8066	0.6600
7	Male	64	T1N0M0	IA	No	1.6865	1.5801	0.0249
8	Male	60	T2N1M0	IIB	Yes	1.0353	1.8025	0.6277
9	Male	64	T2N0M0	IB	No	1.0497	0.9138	1.3061
10	Female	71	T1N0M0	IA	No	1.6634	4.3770	4.2575
11	Male	62	T1N0M0	IA	No	1.8376	0.0802	0.4444
12	Male	65	T2N1M0	IIB	Yes	1.7053	0.5105	3.3223
13	Male	77	T1N2M0	IIIA	Yes	0.4007	0.4323	0.0038
14	Male	72	T2N2M0	IIIA	Yes	2.5508	0.9593	1.1500
15	Male	73	T1N0M0	IA	No	1.9325	2.4967	0.0034
16	Female	72	T2N0M0	IB	No	2.2191	0.8351	0.0140
17	Male	58	T3N0M0	IIB	No	0.5510	0.4796	0.0306
18	Male	72	T2N2M0	IIIA	Yes	1.7232	0.0508	0.0769
19	Male	85	T3N0M0	IIB	No	1.8790	0.5664	2.3457
20	Male	64	T2N2M0	IIIA	Yes	0.8950	1.2311	6.5432
21	Male	55	T1N2M0	IIIA	Yes	0.7371	0.5510	1.5157
22	Male	62	T1N1M0	IIB	Yes	2.3950	0.1708	0.9794
23	Male	57	T2N1M0	IIB	Yes	1.2658	0.1207	0.0344
24	Male	53	T1N0M0	IA	No	0.5548	0.1582	0.1199
25	Male	62	T2N2M0	IIIA	Yes	2.0219	0.0367	2.4453
26	Male	75	T2N2M0	IIIA	Yes	1.1404	0.0961	0.0029
27	Male	72	T2N0M0	IB	No	1.2058	4.8569	1.2616
28	Male	66	T2N0M0	IB	No	2.9690	1.2570	0.0369
29	Male	60	T2N0M0	IB	No	0.1497	0.6329	0.7113
30	Male	62	T2N2M0	IIIA	Yes	1.5777	0.7422	0.5946
31	Female	67	T1N0M0	IA	No	0.2994	0.5824	0.0296
32	Male	65	T1N0M0	IA	No	1.0312	0.2059	0.3167
33	Male	61	T2N2M0	IIIA	Yes	3.3173	1.8025	1.4142
34	Male	67	T2N0M0	IB	No	3.0223	1.9588	0.4785
35	Female	40	T1N1M0	IIB	Yes	1.4845	0.8586	2.6574
36	Female	52	T2N0M0	IB	No	0.2141	0.2192	0.3143
37	Male	74	T2N2M0	IIIA	Yes	2.1264	0.5625	0.0836
38	Female	71	T2N0M0	IB	No	0.7945	2.4453	1.9814
39	Male	67	T2N0M0	IIB	No	5.7757	0.8066	0.8716
40	Male	65	T1N0M0	IA	No	1.0930	0.9202	0.7965
41	Male	73	T1N0M0	IA	No	2.9690	1.0644	1.2005

42	Male	65	T2N2M0	IIIA	Yes	1.2514	1.3013	0.1066
43	Male	58	T2N1M0	IIB	Yes	1.1975	1.3195	0.1650
44	Male	68	T4N0M0	IIIA	No	2.7478	0.4966	0.0347
45	Female	67	T3N0M0	IIB	No	0.5827	1.5475	0.6962
46	Male	60	T2N2M0	IIIA	Yes	0.2900	0.6507	0.7715
47	Female	57	T2N0M0	IB	No	0.8179	1.0718	0.3896
48	Female	64	T4N2M1	IV	Yes	5.3889	1.1975	0.1539
49	Male	68	T1N1M0	IIB	Yes	0.6552	0.2466	0.7371
50	Female	56	T2N2M0	IIIA	Yes	0.3121	1.2058	0.4204
51	Male	75	T2N0M0	IIA	No	1.2834	0.1250	0.3789
52	Female	69	T2N0M0	IB	No	0.2852	0.2606	0.5743
53	Female	42	T2N0M0	IB	No	1.0140	0.9659	0.2136
54	Female	61	T2N1M0	IIA	Yes	1.1926	1.2397	0.2994
55	Male	73	T3N2M0	IIIB	Yes	3.1383	1.7052	1.6245
56	Male	71	T2N0M0	IIA	No	1.8661	1.4340	1.5453
57	Male	65	T2N0M0	IIA	No	0.4005	0.7071	0.3978
58	Male	77	T3N0M0	IIB	No	1.6245	3.2043	0.0754
59	Male	68	T4N2M0	IIIB	Yes	0.3253	0.2432	0.1768
60	Male	58	T3N0M0	IIB	No	1.0210	0.9330	0.0629
61	Male	73	T3N0M0	IIB	No	0.3536	1.1251	0.0890
62	Female	71	T2N0M0	IB	No	1.3672	0.8766	0.3231
63	Male	47	T3N2M0	IIIB	Yes	2.2666	0.5864	0.2670
64	Female	63	T4N0M1	IV	Yes	3.0843	0.0961	0.0304
65	Female	59	T2N2M0	IIIA	Yes	0.8526	1.0792	4.5948
66	Female	57	T2N2M0	IIIA	Yes	1.6133	0.0733	0.1073
67	Female	64	T4N2M0	IIIB	Yes	1.5476	0.4061	0.0775
68	Male	68	T2N2M0	IIIA	Yes	0.6199	0.9330	0.3737
69	Male	69	T4N2M1	IV	Yes	1.2570	1.3947	0.6878
70	Male	87	T2N0M0	IIA	No	2.2346	2.8679	0.5212
71	Female	64	T1N0M0	IA	No	0.5664	0.2912	0.0878
72	Male	73	T3N0M0	IIB	No	1.0943	0.7526	0.6373
73	Male	54	T4N1M0	IIIA	Yes	1.5483	0.4293	0.3737
74	Female	65	T2N1M0	IIB	Yes	0.3299	2.1886	0.7274

[§] Metastasis, local lymph node metastasis or distant organ metastasis.

[†] T, tumor tissues; N, matched adjacent noncancerous tissues.

Table S2. Mass spectrometry-based analysis of the proteins interacting with OTUD5 in 293T cells.

Accession	Gene name	Coverage (%)	Peptides	Unique Peptides	Score	MW (kDa)
Q96G74	OTUD5	51	29	29	382.13	60.6
Q9UJV9	DDX41	72	42	42	221.03	69.8
A0A0G2JIW1	HSPA1B	53	29	17	173.44	70.1
A0A0J9YVP6	PUF60	58	25	25	153.58	57.4
P49756	RBM25	37	33	33	116.38	100.1
O43143	DHX15	35	27	26	114.31	90.9
H6VRF8	KRT1	45	28	23	108.48	66.0
O95071	UBR5	17	41	41	101.88	309.2
O75533	SF3B1	33	34	34	99.85	145.7
Q9Y5B9	SUPT16H	31	31	31	98.94	119.8
Q15393	SF3B3	27	28	28	93.18	135.5
Q8IZ29	TUBB2C	60	20	4	85.03	49.8
V9HWB4	HEL-S-89n	40	23	21	79.96	72.3
P35908	KRT2	52	26	19	79.14	65.4
E9PK54	HSPA8	89	13	9	78.78	19.9
Q15365	PCBP1	57	13	9	72.30	37.5
P13639	EEF2	26	22	21	71.38	95.3
Q9BY77	POLDIP3	47	16	10	69.13	46.1
A0A1B0GVI3	KRT10	35	20	17	68.42	63.3
P27708	CAD	16	34	34	66.15	242.8
P54652	HSPA2	23	13	4	65.41	70.0
Q8N163	CCAR2	30	23	18	64.84	102.8
Q06830	PRDX1	67	12	11	64.40	22.1
O00571	DDX3X	31	19	19	63.57	73.2
P17812	CTPS1	35	20	18	61.93	66.6
P35527	KRT9	39	19	18	60.36	62.0
F5H5D3	TUBA1C	44	17	1	58.09	57.7
A0A0S2Z4Z0	RBM14	29	19	19	55.25	69.4
A0A5H1ZRQ2	DDX17	24	18	11	50.61	80.4
P38919	EIF4A3	32	17	15	49.94	46.8
A0A024R1K8	SF3A1	30	21	21	49.16	88.8
A0A7I2V599	HSPD1	28	13	13	48.68	57.8
F8VZX2	PCBP2	39	9	5	45.39	33.8
P21127	CDK11B	26	21	21	44.69	92.6
A0A0C4DG89	DDX46	23	21	21	43.86	117.4
Q9H5Z1	DHX35	39	24	24	43.46	78.9
Q6PJT7	ZC3H14	25	15	15	42.97	82.8
O43290	SART1	25	16	16	42.36	90.2
Q9NRF8	CTPS2	24	13	11	41.99	65.6
B5BUE6	DDX5	30	18	11	41.83	69.1
Q2TAY7	SMU1	27	11	11	40.13	57.5
Q13769	THOC5	27	18	18	39.11	78.5
Q8WUT1	POLDIP3	48	7	1	39.09	24.8

Q13435	SF3B2	22	19	19	38.78	100.2
A0A024R0F6	TRIM33	10	11	11	37.86	122.4
X6R700	CHTOP	32	6	6	37.79	23.6
Q8IX18	DHX40	24	18	18	36.59	88.5
Q96AG4	LRRC59	26	7	7	35.42	34.9
A0A024RDU9	GTF2F2	48	11	11	33.66	28.4
P13647	KRT5	24	15	9	33.64	62.3
A0A024RDQ0	HSPH1	16	11	10	33.40	92.1
A0A0S2Z428	KRT6A	28	15	1	32.99	60.0
Q9Y5B6	PAXBP1	16	15	15	32.92	104.7
E9PB61	ALYREF	32	5	5	32.73	27.5
P02533	KRT14	27	12	3	32.66	51.5
J3QTR3	RPS27A	41	4	4	32.39	12.2
P05141	SLC25A5	37	12	3	31.64	32.8
P08779	KRT16	22	10	1	31.25	51.2
Q8NE71	ABCF1	18	13	13	30.63	95.9
A0A8I5KV60	TAF2	12	15	15	29.85	142.6
A0A024RAC5	RCC2	25	11	11	29.59	56.0
A0A024RD80	HSP90AB1	19	12	7	29.13	83.2
Q96I25	RBM17	33	12	12	28.78	44.9
K9JA46	EL52	15	10	5	28.55	84.6
Q9BRX9	WDR83	27	8	8	28.55	34.3
Q8N523	TFIP11	16	11	11	27.98	96.7
O14617	AP3D1	13	13	13	27.91	130.1
Q93009	USP7	11	12	12	27.72	128.2
Q96IR1	RPS4X	36	10	10	27.04	27.2
F8VTV8	CDK4	45	9	8	26.94	22.0
O75643	SNRNP200	9	18	18	26.86	244.4
Q15051	IQCB1	20	13	13	26.52	68.9
Q96FV9	THOC1	23	15	15	26.34	75.6
K7EJV9	RPL23A	26	7	7	26.29	19.4
Q6I9V5	SLC25A6	34	10	1	26.13	32.8
A0A499FJY3	RBM15	17	16	16	26.12	101.3
P31943	HNRNPH1	30	9	7	25.68	49.2
Q13838	DDX39B	26	10	10	25.11	49.0
Q6UN15	FIP1L1	18	8	8	24.17	66.5
V9HWC6	HEL-S-39	42	9	9	23.74	22.7
P62829	RPL23	62	7	7	22.99	14.9
O94906	PRPF6	11	10	10	22.80	106.9
Q86W42	THOC6	32	8	8	22.71	37.5
A0A024R566	SF1	15	6	1	22.47	61.9
Q9Y3I0	RTCB	13	6	6	22.18	55.2
P22061	PCMT1	45	9	9	22.07	24.6
Q96QD9	FYTTD1	33	10	10	22.04	35.8
Q8NI27	THOC2	8	14	14	21.65	182.7

E5RFJ3	CCAR2	46	6	1	21.54	17.5
Q92945	KHSRP	11	6	6	21.50	73.1
A0A024R8R0	SAP30BP	19	7	7	21.34	33.9
B5BU25	U2AF2	17	6	6	20.50	53.1
A0A0S2Z3H3	SLC25A4	31	9	1	20.31	33.0
P62937	PPIA	48	7	7	20.18	18.0
O60841	EIF5B	10	11	11	19.86	138.7
Q8IWX8	CHERP	9	8	8	19.83	103.6
P62280	RPS11	33	5	5	19.65	18.4
P07355	ANXA2	25	7	7	19.45	38.6
Q8WUA2	PPIL4	21	11	11	19.39	57.2
Q04695	KRT17	11	5	1	19.27	48.1
P62917	RPL8	16	4	4	19.04	28.0
Q14240	EIF4A2	19	8	2	18.89	46.4
Q53H96	PYCR3	33	7	7	18.87	28.6
Q9Y2W1	THRAP3	9	8	7	18.79	108.6
A0A7I2V4F0	DDX1	17	11	11	18.31	81.7
Q14554	PDIA5	18	8	8	18.02	59.6
Q9UMS4	PRPF19	19	7	7	17.66	55.1
O14744	PRMT5	11	8	8	17.19	72.6
Q14331	FRG1	28	6	6	16.98	29.2
H0Y8C6	IPO5	11	10	10	16.95	123.8
Q8N684	CPSF7	16	7	7	16.79	52.0
P46109	CRKL	30	7	7	16.57	33.8
Q5T7C4	HMGB1	32	5	4	16.50	18.3
P10599	TXN	31	3	3	16.38	11.7
Q15182	SNRPB	14	5	5	16.21	29.7
P42766	RPL35	16	3	3	16.18	14.5
O43684	BUB3	14	4	4	15.92	37.1
Q96HS1	PGAM5	19	7	7	15.67	32.0
A0A7I2V5M5	NCL	10	8	8	15.64	73.6
Q96BS4	FBL	31	6	6	15.31	28.4
A0A384ME17	TUFM	23	9	9	14.93	49.8
P55081	MFAP1	18	5	5	14.90	51.9
A0A024RCR6	BAG6	9	9	9	14.75	118.6
P41091	EIF2S3	14	6	6	14.41	51.1
Q01081	U2AF1	27	4	3	14.31	27.9
P27348	YWHAQ	26	6	4	13.97	27.7
Q53F19	NCBP3	12	7	7	13.90	70.5
Q1HBJ4	MAPK1	19	8	6	13.66	41.4
Q86YB2	DHX8	5	7	6	13.66	139.2
Q9UGI8	TES	18	8	8	13.39	48.0
V9HW01	HEL-S-310	25	4	4	13.15	17.8
E9PK25	CFL1	18	4	4	12.92	22.7
Q9BZI7	UPF3B	21	8	8	12.77	57.7

P62851	RPS25	28	4	4	12.63	13.7
O14654	IRS4	8	9	9	12.55	133.7
Q6P2Q9	PRPF8	5	13	13	12.46	273.4
O75131	CPNE3	13	7	7	12.45	60.1
P09012	SNRPA	16	3	1	12.25	31.3
B5BUB1	RUVBL1	10	4	4	12.19	50.2
Q9Y383	LUC7L2	19	7	7	12.09	46.5
Q3YEC7	RABL6	12	8	8	12.07	79.5
P0DMU9	CT45A10	22	4	4	11.94	21.1
P62318	SNRPD3	32	3	3	11.94	13.9
Q9NYF8	BCLAF1	7	6	5	11.79	106.1
Q10570	CPSF1	4	6	6	11.70	160.8
Q5QNW6	H2BC18	29	5	2	11.66	13.9
Q9BVP2	GNL3	10	6	6	11.59	62.0
P36578	RPL4	10	4	4	11.58	47.7
P63173	RPL38	39	3	3	11.58	8.2
A0A087X0X3	HNRNPM	6	5	5	11.52	77.5
P61106	RAB14	30	6	6	11.45	23.9
A0A6I8PU73	DNAJC7	12	6	6	11.42	56.7
G3V207	TMCC3	19	8	8	11.42	50.1
Q96SB4	SRPK1	18	10	10	11.23	74.3
Q5RI18	HNRNPU	9	5	5	10.95	67.8
Q8ND56	LSM14A	15	7	7	10.92	50.5
Q9Y305	ACOT9	24	10	10	10.90	49.9
Q9NR30	DDX21	12	8	6	10.71	87.3
G9K389	YWHAE/FAM22B	10	4	2	10.65	41.3
Q96J01	THOC3	16	6	6	10.64	38.7
K7ERP4	GPX4	17	3	3	10.63	17.6
B1ALA9	PRPS1	20	5	5	10.62	31.4
H7C561	SF1	12	3	1	10.58	30.1
A0A024QZD5	SNRP70	12	6	6	10.54	51.5
Q9NVP1	DDX18	7	5	5	10.44	75.4
Q09028	RBBP4	12	5	5	10.28	47.6
H7C3A1	SRRT	8	4	4	10.21	56.7
P23396	RPS3	28	6	6	10.07	26.7
Q14244	MAP7	9	6	6	9.98	84.0
Q9Y230	RUVBL2	12	5	5	9.97	51.1
B4DY08	HNRNPC	11	4	4	9.84	32.0
Q9Y2T2	AP3M1	20	6	6	9.72	46.9
B2R4R0	HIST1H4J	39	4	4	9.67	11.4
P23526	AHCY	9	4	4	9.62	47.7
O75909	CCNK	8	3	3	9.58	64.2
Q53XC0	EIF2S1	30	9	9	9.47	36.1
A0A024R6H0	CPSF2	5	4	4	9.40	88.4
P52597	HNRNPF	14	4	2	9.35	45.6

O43175	PHGDH	8	4	4	9.33	56.6
A0A024R1A4	UBE2L3	47	5	5	9.31	17.9
Q9Y6A4	CFAP20	21	4	4	9.30	22.8
B5BU01	EIF2S2	9	3	3	9.29	38.3
Q9BSV4	SFPQ	12	7	7	9.28	68.6
Q4VB24	HIST1H1E	15	3	3	9.10	21.9
B5MCF9	PES1	15	6	6	8.96	66.0
P62891	RPL39	20	1	1	8.89	6.4
I3L397	EIF5A	21	4	4	8.69	16.0
A0A0D9SGE8	PHF6	17	6	6	8.63	41.3
Q2TBE0	CWF19L2	15	12	12	8.56	103.7
A0A2R8YDP2	CSNK2A1	17	5	5	8.41	40.6
P62424	RPL7A	15	5	5	8.23	30.0
Q9UNE7	STUB1	9	3	3	8.14	34.8
D0PNI1	YWHAZ	12	3	1	8.02	27.7
Q15007	WTAP	9	3	3	8.02	44.2
A0A024RBB5	CSRP2	24	3	3	8.00	20.9
A0A024RAS3	hCG_27698	7	3	3	7.98	50.6
F8WJN3	CPSF6	13	5	5	7.92	52.2
A0A2R8Y811	RPS14	23	3	3	7.75	16.1
E9PAV9	GPATCH4	14	4	4	7.73	42.6
J3KNF4	CCS	11	3	3	7.68	27.1
J3KRX5	RPL17	22	3	3	7.63	20.2
A0A6Q8PG37	KIF2A	10	8	8	7.61	81.0
P47813	EIF1AX	18	3	3	7.59	16.5
A0A8V8TM61	PRPF40A	4	4	4	7.55	106.3
C9JYQ9	RPL22L1	26	3	3	7.51	14.5
Q6P1J9	CDC73	10	6	6	7.50	60.5
P61326	MAGOH	24	3	3	7.41	17.2
Q8IWV8	UBR2	7	11	11	7.41	200.4
Q02978	SLC25A11	16	4	4	7.37	34.0
Q8N5L9	RPS2	14	4	4	7.34	31.3
Q9HCM4	EPB41L5	6	4	4	7.31	81.8
Q9UKV3	ACIN1	4	4	4	7.26	151.8
P31689	DNAJA1	9	3	3	7.19	44.8
K7PML8	CDKN2A	19	2	2	7.17	16.5
P62277	RPS13	16	3	3	7.09	17.2
Q4KMP7	TBC1D10B	10	6	6	7.09	87.1
F8W7C6	RPL10	25	4	4	7.07	18.6
Q16778	H2BC21	29	4	1	7.04	13.9
A0A024R4M0	RPS9	20	5	5	6.82	22.6
F8WCT1	ARL6IP4	8	1	1	6.82	25.6
A0A024R1T5	CNP	8	3	3	6.79	45.1
G3V5V3	NEMF	3	3	3	6.78	113.9
Q969Q0	RPL36AL	42	5	5	6.73	12.5

Q16777	H2AC20	27	3	3	6.72	14.0
P81605	DCD	20	2	2	6.65	11.3
Q9Y3B4	SF3B6	22	3	3	6.61	14.6
P62314	SNRPD1	20	3	3	6.58	13.3
A0A7P0Z3Z5	DDB1	7	6	6	6.50	119.0
A0A7I2V5Y3	BTF3	26	2	2	6.48	10.7
P26373	RPL13	15	3	3	6.34	24.2
Q549M8	C14orf166	14	3	3	6.34	28.1
P06493	CDK1	6	2	1	6.31	34.1
C9J384	CMSS1	19	4	4	6.27	26.2
P08579	SNRPB2	11	3	1	6.23	25.5
Q08945	SSRP1	9	7	7	6.16	81.0
A0A3B3ITJ4	HNRNPL	8	4	4	6.12	59.2
J3KPD9	NME1-NME2	30	4	4	6.08	22.4
A0A024R1Z1	CDC34	16	4	4	6.07	26.7
Q7RTV0	PHF5A	22	3	3	6.05	12.4
A0A7I2V535	NONO	6	3	3	6.04	57.6
B9EG90	TOP1	3	3	3	6.04	90.6
A0MNP2	WDR57	9	3	3	5.96	39.3
Q6PJJ2	RRP1	10	4	4	5.94	52.7
Q9BU76	MMTAG2	16	4	4	5.94	29.4
H7C2W9	RPL31	35	4	4	5.93	12.8
Q1L5Z9	LONRF2	4	4	4	5.92	83.6
P52292	KPNA2	8	5	5	5.79	57.8
A0A0S2Z4T1	MCM3	4	4	4	5.78	90.9
Q9H5V9	STEEP1	18	4	4	5.75	25.6
Q9UKJ3	GPATCH8	3	4	4	5.75	164.1
Q9NY12	GAR1	21	5	5	5.74	22.3
I4AY87	MIF	10	1	1	5.73	12.5
Q8NC51	SERBP1	11	5	5	5.71	44.9
Q8TDD1	DDX54	5	5	4	5.70	98.5
A0A024R8D7	LCN1	13	2	2	5.68	19.2
P05109	S100A8	24	2	2	5.64	10.8
A1A4E9	KRT13	9	4	1	5.60	49.6
A0A6I8PRA0	TCOF1	8	9	9	5.56	125.2
Q9BRP1	PDCD2L	10	3	3	5.51	39.4
A0A024R8A7	hCG_31253	3	2	2	5.46	61.6
P35080	PFN2	16	2	1	5.43	15.0
A0A0B4J259	LYZ	9	1	1	5.41	15.3
Q8TBR3	FUS	9	5	5	5.28	53.4
Q16763	UBE2S	10	2	2	5.27	23.8
P35520	CBS	9	5	5	5.25	60.5
A0A024R179	NCBP1	8	6	6	5.24	91.8
P21741	MDK	12	2	2	5.23	15.6
O15371	EIF3D	6	3	3	5.19	63.9

O95470	SGPL1	2	2	2	5.13	63.5
A6QKW0	SHINC3	8	2	2	5.11	27.9
P38159	RBMX	13	5	5	5.10	42.3
D6RD47	RPS23	17	2	2	4.97	14.8
P50990	CCT8	4	2	2	4.93	59.6
Q9BQQ5	L27a	27	3	3	4.91	12.0
P62316	SNRPD2	16	2	2	4.85	13.5
Q8N5A5	ZGPAT	8	4	4	4.84	57.3
B5MD38	HADHB	7	2	2	4.75	37.9
A0A087WTT1	PABPC1	9	5	5	4.73	65.2
Q6I9Y2	THOC7	22	4	4	4.73	23.7
Q8N1G4	LRRC47	5	3	3	4.72	63.4
P26641	EEF1G	12	5	5	4.66	50.1
H0Y368	DPM1	7	2	2	4.65	33.3
O95391	SLU7	7	4	4	4.63	68.3
H0Y449	YBX1	14	3	3	4.62	42.0
P36957	DLST	5	2	2	4.57	48.7
P62269	RPS18	18	3	3	4.54	17.7
P26583	HMGB2	20	3	2	4.51	24.0
A0A7I2YQG2	RPL19	4	1	1	4.49	25.7
Q15717	ELAVL1	6	2	2	4.49	36.1
Q5VVC8	RPL11	18	3	3	4.48	19.0
P24941	CDK2	6	2	1	4.44	33.9
H0Y8X0	DEK	21	6	6	4.43	32.7
B0QYK0	EWSR1	6	3	3	4.42	64.9
Q8N0V3	RBFA	6	2	2	4.40	38.3
P51570	GALK1	8	3	3	4.36	42.2
Q9BYG3	NIFK	19	4	4	4.36	34.2
B7Z7B0	CPSF4	16	2	2	4.35	21.9
P61513	RPL37A	20	1	1	4.35	10.3
P37108	SRP14	30	4	4	4.34	14.6
A0A8I5QKQ5	PDIA3	4	2	2	4.33	51.0
H0YLI7	AP3S2	10	2	2	4.32	23.4
V9HW96	HEL-S-100n	4	2	2	4.31	57.5
Q96H79	ZC3HAV1L	7	2	2	4.29	32.9
Q9Y5T5	USP16	1	1	1	4.29	93.5
A0A2R8Y6G6	ENO1	7	3	3	4.26	47.3
Q9NVI7	ATAD3A	4	3	3	4.26	71.3
A0A024R0Z3	DDX23	5	4	4	4.23	95.5
P42167	TMPO	11	4	4	4.23	50.6
P62906	RPL10A	13	3	3	4.23	24.8
A0A6I8PS19	GNL3L	6	3	3	4.18	58.0
E4W6B6	RPL27	21	3	3	4.12	14.2
B4DY09	ILF2	11	3	3	4.07	38.9
Q5JR94	RPS8	19	4	4	4.03	24.2

A0A024R7L5	UPF1	3	3	3	4.02	123.0
M0R0R2	RPS5	4	1	1	4.02	25.3
Q3B7A7	GART	2	2	2	4.02	107.7
H0Y9X1	TMA16	24	6	6	4.01	27.7
A0A024RB14	RPS26	21	2	2	3.95	13.0
V9HWC7	HEL-S-128m	13	4	4	3.93	25.0
A0A024RCA6	SLC25A22	6	2	2	3.90	26.6
Q07666	KHDRBS1	11	4	4	3.88	48.2
B8ZZR0	CLK1	5	2	2	3.87	36.2
Q7Z3H5	DKFZp686O0215	11	4	1	3.83	37.5
P49207	RPL34	21	3	3	3.75	13.3
Q9BTI9	NPM1	6	2	2	3.73	25.0
Q96BR5	COA7	8	2	2	3.68	25.7
E9PDF6	MYO1B	2	2	2	3.56	128.4
A0A8I5KVM3	TAF8	8	2	2	3.51	35.1
Q86YZ3	HRNR	4	2	2	3.51	282.2
A6NLN1	PTBP1	10	5	5	3.49	56.5
A0A024QZ42	PDCD6	9	1	1	3.45	14.4
K7EL20	EIF3G	8	2	2	3.43	29.3
A0A024R0H7	WDR77	17	4	4	3.39	36.7
B4DT23	FAM98A	8	2	2	3.25	34.1
M0R210	RPS16	22	3	3	3.23	14.4
O60884	DNAJA2	4	2	2	3.22	45.7
E7EUT5	GAPDH	12	3	3	3.18	27.9
A0A024QZR3	hCG_2002731	7	3	3	3.08	43.4
Q53XM7	VAPB	5	1	1	3.04	27.2
Q5T760	SRSF11	3	1	1	3.03	54.3
Q9Y2X3	NOP58	5	2	2	3.03	59.5
N0E4C7	CSNK2B	12	2	2	2.86	24.9
Q8WXE9	STON2	1	1	1	2.78	101.1
A0A0S2Z4Z6	SRRM1	2	2	2	2.77	103.9
H0Y9Y4	RPS3A	20	4	4	2.77	23.5
Q9H3K6	BOLA2B	59	4	4	2.76	10.1
Q15084	PDIA6	3	1	1	2.73	48.1
S4R3G0	SRSF9	15	2	1	2.72	13.8
Q7Z666	DKFZp779I1064	4	1	1	2.70	27.7
Q2NL82	TSR1	3	2	2	2.67	91.8
E7ET15	U2SURP	4	3	3	2.66	118.2
P02452	COL1A1	1	1	1	2.66	138.8
Q96CS3	FAF2	5	2	2	2.62	52.6
Q6YN16	HSDL2	10	4	4	2.58	45.4
A2A3R5	RPS6	6	1	1	2.57	25.0
Q5TFE4	NT5DC1	3	1	1	2.56	51.8
H3BNI9	CSNK2A2	7	2	2	2.54	35.4
A0A024R326	RPL29	15	2	2	2.53	17.5

P17987	TCP1	9	5	5	2.53	60.3
P62861	FAU	8	1	1	2.50	14.4
Q5M9Q1	NKAPL	2	1	1	2.48	46.3
Q8WU68	U2AF1L4	15	2	1	2.48	25.7
P84090	ERH	11	1	1	2.47	12.3
M0R0C3	TIMM50	11	1	1	2.44	12.0
H0YIV5	LETMD1	8	1	1	2.42	16.4
Q6I9T8	PPP2CA	9	3	3	2.42	35.5
Q9Y295	DRG1	9	3	3	2.42	40.5
K7EIY6	RNF126	8	1	1	2.40	30.8
A0A024R1T9	ACLY	3	4	4	2.39	120.8
P30050	RPL12	5	1	1	2.37	17.8
P50402	EMD	11	2	2	2.37	29.0
Q6P6C2	ALKBH5	3	1	1	2.37	44.2
A0A494C128	NOP56	4	2	2	2.36	53.0
Q3B726	POLR1F	3	1	1	2.36	37.4
A0A087X2D0	SRSF3	9	1	1	2.35	10.3
B4DZG7	ARL1	7	1	1	2.31	15.4
O95801	TTC4	5	2	2	2.29	44.7
Q69YN2	CWF19L1	2	1	1	2.28	60.6
Q76LA1	CSTB	12	1	1	2.26	11.1
Q8WVV9	HNRNPLL	5	2	2	2.26	60.0
A0A024R534	MTA2	1	1	1	2.25	75.0
J3KN67	TPM3	4	1	1	2.25	33.2
Q0QEL2	CS	5	1	1	2.25	24.9
Q6IAX2	RPL21	14	2	2	2.24	18.6
Q9UKD2	MRTO4	4	1	1	2.24	27.5
Q69YP1	DKFZp762M013	3	2	2	2.23	87.3
A0A7I2YQL3	WDR26	2	1	1	2.21	65.4
A0A024R029	ABT1	7	2	2	2.20	31.1
B4DEX8	MAT2A	7	2	2	2.20	39.7
P50991	CCT4	6	3	3	2.20	57.9
Q86UK7	ZNF598	1	1	1	2.20	98.6
A0A669KB29	CXXC1	2	1	1	2.17	74.6
Q53GS9	USP39	2	1	1	2.17	65.3
Q9GZZ8	LACRT	13	2	2	2.16	14.2
C9JYI4	KPNA1	9	2	2	2.15	36.8
A0JLU5	ESF1	3	2	2	2.14	63.4
Q8TED0	UTP15	4	2	2	2.14	58.4
D6RCB9	NHP2	7	1	1	2.13	15.2
P36551	CPOX	2	1	1	2.13	50.1
Q15061	WDR43	1	1	1	2.13	74.8
Q9Y2S6	TMA7	14	1	1	2.12	7.1
Q6IBU0	EIF5	2	1	1	2.11	49.1
Q92522	H1-10	5	1	1	2.11	22.5

A5PLN4	SF4	4	2	2	2.10	72.5
Q99832	CCT7	4	3	3	2.10	59.3
A0A024R598	LOC51035	4	1	1	2.08	33.3
A0A384MDU2	COL1A2	1	1	1	2.08	129.2
Q05DF2	SF3A2	4	2	2	2.08	51.4
Q7L3T8	PARS2	2	1	1	2.08	53.2
A0A0A0MRM9	NOLC1	1	1	1	2.05	74.6
Q9Y4C2	TCAF1	2	2	2	2.04	102.1
Q12906	ILF3	3	2	2	2.03	95.3
A0A024R2L1	WDR48	3	2	2	2.01	76.2
E9PC15	AGK	2	1	1	2.01	43.8
M0R2N5	TECR	7	2	2	2.01	39.9
Q6I9T7	CBX5	6	1	1	2.01	22.2
X6RAJ1	AKAP17A	2	1	1	2.01	51.5
A0A3B3ISJ3	CAAP1	7	1	1	2.00	25.1
Q6IB11	PGRMC1	7	1	1	2.00	21.7
H7C224	IRAK1	2	1	1	1.97	42.2
P39023	RPL3	3	1	1	1.97	46.1
D3DV26	S100A10	5	1	1	1.96	22.3
P05388	RPLP0	6	2	2	1.96	34.3
P42677	RPS27	38	3	3	1.96	9.5
Q9H2R7	NPD011	4	1	1	1.95	24.3
O00629	KPNA4	2	1	1	1.93	57.9
A0A087X0M4	SLC4A1AP	1	1	1	1.92	82.8
Q6ICN0	GRB2	3	1	1	1.92	25.1
G3V4T2	PABPN1	20	4	4	1.88	20.2
Q96QK1	VPS35	1	1	1	1.88	91.6
P39019	RPS19	6	1	1	1.87	16.1
H3BLY3	CIR1	9	1	1	1.86	10.7
Q15370	ELOB	6	1	1	1.86	13.1
Q16629	SRSF7	5	1	1	1.85	27.4
O95816	BAG2	4	1	1	1.84	23.8
Q9GZP4	PITHD1	4	1	1	1.84	24.2
O00422	SAP18	14	3	3	1.83	17.6
P18124	RPL7	7	2	2	1.83	29.2
Q96GA3	LTV1	2	1	1	1.83	54.8
H0Y714	IMP4	5	2	2	1.82	31.9
H0YHA7	RPL18	13	2	2	1.82	19.0
P62081	RPS7	9	2	2	1.82	22.1
H0Y3N9	PHF8	2	2	2	1.81	116.8
Q9HB71	CACYBP	13	2	2	1.81	26.2
H0Y5S9	CSNK1E	23	2	2	1.80	13.0
A0A0S2Z556	PQBP1	4	1	1	1.79	32.2
A0A286YF97	MAPK10	2	1	1	1.79	52.3
Q00688	FKBP3	8	2	2	1.79	25.2

Q96D46	NMD3	2	1	1	1.79	57.6
A0A7I2V2S3	XPO1	2	3	3	1.78	109.3
A0A024RBK3	RPL6	11	3	3	1.77	32.7
E9PLW6	AASDHPPT	5	1	1	1.77	22.2
Q6MZS5	DKFZp686A13234	3	2	2	1.77	68.3
A0A087WYT3	PTGES3	13	2	2	1.76	19.1
B1AKM8	PISD	4	1	1	1.75	26.3
Q658X2	DKFZp666I2110	4	1	1	1.75	26.7
B5MCH7	BZW2	2	1	1	1.74	39.4
I3L303	RPS15A	17	1	1	1.74	6.0
J3KS15	MRPL58	6	1	1	1.73	21.9
P31151	S100A7	11	1	1	1.73	11.5
Q9BVI4	NOC4L	6	3	3	1.73	58.4
A0A8I5KTZ9	FMR1	1	1	1	1.72	63.1
A0A804HHW5	HSD17B10	4	1	1	1.71	22.3
A6PVX3	PSMD4	7	1	1	1.71	21.8
Q92917	GPKOW	3	2	2	1.71	52.2
O75937	DNAJC8	3	1	1	1.70	29.8
Q6UXN9	WDR82	4	2	2	1.70	35.1
Q9UHR4	BAIAP2L1	1	1	1	1.70	56.8
H7C519	NT5DC2	5	1	1	1.68	23.4
Q9BQ61	TRIR	7	1	1	1.68	18.4
A0A7I2V2U6	MTHFD2	3	1	1	1.67	35.2
P30626	SRI	6	1	1	1.67	21.7
P0DPB5	POLR1D	11	2	2	1.66	14.3
P18583	SON	0	1	1	1.66	263.7
Q13200	PSMD2	1	1	1	1.66	100.1
Q9NW64	RBM22	3	1	1	1.66	46.9
Q5T8P6	RBM26	1	2	2	1.65	113.5
Q8NFF5	FLAD1	1	1	1	1.65	65.2
Q9H0A0	NAT10	2	2	2	1.65	115.7
Q9Y3C6	PPIL1	4	1	1	1.65	18.2
Q7L0Y3	TRMT10C	5	2	2	1.64	47.3
Q8N516	IKBKAP	1	1	1	1.64	150.1
A0A024R254	MAGED1	2	2	2	1.63	86.1
A0A0S2Z5U7	DIABLO	9	2	2	1.63	27.1
A0A6I8PRS5	GPATCH11	3	1	1	1.63	30.7
B1AJY5	PSMD10	12	2	2	1.63	20.2
C9JRD2	DNAJB2	4	1	1	1.63	25.4
I3L2C6	RHOT2	4	1	1	1.63	23.7
Q86X55	CARM1	2	2	2	1.62	65.8
A0A384MR33	SMC1L1	1	1	1	1.61	143.1
Q00587	CDC42EP1	2	1	1	1.61	40.3
Q6P1M0	SLC27A4	1	1	1	1.61	72.0

Table S3. Virtual screening of candidate pharmaceutical compounds targeting OTUD5.

ZINC ID	Docking score	Name	Therapeutic indications	Mechanisms of action (MoA)
**ZINC000003934128**	-10.7	Temoporfin	Head and neck squamous cell carcinoma	Photosensitizing agent
**ZINC000006716957**	-10.7	Nilotinib	Chronic myeloid leukemia	Abl kinase inhibitor, Bcr-Abl kinase inhibitor
**ZINC000011677911**	-10.4	Bisoctrizole	Urticaria	Absorber of UVA and UVB radiation
**ZINC000885764928**	-10.3	Paritaprevir	Hepatitis C Virus (HCV)	Antiviral agent
**ZINC000169621219**	-10	Eribulin	Metastatic breast cancer and metastatic or unresectable liposarcoma	Microtubule inhibitor
**ZINC000150338819**	-9.9	Ledipasvir	Hepatitis C virus	Antiviral agent
**ZINC000164760756**	-9.9	Olysio	Hepatitis C virus	Antiviral agent
**ZINC000000896717**	-9.8	Accolate	Asthma, Chronic urticaria	Leukotriene receptor antagonist
**ZINC000095862733**	-9.7	Digitoxin	Congestive cardiac insufficiency, Arrhythmias and Heart failure	Cardiac glycoside
**ZINC000100013130**	-9.7	Midostaurin	High-risk acute myeloid leukemia (AML) with specific mutations, Aggressive systemic mastocytosis (ASM), Systemic mastocytosis with associated hematologic neoplasm (SM-AHN), or Mast cell leukemia (MCL)	Antineoplastic agent, FLT3 inhibitor, KIT inhibitor, PKC inhibitor

Table S4. Primers used for plasmid construction, qRT-PCR, and ChIP, as well as sequences of shRNAs and siRNAs.

**Primers for plasmid construction**	**Sequence, 5' – 3'**
HA-OTUD5-1-F	GGGAGACCCAAGCTGGCTAGC
HA-OTUD5-1-R	AACATCGTATGGGTAACCGGTCTCCTCACGCTCGGGACTG
HA-OTUD5-2-F	GGGAGACCCAAGCTGGCTAGC
HA-OTUD5-2-R	AACATCGTATGGGTAACCGGTCCCTGGTTTGAATGATGGC
HA-OTUD5-3-F	GGGAGACCCAAGCTGGCTAGC
HA-OTUD5-3-R	AACATCGTATGGGTAACCGGTGTCATTCAGACCAAAGGCA
HA-OTUD5-4-F	GGGAGACCCAAGCTGGCTAGCGCCACCGTCGGCGCAGGCTACAACA
HA-OTUD5-4-R	AACATCGTATGGGTAACCGGT
pGEX-4T-2-OTUD5-GST-F	TCGGATCTGGTTCCGCGTGGATCCATGACTATACTCCCCAAAAAGAAGCCGC
pGEX-4T-2-OTUD5-GST-R	TCGTCAGTCAGTCACGATGCGGCCGCACTCTTGTCTGGGGGCGGGT
pCDNA3.1-OTUD5-Myc-F	CCACACTGGACTAGTGGATCCGCCACCATGACTATACTCCCC
pCDNA3.1-OTUD5-Myc-R	TTTTTGTTCGGGCCCAAGCTTACTCTTGTCTGGGGGCG
pCDNA3.1-OTUD5-C224S-F	GATGAAGGAGGATGGCGCCTCTCTCTTCCGGGCTGTAGCTG
pCDNA3.1-OTUD5-C224S-R	CAGCTACAGCCCGGAAGAGAGAGGCGCCATCCTCCTTCATC
pCDNA3.1-OTUD5-K558A-F	TACCTAGACAGTATGGCGAAAAACAAAGTGCAC
pCDNA3.1-OTUD5-K558A-R	GTGCACTTTGTTTTTCGCCATACTGTCTAGGTA
pCDNA3.1-OTUD5-D555A-F	AACAGGAATACCTAGCCAGTATGAAGAAAAAC
pCDNA3.1-OTUD5-D555A-R	GTTTTTCTTCATACTGGCTAGGTATTCCTGTT
pCDNA3.1-OTUD5-E552A-F	CAGTGTCCCAACAGGCATACCTAGACAGTAT
pCDNA3.1-OTUD5-E552A-R	ATACTGTCTAGGTATGCCTGTTGGGACACTG
Flag-TIF1γ-1 (RBCC+M)-F	CTAGCTAGCTAGGCCACCATGGCGGAAAACAAA
Flag-TIF1γ-1 (RBCC+M)-R	GCTACCGGTAGCTTCATTTGGGTCATCATCTTTATT
Flag-TIF1γ-2 (RBCC)-F	CTAGCTAGCTAGGCCACCATGGCGGAAAACAAA
Flag-TIF1γ-2 (RBCC)-R	GCTACCGGTAGCTCCATTAGCAGCAGGGACAGGATC
Flag-TIF1γ-3 (M+PB)-F	CTAGCTAGCTAGGCCACCATGGCAATACGTTTC
Flag-TIF1γ-3 (M+PB)-R	GCTACCGGTAGCCTTTATATGTACTGGTCTCTCATC
Flag-TIF1γ-1043R-F	TTTAATGAAATGATGAGAGTTGTTCAAGTTTAT
Flag-TIF1γ-1043R-R	ATAAACTTGAACAACTCTCATCATTTCATTAAA
Flag-TIF1γ-1057R-F	CAAGAGATTAATTTGAGGGCTGATTCAGAAGTA
Flag-TIF1γ-1057R-R	TACTTCTGAATCAGCCCTCAAATTAATCTCTTG
Flag-TIF1γ-1127R-F	AGACCAGTACATATAAGGACCGGTGACTACAAG
Flag-TIF1γ-1127R-R	CTTGTAGTCACCGGTCCTTATATGTACTGGTCT
**Primers for qRT-PCR**	**Sequence, 5' – 3'**
TIF1γ-F	AGTGGCCATTTTCACCCTTATCA
TIF1γ-R	CATAACATGCTTCACCTGCCG
OTUD5-F	GGTTGTGCGAAAGCATTGCAT
OTUD5-R	ACCTCCACAGGACGGTTGT
OTUB1-F	TCGGTCCTATACAAGGAGTATGC
OTUB1-R	GGTCTTGCGGATGTACGAGT
OTUD7A-F	GTGTTGGGCAGCACTTCTACA
OTUD7A-R	CGTGGACCGAACAAAGTCTG
Snail-F	TCGGAAGCCTAACTACAGCGA

Snail-R	AGATGAGCATTGGCAGCGAG
Slug-F	TGTGACAAGGAATATGTGAGCC
Slug-R	TGAGCCCTCAGATTTGACCTG
PAI-1-F	AGTGGACTTTTCAGAGGTGGA
PAI-1-R	GCCGTTGAAGTAGAGGGCATT
β-actin-F	CACAGAGCCTCGCCTTTGCC
β-actin-R	CATGCCGGAGCCGTTGTCG
**Primers for ChIP**	**Sequence, 5' – 3'**
SBE1-F*	CGTCGTCGAGGTCCCA
SBE1-R	AGTAAGGGGCGGGGCT
SBE2-F	CACGTACTTCCGAGTAAG
SBE2-R	TCTCCAAGTCCAGCAA
SBE3-F	CACTGCCTTCATCTTGGA
SBE3-R	TAGTGTAGTGCGCGAGG
**shRNAs§**	**Sequence, 5' – 3'**
sh-OTUD5-1	CCGGGGGCTGGGCCTGCCATCATTCCTCGAGGAATGATGGCAGGCCCAGCCCTTTTTG
sh-OTUD5-2	CCGGGGACGAACCCATTCGTGTTAGCTCGAGCTAACACGAATGGGTTCGTCCTTTTTG
sh-TIF1γ	CCGGCGACATCCAGCAAGTTGGTGTCTCGAGACACCAACTTGCTGGATGTCGTTTTTG
sh-NC	CCGGTTCTCCGAACGTGTCACGTTTCTCGAGAAACGTGACACGTTCGGAGAATTTTTG
**siRNAs†**	**Sequence, 5' – 3'**
si-TIF1γ-1	CGACAUCCAGCAAGUUGGUTT
si-TIF1γ-2	AGUCAGGGCUCAGCAGCCUTT
si-SMAD4	GUACUUCAUACCAUGCCGATT
si-NC	UUCUCCGAACGUGUCACGUTT

* SBE, SMAD-binding element; F, forward; R, reverse.

§ shRNAs: short-hairpin RNAs.

† siRNAs: small interfering RNAs.

Table S5. Sequences for construction of luciferase reporter plasmids.

Fragments	Sequence, 5' – 3'
OTUD5 promoter (OP)<br>(-2,000 ~ -1, 2000bp)	ACTGGAAGTGAACTCCAAATACCTTCTTGGTTCATCAGCTCATCTGAACCC<br>CATAACCATTCCCTGAGGTAGTCTGGGCAGGTATTATCATTCCTGTTTTACA<br>CATGGGTAATCTCAAAAGTGACACAATGACTTGCTCAAGGTCATACAGTTG<br>GTAATGGTCCATCTGTGCCTGGAACTGAGGTCTTTCTGGCTCCAAAGCCTG<br>GGATCCTTCCACTGCACCACCATGCAGACTACTTTATTGAACACTTCCTATG<br>TGCTGGGCACAGCACCAGGCCCTTCACAAGCATTAATCTCTGATCCTCGGA<br>TTAGAATTATTGCCAATTTATAGAATAGAAATTAAGGATGGGTGCTCACTTC<br>GCACCACATATACTAAAATTAGAACGATACAGAGATTAGCATGGCACTATGC<br>GAAAGGATGACACGCAAATACGTGAAGCGTTCTATAAAAAAGTAAACAAC<br>AACAACAAAAATTGAGGACGGGGTGGGTTTTAAATGACTGACAAAGATCA<br>CTCAGACAGGAAGGGCAGAGCTAGGATTAGCGTGTAGCTCTCCAGCTACT<br>CAATAGCAGGGATTCTGCCAACCCCATTTTGAGACGCTGACTGGTGACAG<br>AGCTTTCATAAACCTCGATCGACCCAAGCAAACTCAGTTTTAGAAAAAGG<br>CTGGGCTTGGCCAGGCGCGGTGACTAACGCCTGTAATCCCAGCACTCTGG<br>GAAGCCGAGGCGGGAGGATCGCTTGAGCCCGGGAGCTGGAGATCAGCCT<br>GGGCAACATAGTGAGACTTTGTCCCTACAAAAAAGTGAAAAAAAAATTAG<br>CCGGTCGTAGTGGTGCGCGCCTGTGGTCCCAGCTATTCGGGAGGCTGAGG<br>CGGGAGGATCGCTTGAACCTGGGAGTTGGAGTTTGCAGTGAGCTATGATC<br>GCGCCACTGCATTCCAACCTGGGCGACAGAGTGAGACCCCTGTCTCAATT<br>AGGAAAAAAAAAAAAAAAAAAAAAAAAAAAGGCTGGGCCGCGCCTGCG<br>CACTAGCCATAGGGGCGGGGAGGTCAACGCGAGGCCGAGAGTGCTGTGG<br>TGCCAGAGAAGGCGTGTCTGTTTCCCAAACAACCACTGTCCCCACGGCGC<br>CTGTCTGTCCCAAGGTTGGTCGGTATGCCGCGAGTGACGGCGTCTCTTAAC<br>CAATCACCGCAGAACTCAGGCTGTTGCCGGGGCGAAGCGGTGTCCGCCTC<br>CCCCGGAAGGGTATTTGTAGGTGGGTGGGACCGGAAGTGACGCTACAGGG<br>GCCAGCTATGCTCCCGGGAGTGTTGATGTTTTCCAGTCATTCCGGCTGACA<br>GCGTTCAAGTTGGAATCCTGGAGGGGAGGTGTTTTTCCTGTCGTACGTGGG<br>ACAGGCCACGCTGTCCGTCCGCAGTACCGACGCCTGCAGGTCAGAGCTTC<br>GGGGAGAAAAGTGAAGAGCAAGACGGAACTGACGGGGAGAAAGGCTGG<br>GAACCAGGGTGTCGACTTTGACTGAAATTTGAGACGGAGGGCACCGGAG<br>GGCGAGCACTCGCCTGTGATTGGCCCGTGGGCGTCGTCGAGGTCCCACGC<br>AGCTGCTCAATTGGTTGGTGTTGCAATTGTTGTGGCGGCGGCGGCGGCGAT<br>TTTGCCCACGTACTTCCGAGTAAGGGGCGGGGCTGTGCCCTGGCGCGCGT<br>GCGCAGCGCCCCGGGGCCCCACCCGGTAGTGCAAGAACCTGCGAGGGGG<br>CGGAGCGAAGAGGTGCTTGTTTTGGTTCTGTTTCCTTTGAAGCAGAAGGC<br>CGGAACAAGCGTAGCAATAAACTTGCTGGACTTGGAGAGAAGGCTAAGAC<br>AAACTCGCCGCACTGCCTTCATCTTGGACTTTACATCCGGGTTCTCCTCTCG<br>GCGTGACCCGCGCGCCGCCACCGCCGCCGCCGCCGCCGCCTCCTCCG<br>CCGCCGAGGGTTCTCGAACCGGTGCCGCGATCCCTTTATCCGGGTCTCGCC<br>GTTCCCGTCGTGCCTCGCGCACTACACTAGCCCCCTCAT

OTUD5 promoter (OP)-WT<br>(-500 ~ -1, 500bp)	CACCGGAGGGCGAGCACTCGCCTGTGATTGGCCCGTGGGCGTCGTCGAGG<br>TCCCACGCAGCTGCTCAATTGGTTGGTGTTGCAATTGTTGTGGCGGCGGCG<br>GCGGCGATTTTGCCCACGTACTTCCGAGTAAGGGGCGGGGCTGTGCCCTG<br>GCGCGCGTGCGCAGCGCCCCGGGGCCCCACCCGGTAGTGCAAGAACCTGC<br>GAGGGGGCGGAGCGAAGAGGTGCTTGTTTTGGTTCTGTTTCCTTTGAAGC<br>AGAAGGCCGGAACAAGCGTAGCAATAAACTTGCTGGACTTGGAGAGAAG<br>GCTAAGACAAACTCGCCGCACTGCCTTCATCTTGGACTTTACATCCGGGTT<br>CTCCTCTCGGCGTGACCCGCGCGCCGCCACCGCCGCCGCCGCCGCCGCCG<br>CCTCCTCCGCCGCCGAGGGTTCTCGAACCGGTGCCGCGATCCCTTTATCCG<br>GGTCTCGCCGTTCCCGTCGTGCCTCGCGCACTACACTAGCCCCCTCAT
OTUD5 promoter (OP)-M#1<br>(-500 ~ -1, 500bp)	CACCGGAGGGCGAGCACTCGCCTGTGATTGGCCCGTGGGCGTCGTCGAGG<br>TCCCACGCAGCTGCTCAATTGGTTGGTGTTGCAATTGTTGTCCGCCGCCGG<br>GCGGCGATTTTGCCCACGTACTTCCGAGTAAGGGGCGGGGCTGTGCCCTG<br>GCGCGCGTGCGCAGCGCCCCGGGGCCCCACCCGGTAGTGCAAGAACCTGC<br>GAGGGGGCGGAGCGAAGAGGTGCTTGTTTTGGTTCTGTTTCCTTTGAAGC<br>AGAAGGCCGGAACAAGCGTAGCAATAAACTTGCTGGACTTGGAGAGAAG<br>GCTAAGACAAACTCGCCGCACTGCCTTCATCTTGGACTTTACATCCGGGTT<br>CTCCTCTCGGCGTGACCCGCGCGCCGCCACCGCCGCCGCCGCCGCCGCCG<br>CCTCCTCCGCCGCCGAGGGTTCTCGAACCGGTGCCGCGATCCCTTTATCCG<br>GGTCTCGCCGTTCCCGTCGTGCCTCGCGCACTACACTAGCCCCCTCAT
OTUD5 promoter (OP)-M#2<br>(-500 ~ -1, 500bp)	CACCGGAGGGCGAGCACTCGCCTGTGATTGGCCCGTGGGCGTCGTCGAGG<br>TCCCACGCAGCTGCTCAATTGGTTGGTGTTGCAATTGTTGTGGCGGCGGCG<br>GCGGCGATTTTGCCCACGTACTTCCGAGTAAGGGGCGGGGCTGTGCCCTG<br>GCGCGCGTGCGCAGCGCCCCGGGGCCCCACCCGGTAGTGCAAGAACCTG<br>GCTCCCCCGCCTCGCTAGAGGTGCTTGTTTTGGTTCTGTTTCCTTTGAAGC<br>AGAAGGCCGGAACAAGCGTAGCAATAAACTTGCTGGACTTGGAGAGAAG<br>GCTAAGACAAACTCGCCGCACTGCCTTCATCTTGGACTTTACATCCGGGTT<br>CTCCTCTCGGCGTGACCCGCGCGCCGCCACCGCCGCCGCCGCCGCCGCCG<br>CCTCCTCCGCCGCCGAGGGTTCTCGAACCGGTGCCGCGATCCCTTTATCCG<br>GGTCTCGCCGTTCCCGTCGTGCCTCGCGCACTACACTAGCCCCCTCAT
OTUD5 promoter (OP)-M#3<br>(-500 ~ -1, 500bp)	CACCGGAGGGCGAGCACTCGCCTGTGATTGGCCCGTGGGCGTCGTCGAGG<br>TCCCACGCAGCTGCTCAATTGGTTGGTGTTGCAATTGTTGTGGCGGCGGCG<br>GCGGCGATTTTGCCCACGTACTTCCGAGTAAGGGGCGGGGCTGTGCCCTG<br>GCGCGCGTGCGCAGCGCCCCGGGGCCCCACCCGGTAGTGCAAGAACCTGC<br>GAGGGGGCGGAGCGAAGAGGTGCTTGTTTTGGTTCTGTTTCCTTTGAAGC<br>AGAAGGCCGGAACAAGCGTAGCAATAAACTTGCTGGACTTGGAGAGAAG<br>GCTAAGACAAACTCGCCGCACTGCCTTCATCTTGGACTTTACATCCGGGTT<br>CTCCTCTCGGCGTGACCCGCGCGCCGCCAGGCGGCGGCGGCGGCGCGCCG<br>CCTCCTCCGCCGCCGAGGGTTCTCGAACCGGTGCCGCGATCCCTTTATCCG<br>GGTCTCGCCGTTCCCGTCGTGCCTCGCGCACTACACTAGCCCCCTCAT

Highlighted sequences in green, putative SMAD-binding elements; WT, wild type (green); M, mutant (red).
