# Supplementary material for: OTUD5-TIF1γ-SMAD3/4 positive feedback loop inhibits TGF-β-induced EMT and metastasis in NSCLC
Source: Cell Death Dis. 2026 May 25;17(1):650. doi: 10.1038/s41419-026-08901-z (PMC13385805; doi:10.1038/s41419-026-08901-z)

Source data for Figure 2

Fig. 2A

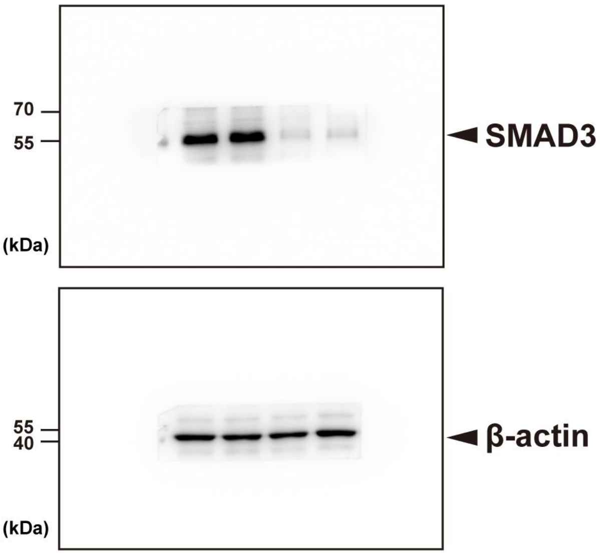

Fig. 2C

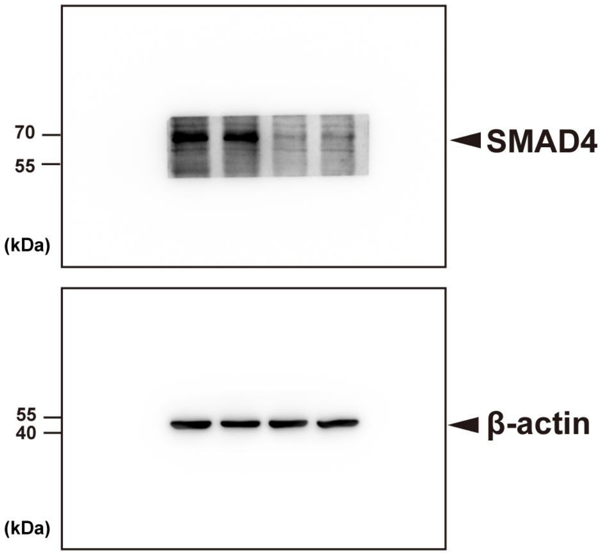

Source data for Figure 3

Fig. 3A

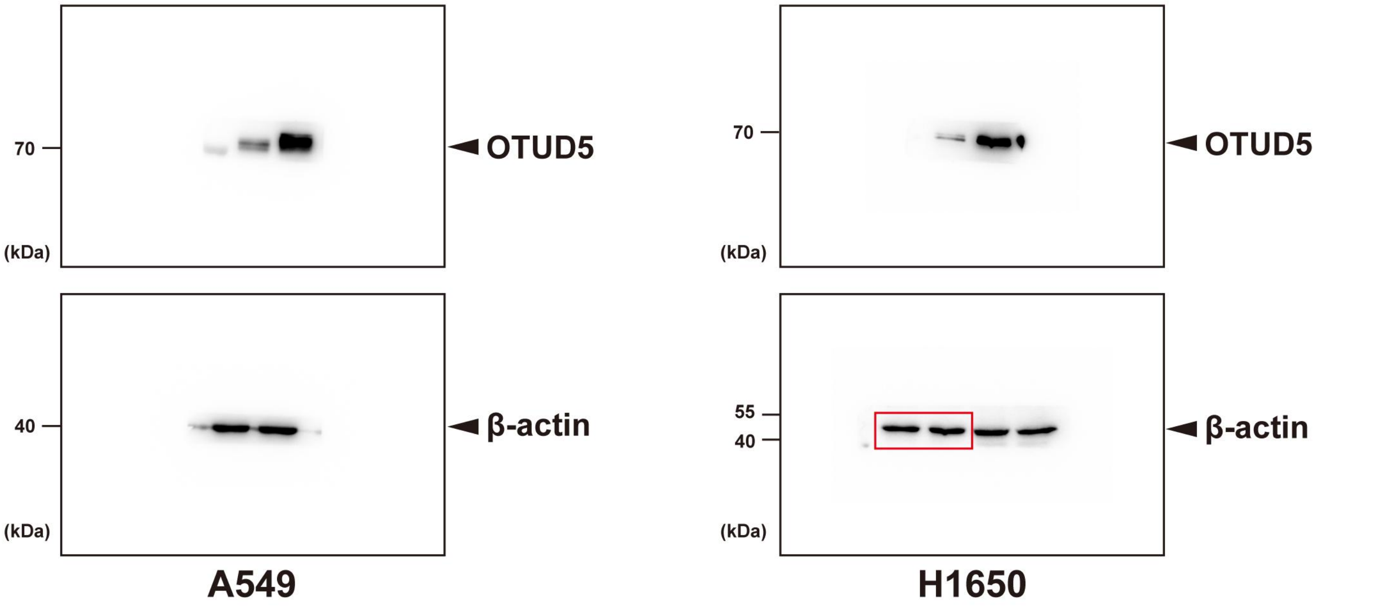

Fig. 3B

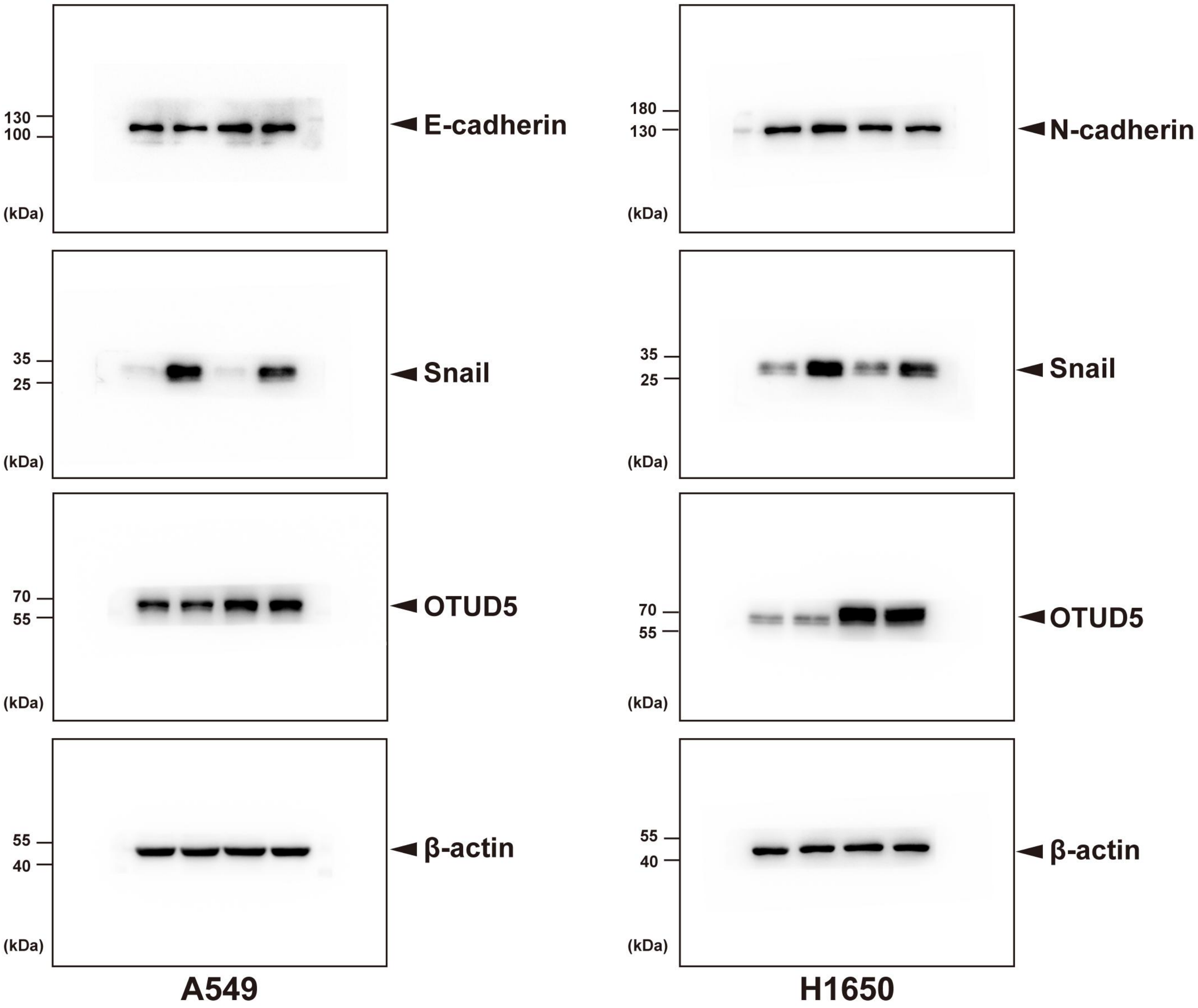

Source data for Figure 4

Fig. 4B

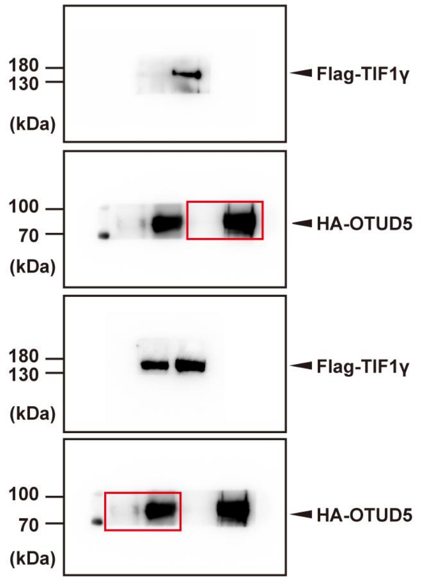

Fig. 4C

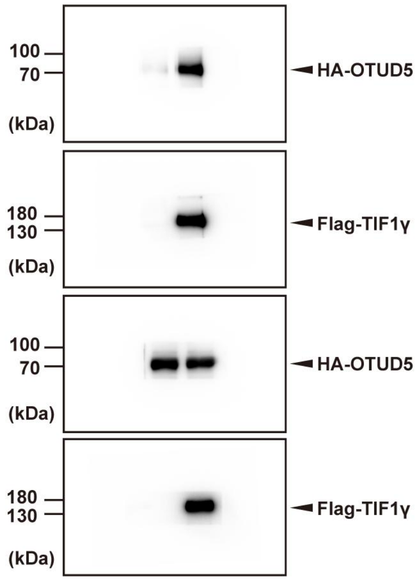

Fig. 4D

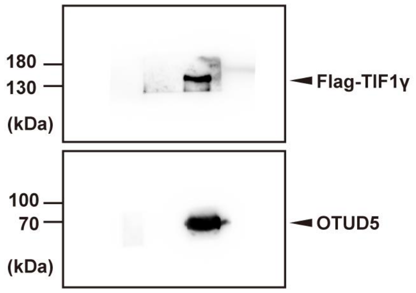

Fig. 4K

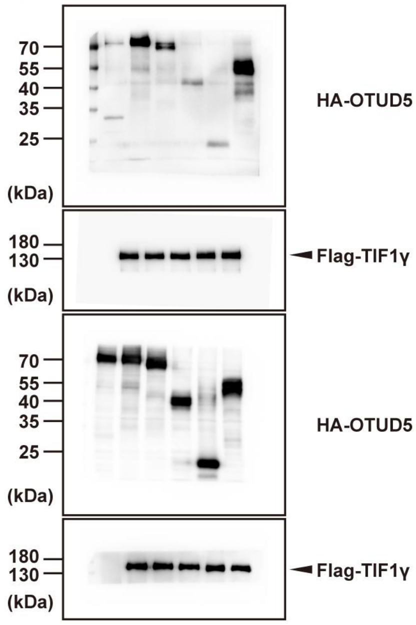

Fig. 4E

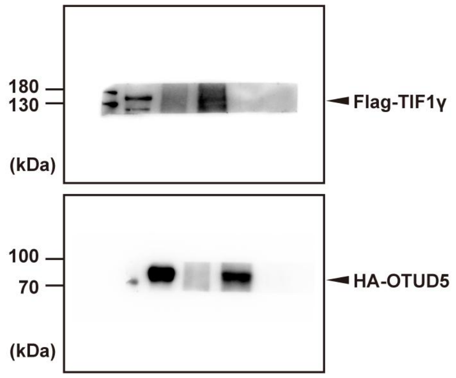

Fig. 4F

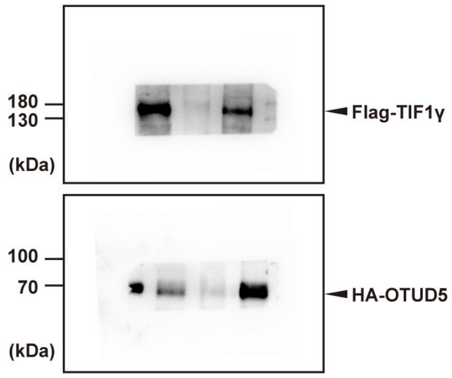

Fig. 4L

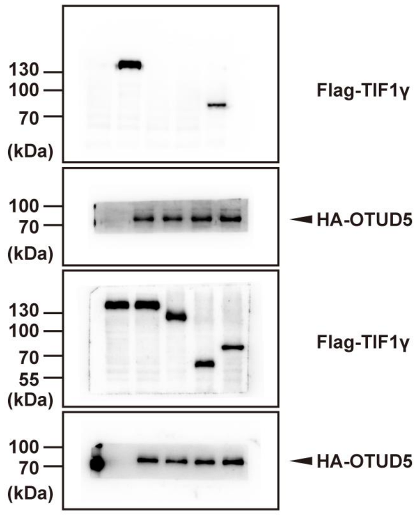

Fig. 4G

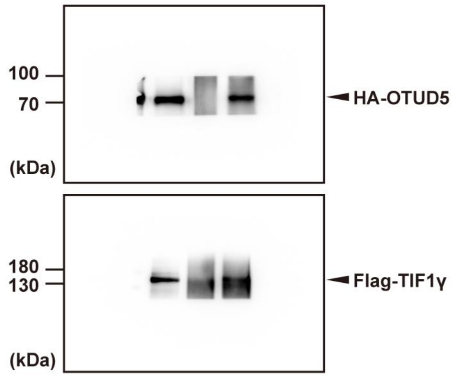

Fig. 4H

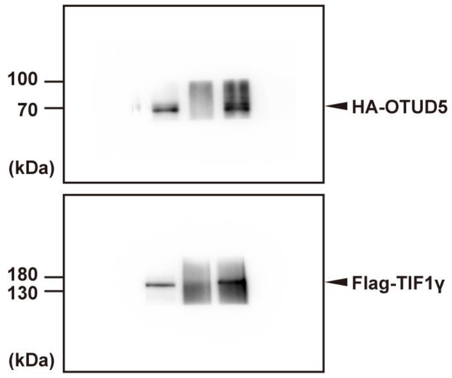

Source data for Figure 5

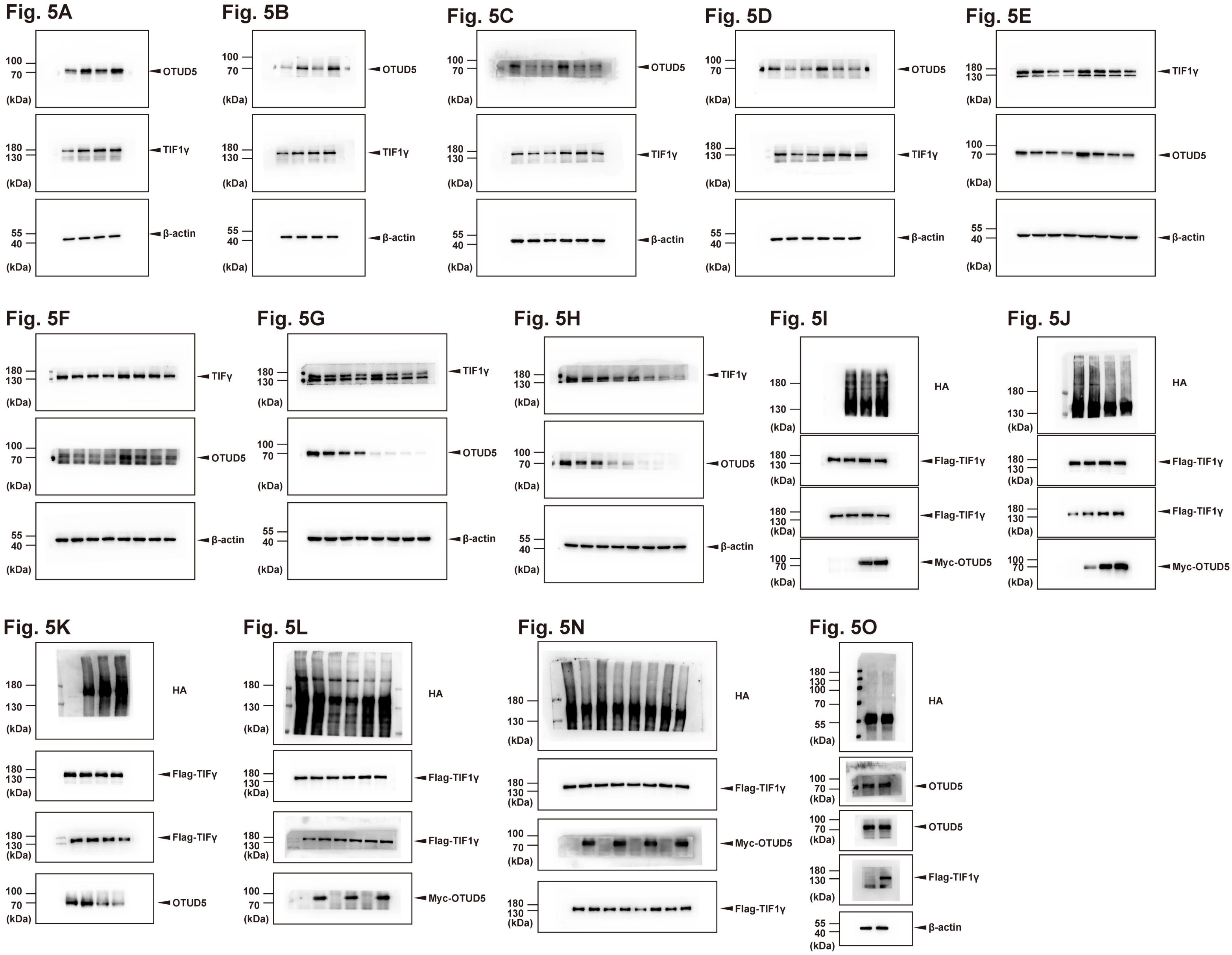

Source data for Figure 6

Fig. 6A

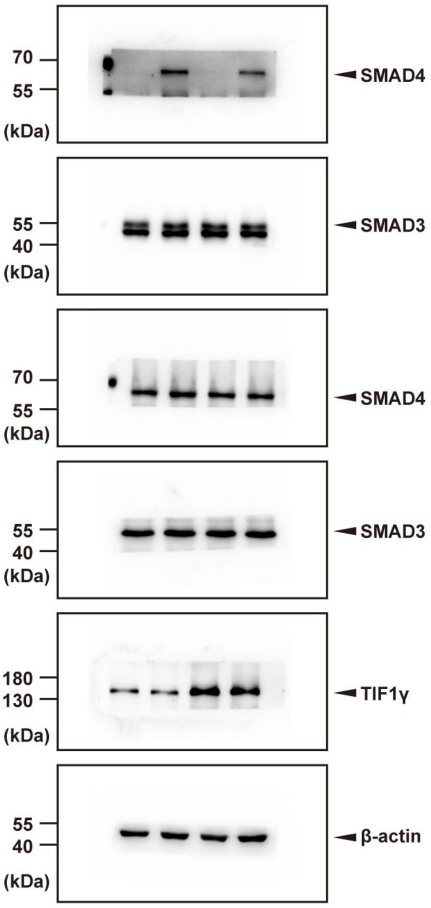

Fig. 6B

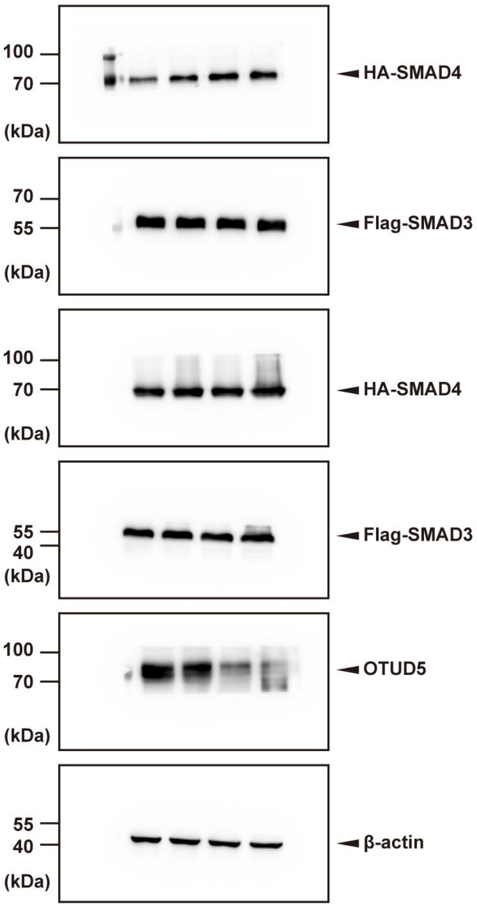

Fig. 6C

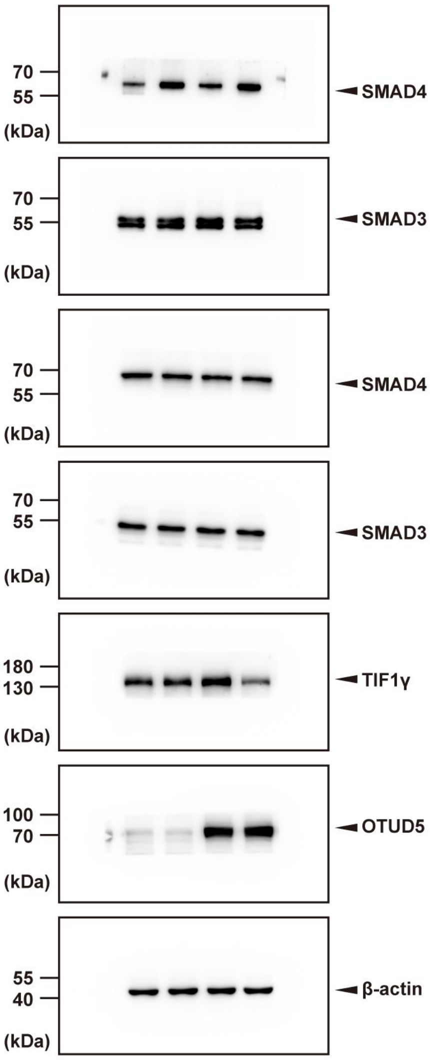

Fig. 6H

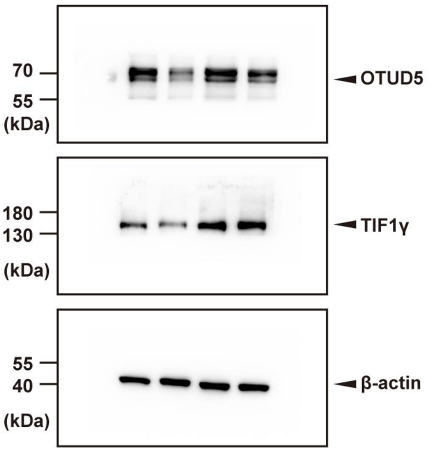

Source data for Figure 7

Fig. 7A

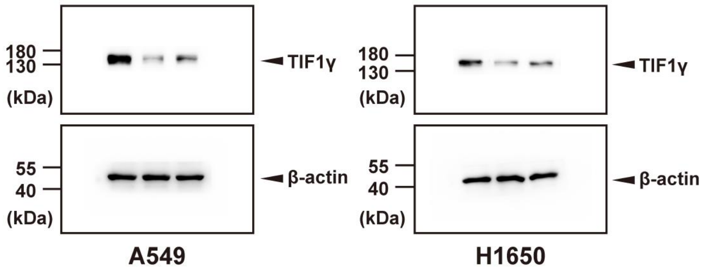

Fig. 7B

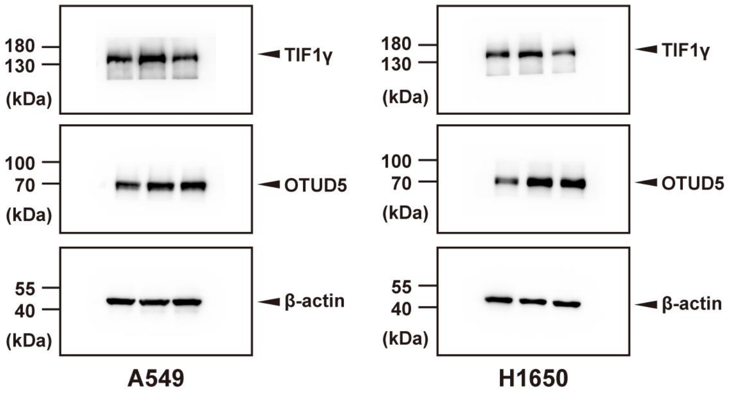

Fig. 7C

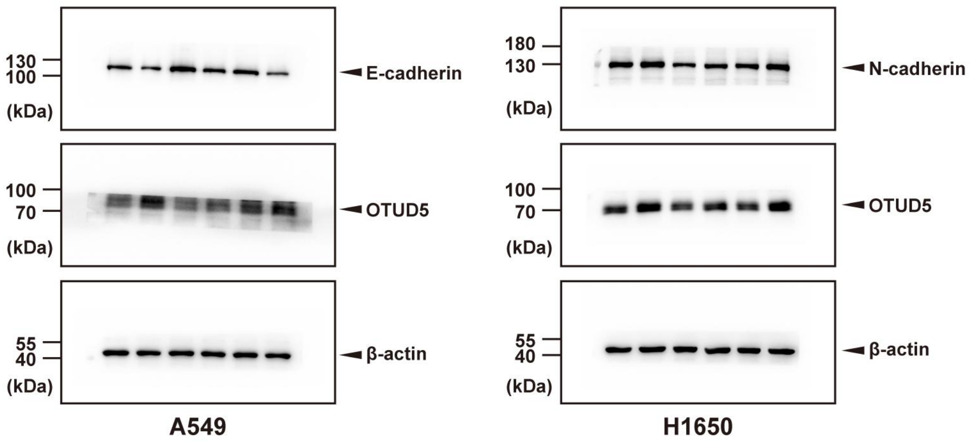

Fig. 7E

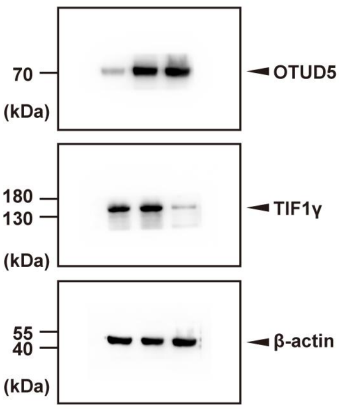

Source data for Figure 8

Fig. 8F

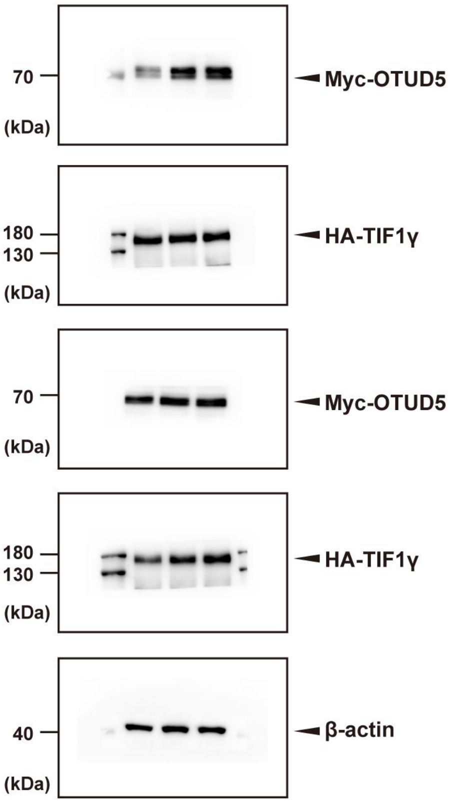

Fig. 8G

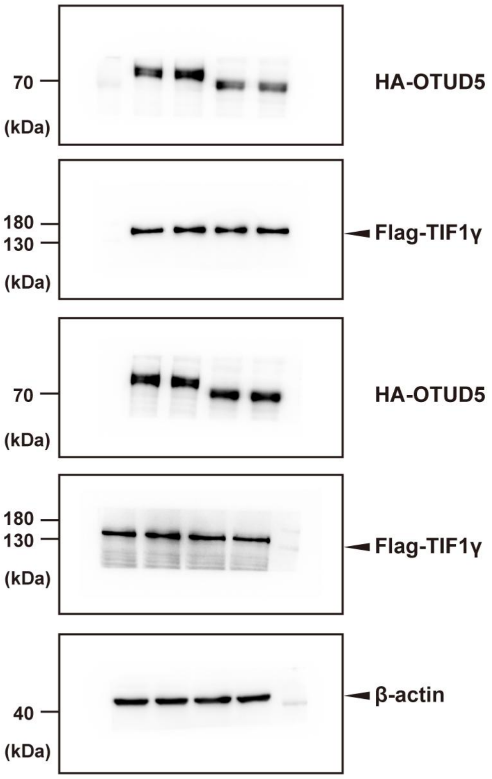

Fig. 8H

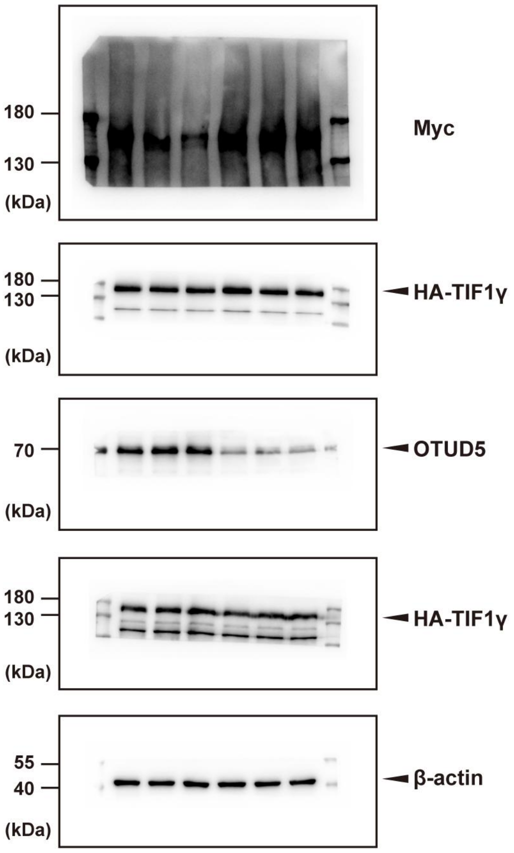

Fig. 8I

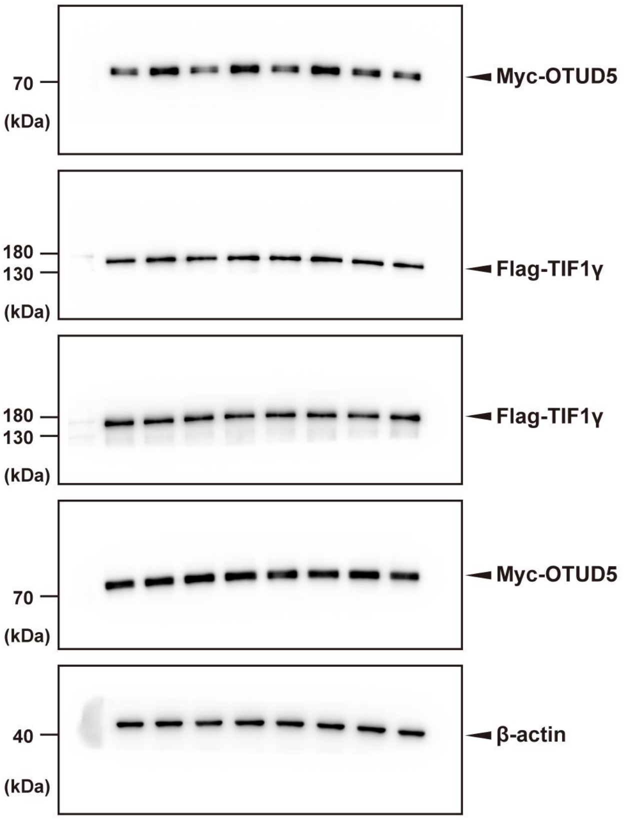

Fig. 8J

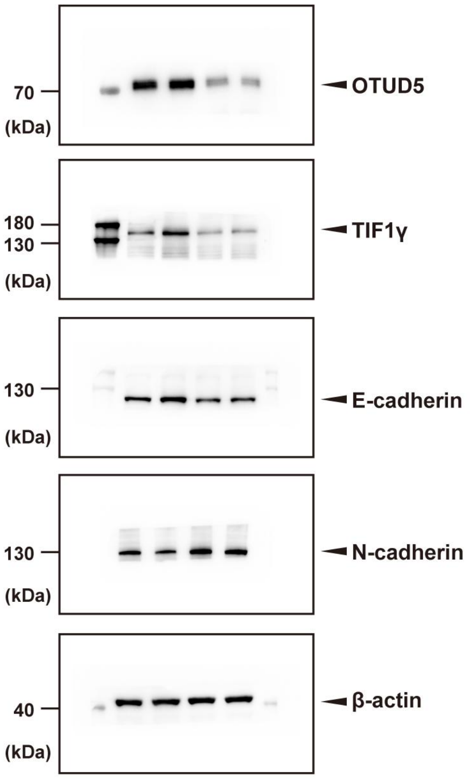

Source data for Figures S2-3

Fig. S2A

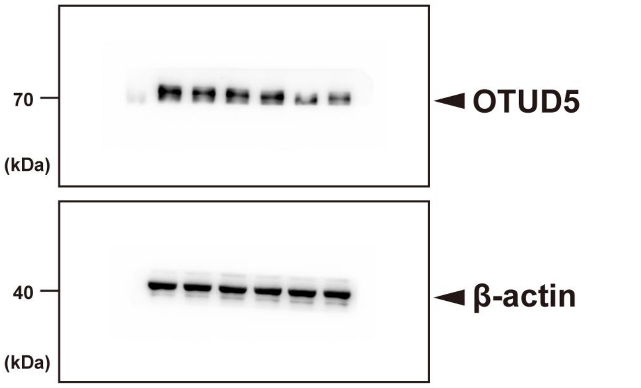

Fig. S2B

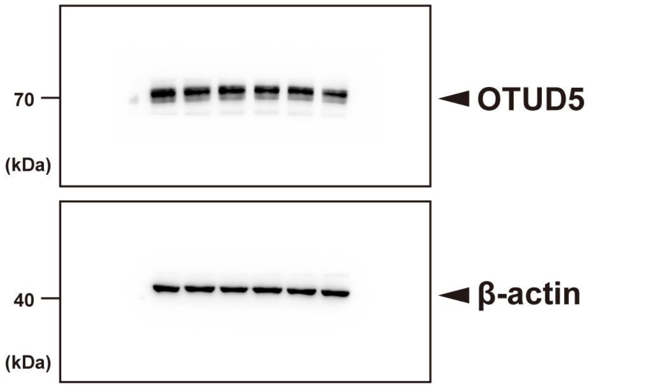

Fig. S3A

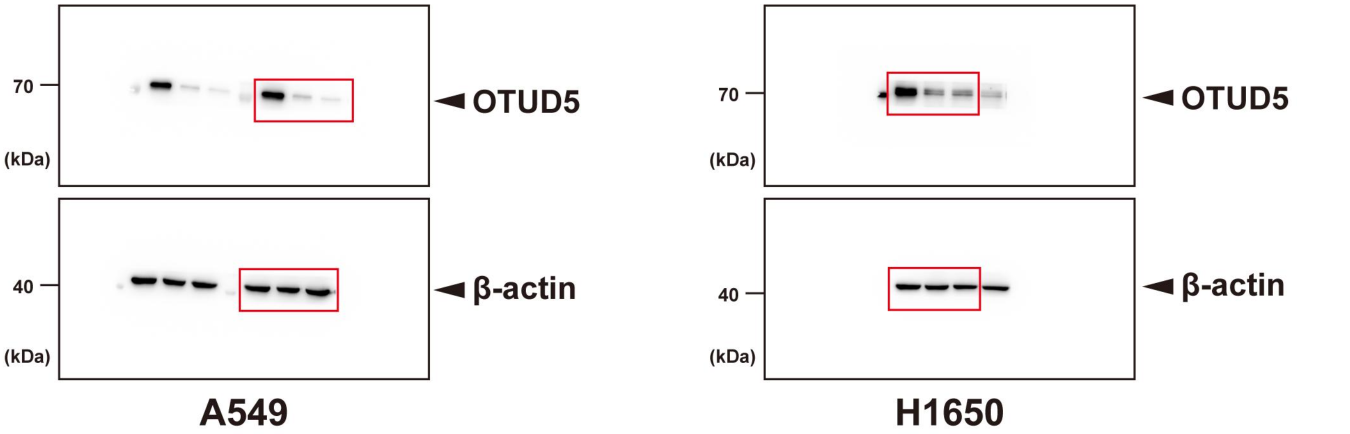

Fig. S3B

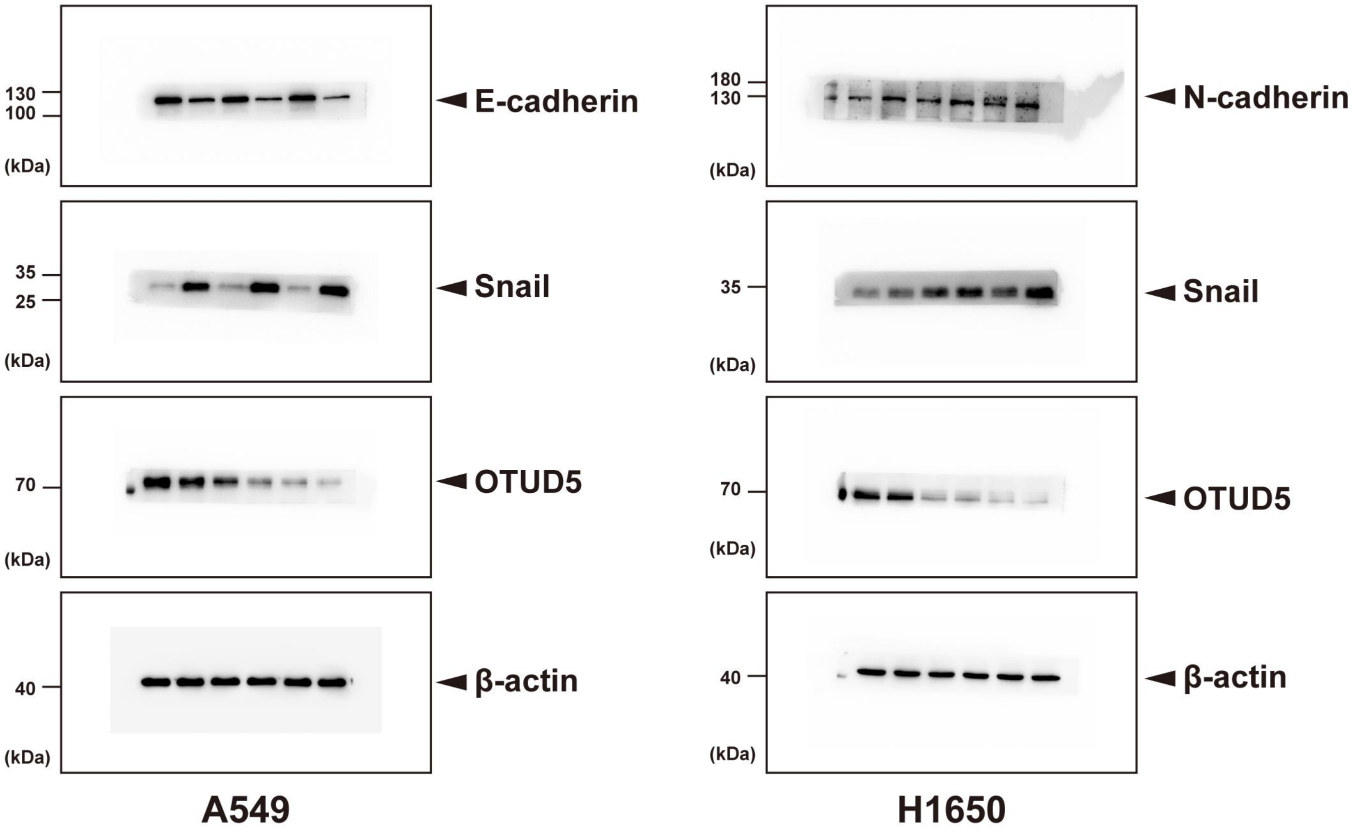

Source data for Figure S5

Fig. S5A

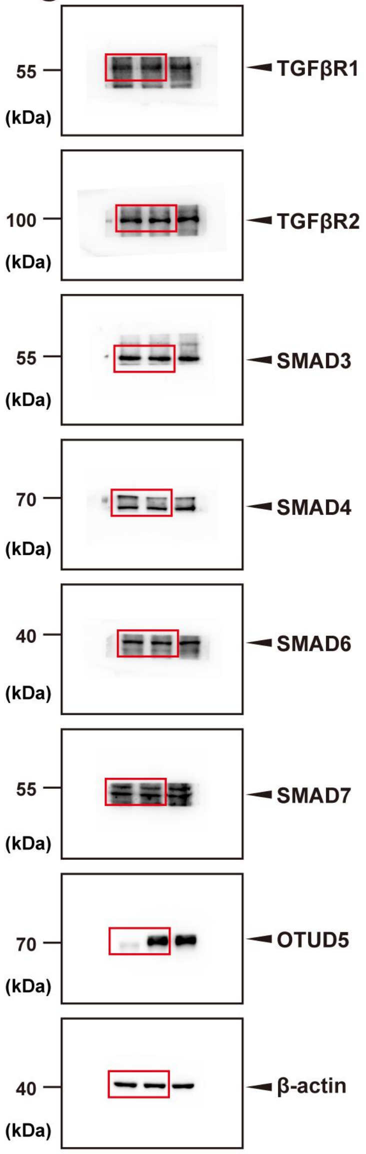

Fig. S5B

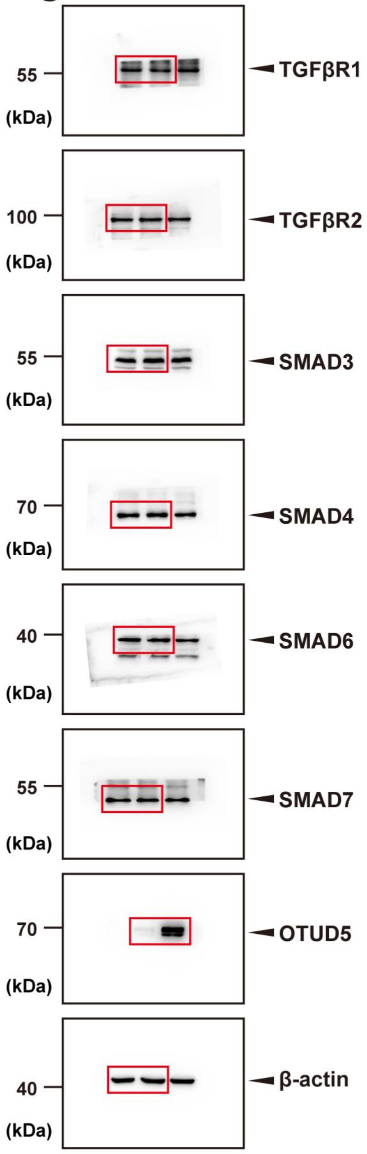

Fig. S5C

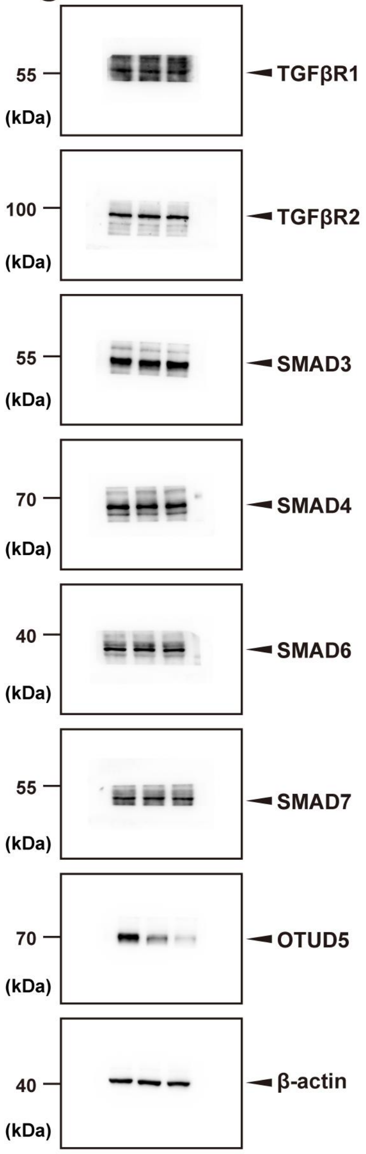

Fig. S5D

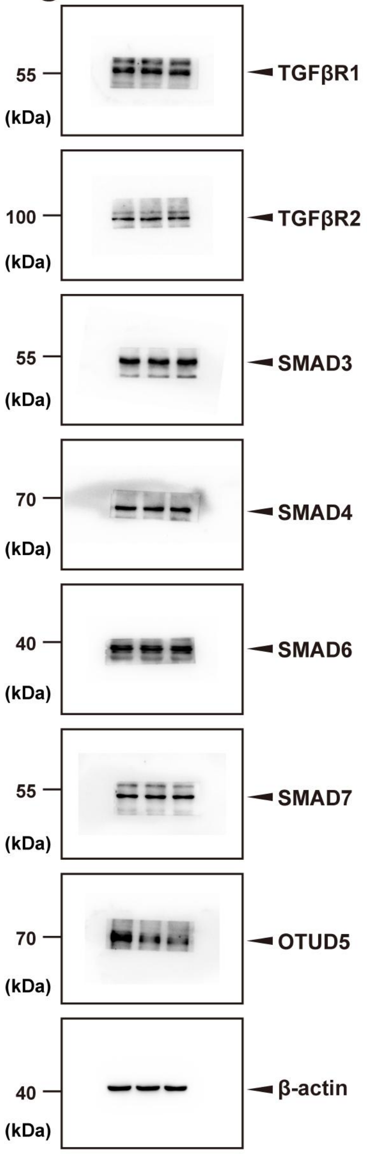

Source data for Figure S7

Fig. S7G

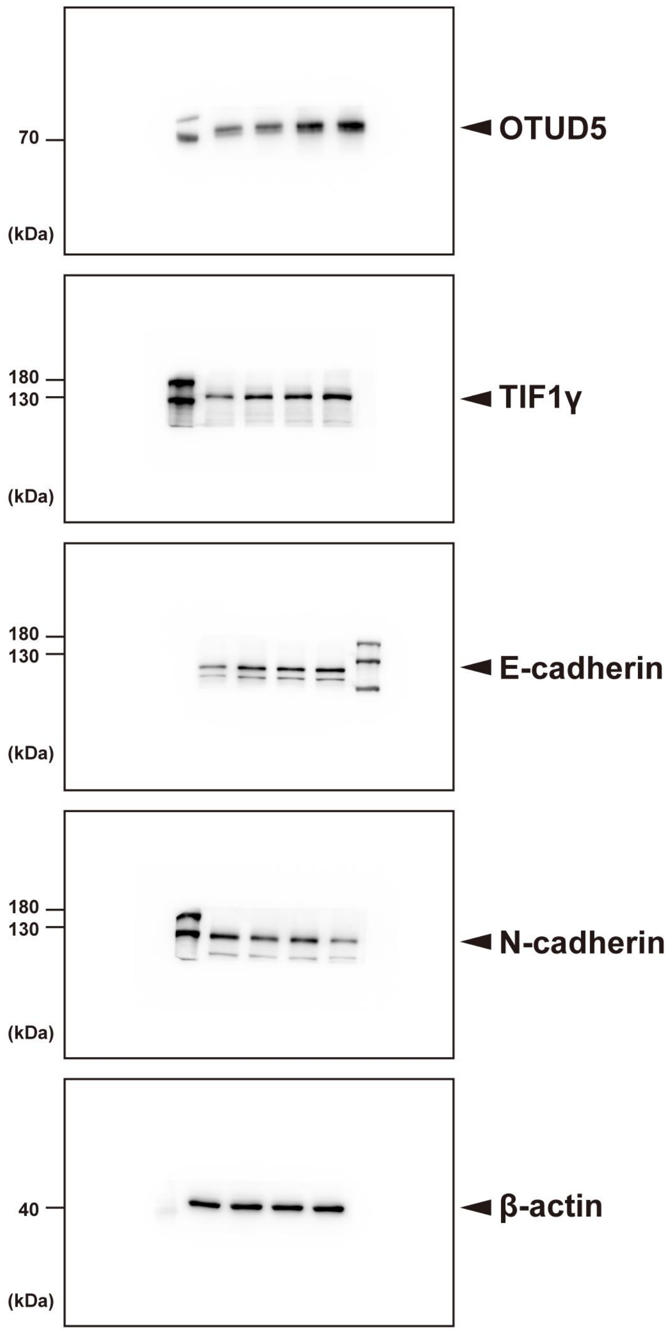

Supplement: Supplementary file 4 — Uncropped original western blots [file 41419_2026_8901_MOESM4_ESM.pdf]
